# Salidroside Ameliorates Depression by Suppressing NLRP3-Mediated Pyroptosis *via* P2X7/NF-κB/NLRP3 Signaling Pathway

**DOI:** 10.3389/fphar.2022.812362

**Published:** 2022-04-12

**Authors:** Yuhui Chai, Yawen Cai, Yu Fu, Yingdi Wang, Yiming Zhang, Xue Zhang, Lingpeng Zhu, Mingxing Miao, Tianhua Yan

**Affiliations:** ^1^ Department of Physiology and Pharmacology, China Pharmaceutic University, Nanjing, China; ^2^ Center of Clinical Research, The Affiliated Wuxi People’s Hospital of Nanjing Medical University, Wuxi, China; ^3^ Center of National Pharmaceutical Experimental Teaching Demonstration, China Pharmaceutic University, Nanjing, China

**Keywords:** salidroside, depression, pyroptosis, NLRP3, P2X7

## Abstract

Depression is a common and serious mental disorder. Data on its pathogenesis remain unclear and the options of drug treatments are limited. Here, we explored the role of pyroptosis, a novel pro-inflammatory programmed cell death process, in depression as well as the anti-depression effects and mechanisms of salidroside (Sal), a bioactive extract from *Rhodiola rosea L*. We established a corticosterone (CORT)-induced or lipopolysaccharide (LPS)-induced mice *in vivo*, and CORT, or nigericin (NLRP3 agonist)-induced PC12 cells *in vitro*. Our findings demonstrated that Sal profoundly mediated CORT or LPS-induced depressive behavior and improved synaptic plasticity by upregulating the expression of brain-derived neurotrophic factor (BDNF) gene. The data showed upregulation of proteins associated with NLRP3-mediated pyroptosis, including NLRP3, cleaved Caspase-1, IL-1β, IL-18, and cleaved GSDMD. The molecular docking simulation predicted that Sal would interact with P2X7 of the P2X7/NF-κB/NLRP3 signaling pathway. In addition, our findings showed that the NLRP3-mediated pyroptosis was regulated by P2X7/NF-κB/NLRP3 signaling pathway. Interestingly, Sal was shown to ameliorate depression *via* suppression of the P2X7/NF-κB/NLRP3 mediated pyroptosis, and rescued nigericin-induced pyroptosis in the PC12 cells. Besides, knock down of the NLRP3 gene by siRNA markedly increased the inhibitory effects of Sal on pyroptosis and proinflammatory responses. Taken together, our findings demonstrated that pyroptosis plays a crucial role in depression, and Sal ameliorates depression by suppressing the P2X7/NF-κB/NLRP3-mediated pyroptosis. Thus, our study provides new insights into the potential treatment options for depression.

## Introduction

Depression is a widespread mental health disorder, which is associated with disability and high mortality rates (Atique-Ur-Rehman and Neill, 2019). Typical clinical symptoms of depression include depressed mood, suppressed thinking and movement, as well as thoughts of worthlessness or even suicide (Su et al., 2018). It is a health problem that affects many people worldwide and leads to a significant social and economic burden (Malhi and Mann, 2018). Although multiple antidepressants are available for depression, there are still a series of limitations, including a low curative ratio, poor treatment compliance and serious side effects (Cosker et al., 2020). Thus, it is important to find more reliable treatments for depression.

Although the pathogenesis of depression is unclear, it is affected by various genetic, psychological, and physiological factors. Previous data showed that factors such as structural changes within the hippocampus, loss of BDNF, and excessive neuroinflammation are important in the development of depression (Haase and Brown, 2015). Recent evidence suggests that neuroinflammation is a key mediator of depression (Troubat et al., 2020). Other studies have shown that the expression of proinflammatory cytokines, especially interleukin (IL)-1β, is significantly enhanced in depressed patients (Pallavi et al., 2015). Increased inflammation leads to abnormal neuron changes and induces depressive symptoms (Felger 2018). Inflammation has been associated with a complex of NOD-like receptors (NLRs), and the NLRP3 receptor is an important molecular platform for processing and handling inflammatory responses in the intrinsic immune system (He et al., 2016). Activation of NLRP3 inflammasome releases inflammatory factors and initiates downstream inflammatory responses (Ramaswamy et al., 2013). Previous studies implicated that the NLRP3 inflammasome in the development of depression (Kaufmann et al., 2017; Su et al., 2017). Besides, other studies evaluated the IL-1β, TNF-α, and IL-6, and showed that they are overexpressed in patients with depression (Penninx et al., 2003; Thomas et al., 2005). Similarly, a previous systematic review and meta-analysis showed that the expression levels of IL-1β and IL-6 were significantly elevated in elderly patients with depression (Ng et al., 2018). In addition, there was significant increase of serum corticosterone and IL-1β, as well as higher levels of NLRP3 protein in hippocampus in the chronic stress-induced depression mice models (Zhang et al., 2015). Thus, NLRP3 inflammasome plays a crucial role in depression.

Previous data has shown that activation of the NLRP3 inflammasome can trigger caspase-1 associated pyroptosis, also referred to as “caspase-1 dependent programmed cell death”. Pyroptosis executor gasdermin D (GSDMD) is cleaved by activated caspase family proteins and releases the N-terminal, which creates holes in the cell membrane, leading to enhanced release of inflammatory products, cell rupture and death (Jorgensen et al., 2017). Recent studies have shown that NLRP3-regulated pyroptosis is involved in various neurodegenerative and metabolic disorders, such as Parkinson’s disease and diabetes as well as depression (Wang et al., 2019; Guan and Han, 2020; Li et al., 2020). However, data on the underlying mechanisms remain unclear.

The P2X7 receptor is distributed on cell membranes and is a P2X subtype closely associated with depression. After exposure to stressors, large amounts of ATP is released, which leads to activation of the P2X7R and NLRP3-mediated inflammatory responses (Bhattacharya and Jones, 2018). The release of inflammatory factors triggers neuroinflammation which can further lead to depressive-like behaviors. Besides, activated P2X7R can activate nuclear factor-κB (NF-κB) and promote maturation and excretion of inflammatory factors, such as IL-8 and IL-1β (Korcok et al., 2004; Thawkar and Kaur, 2019; Wang D. et al., 2020). In addition, it was shown that P2X7R, a key inflammatory switch can activate the NLRP3 inflammasome (Adinolfi et al., 2018).

Salidroside (Sal), a kind of phenylpropanoid glycoside found in the herb *Rhodiola rosea* L., has various pharmacological effects, including anti-inflammatory, antioxidant, and regulation of cognitive functions (Jin et al., 2016; Hu et al., 2020). Recent research has reported that Sal has protective effects on various diseases such as Alzheimer’s disease, epilepsy and stroke (Wang et al., 2015; Zhang et al., 2018; Wang H. et al., 2020). Data has shown that Sal exerts a potent antidepressant-like effect in chronic stress-depressed models or despair models and promotes neurogenesis in the hippocampus (Panossian et al., 2008; Vasileva et al., 2018). Although these findings demonstrate the ameliorative effect of Sal on depressive symptoms, data on its specific molecular mechanisms of Sal remain scant. This study aimed to explore the mechanisms of depression and the potential antidepressant-like effects of Sal.

## Materials and Methods

### Materials

Corticosterone (CORT, C104537), Fluoxetine (FLU, F131623) and TWEEN^®^ 80 (T118633) were purchased from Aladdin (Shanghai, China). Lipopolysaccharide (LPS, L2630) was purchased from Sigma Aldrich (St. Louis, United States). Salidroside (Sal, MB5843-1) was purchased from Meilunbio (Dalian, China). Nigericin (Nig, HY-127019) was purchased from MedChemExpress (NewJersey, United States). The primary antibodies against P2X7, ASC, IL-18, IL-1β were purchased from Proteintech Group (Proteintech, United States). All chemicals and reagents were summarized in Supplementary Table S1.

### Animals

The male C57BL/6 mice (8 weeks old, 20–22 g) were acquired from the Qinglongshan Farm (NO. SCXK 2020–0,005, Nanjing, China). To avoid the influence of female hormones on depressive behaviors, male mice were used in our study (Newhouse et al., 2015). The experimental animals were housed in cages with free access to food and water at the Laboratory Animal Research Center of China Pharmaceutical University. The laboratory temperature was controlled at 20–22°C with 50%–60% relative humidity, with a 12-h light-dark cycle. All animals are acclimatized to the laboratory environment for 1 week before the experiment. The protocol of experiments was supported by the Ethics Committee of China Pharmaceutical University.

### Experimental Design

#### Experiment I: Effect of Sal on CORT-Induced Depression

Male C57BL6/J mice were weighed before the experiment, and were randomly divided into five groups (*n* = 10 per group): 1) the control group, 2) the CORT-injection group, 3) the CORT + fluoxetine treatment group (CORT + FLU), 4) the CORT +20 mg/kg Sal [CORT + Sal (20 mg/kg)] group, 5) the CORT +40 mg/kg Sal [CORT + Sal (40 mg/kg)] group. CORT was suspended in physiological saline containing 0.1% DMSO and 0.1% Tween-80, and the suspension was administered through subcutaneous injection (20 mg/kg) at a volume of 10 ml/kg once daily for 21 days to induce depression-like symptoms (Cavalcanti Capibaribe et al., 2019; Yokoyama et al., 2020). Based on the available studies, FLU (20 mg/kg) was selected for this experiment (Li et al., 2021). To determine the optimal dose of Sal, we relied on previous studies (Chen et al., 2012; Palmeri et al., 2016). Except control group, the other four groups all received CORT injection for 21 days. Sal and FLU were dissolved in saline and administrated by oral gavage 30 min prior to the CORT injection for 21 days. After 24 h of the last CORT administration, behavioral tests were conducted according to the time sequence of administration, including OFT, SPT, and FST. The experimental procedure is shown in .Supplementary Figure S1A. Subsequently, some mice were sacrificed and hippocampal tissue was stored at −80°C until further assays. The brain of other mice was removed and immersed in 4% PFA at 4°C for immunohistochemical examination and HE staining.

#### Experiment Ⅱ: Effect of Sal on LPS-Induced Depression

Mice were randomly divided into five groups (*n* = 10 per group): 1) the control group, 2) the LPS treated group, 3) the LPS +20 mg/kg FLU (LPS + FLU) group, 4) the LPS +20 mg/kg Sal [LPS + Sal (20 mg/kg)] group, 5) the LPS + 40 mg/kg Sal [LPS + Sal (40 mg/kg)] group. LPS (1 mg/kg) was dissolved in saline and injected intraperitoneally (10 ml/kg) for 5 days to induce acute depressive symptoms (Ali et al., 2020). Sal and FLU were dissolved in saline and administrated by oral gavage 30 min prior to the LPS injection for 5 days. Mice treated with a vehicle for the same period were used as the control group. The behavioral evaluations were given after the final LPS administration. The schedule of the experimental design was shown in Supplementary Figure S1B. Finally, some mice were sacrificed and hippocampal tissue was stored at −80°C until further assays. Another was sacrificed and the brain was fixed in 4% PFA at 4°C for immunohistochemical examination.

### Behavioral Tests

The behavioral tests were conducted on consecutive 3 days in the following description order. The first one was the open field test (OFT), followed by the sucrose preference test (SPT) and the last one was the forced swim test (FST).

#### Open Field Test

Based on previous studies, moderately modified OFT was used to assess the locomotor capacity of mice (Zhao et al., 2019). Briefly, mice were acclimated to the experimental room for 1 h and were placed in a wooden box (45 × 45 × 45 cm), which was defined into 25 small squares. We artificially divide the nine grids as the central area and the remaining 16 squares against the walls as the peripheral area. The video recording system (ANY-Maze animal behavior analysis system) automatically records the locomotor activity of mice for 5 min. The observation index including distance, time and average velocity. After the test of each mouse, 75% alcohol was used to clean up the odor of the previous mouse and avoid affecting the results of the next experiment.

#### Sucrose Preference Test

The absence of the ability to feel pleasure from pleasant activities is a classic symptom of human depression. On the other hand, depressed animals showed a decreased rate of preference for sucrose solution intake. The sucrose preference test was conducted using two bottles of either containing 1% sucrose solution or water respectively. Mice were acclimated to sucrose solution for 24 h. For the next 24 h, 1% sugar water was replaced by water. After the mice were starved of food and water for 24 h, the animals housed solely and had free access to the two bottles containing either 1% sucrose solution or water during the formal experiments. After 24 h, the ratio of sucrose consumption and total fluid intake was calculated as a sucrose preference ratio (Couch et al., 2016).

#### Forced Swim Test

Mice were subjected to FST as noted in previous studies (Porsolt et al., 1977). Mice were placed in a beaker (height 21 cm, diameter 14 cm) filled with 15 cm depth of water (20–22°C). The total immobile time for the last 4 minutes of the 6-min total duration was calculated and analyzed by an observer blinded to the treatments.

### Molecular Docking

To further explore the potential mechanism of Sal-mediated antidepressant related to pyroptosis processed by P2X7, molecular docking analysis was performed. The P2X7 protein (PDB code: 6U9W) structure was downloaded from the RCSB website (http://www.pdb.org) in PDB format. The 3D structure of Sal and A804598 (a typical P2X7R antagonist) was downloaded from https://pubchem.ncbi.nlm.nih.gov/. The P2X7 receptor was pretreated with pyMOL software to remove small molecule ligands, dehydrate, and hydrogenate. Then, both the small molecule and protein receptor formats are converted to PDBQT format. Finally, AutoDock Vina 1.1.2 was employed for molecular docking (Seeliger and de Groot, 2010).

### Cell Culture

The PC12 cells were cultured in DMEM (C11995500BT, Gibco) with 10% FBS, and placed in an incubator at 5% CO_2_ and 37°C. The concentration of CORT and Sal was selected by the CCK-8 assay. The cells were treated with CORT, and used to simulate an *in vitro* experimental model of depression in our study. To verify the neuroprotective benefits of Sal, the PC12 cells were exposed to Sal for 2 h and then treated with CORT (200 μM) for 24 h.

To determine whether NLRP3 can activate CORT-induced pyroptosis, the NLRP3 agonist nigericin (10 μM) was used in this study. The PC12 cells were divided into six groups for different treatments: 1) the Control group; 2) the 200 μM CORT (CORT) group; 3) the 10 μM nigericin (Nig group); 4) the 200 μM CORT +50 μM Sal (CORT + Sal) group; 5) the 10 μM Nig +50 μM Sal (Nig + Sal) group; 6) the 200 μM CORT +50 μM Sal +10 μM nigericin (CORT + Sal + Nig) group. The cells were treated with Sal (50 μM) for 2 h, and then incubated with CORT (200 μM) or Nig (10 μM) for 24 h.

### Cell Transfection

NLRP3 siRNA and NC (negative control) siRNA provided by RiboBio (Guangzhou, China) were used in our study. The nucleotide sequences of siRNAs are listed in Supplementary Table S2. The PC12 cells were divided into six groups: the control group, the CORT group (200 μM), the Si-NC (100 nM) + CORT group, the Si-NLRP3 (100 nM) + CORT group, the CORT + Sal (50 μM) group, the Si-NLRP3 (100 nM) + CORT + Sal (50 μM) group. The PC12 cells were transfected with siRNAs by using riboFECTTMCP Reagent according to the manufacturer’s instructions. After transfection for 24 h, the cells were stimulated with CORT (200 μM) or Sal (50 μM) for 24 h. Finally, the cells were collected for various analysis.

### Measurement of Cell Viability

The cell viability was quantified by the CCK-8 kit according to the manufacturer’s protocol. In brief, 10 µl of CCK-8 solution was added to each well of the 96-well plate and cultivated at 37°C for 1 h without light. The absorbance at 450 nm was detected by an enzyme marker. The cell viability of each well was computed according to the formula (Li L. et al., 2016): Cell Viability (%) = (A_test–_A_blank_)/(A_control–_A_blank_)*100%. The absorbance of different samples represented by A_test_, absorbance of unprocessed cells is expressed as A_control_, and A_blank_ is the absorbance of the well that is not inoculated with cells.

### Lactate Dehydrogenase Release Assay

LDH, mainly located in the cytoplasm, can be released into the extracellular when cell membranes are disrupted (Sun et al., 2020). The release of LDH was determined by the LDH detection kit (Beyotime, China), according to the manufacturer’s instructions. Cytotoxicity was determined by a microplate reader at 490 nm.

### Calcein-AM/Propidium Iodide Staining

The cell membrane integrity was detected by Calcein/PI Cell Viability/Cytotoxicity Assay Kit (Beyotime, China) according to the manufacturer’s instructions. Briefly, the cells were seeded in 24-well plates, then stained with Calcein-AM and PI mixed solution at 37°C for 30 min. The images were obtained using a fluorescent microscope (Olympus, Japan) (living cells appeared green and dead cells displayed red fluorescence) and the percentage of PI-positive cells was quantified by ImageJ software.

### Enzyme-Linked Immunosorbent Assay Analysis

ELISA kits were used to determine the levels of BDNF and inflammatory cytokines (IL-18 and IL-1β) in our study according to the protocol’s guidelines.

### Hematoxylin-Eosin Staining

Brain samples were fixed in 4% paraformaldehyde and embedded in paraffin for histopathological analysis (Khan et al., 2020). Serial 5 µm thick sections were obtained at the level of the hippocampus (Shal et al., 2019). HE staining to detect the morphological changes of the hippocampus.

### Immunohistochemistry Staining

Immunohistochemistry was carried out as previously reported with minor modifications (Aricioglu et al., 2019). Brain tissue was fixed with 4% paraformaldehyde for 24 h. Dehydrated, embedded in paraffin, and cut into slices of 5 µm thickness. After a series of operations, including deparaffinization, antigen repair and blocking, sections were incubated overnight with primary antibodies: anti-BDNF (Abcam, ab108319, 1:200), anti-P2X7 (Proteintech, 28207-1-AP, 1:50), anti-NLRP3 (Abcam, ab214185, 1:200) and anti-Cleaved caspase-1 (Thermo Fisher, PA5-99390, 1:200) at 4°C, followed by secondary antibodies incubation. Then, the slices were colored with diaminobenzidine (DAB). Finally, after dehydration and drying, sections were observed using a light microscope. The density values are analyzed by Image pro-plus software.

### Immunofluorescence

The expressions of P2X7, NLRP3 and Cleaved caspase-1 in PC12 cells were evaluated via immunofluorescence assay. Immunofluorescence was carried out as previously reported, with slight modifications (McFerran et al., 1998). In brief, PC12 cells were cultured on 24-well cell plates, fixed by 4% paraformaldehyde for 15 min. Cells were permeabilized with 0.1% Triton × 100 for 20 min and the blocking by 5% BSA for 30 min. The cells were incubated with the antibodies: anti-P2X7 (Proteintech, 28207-1-AP, 1:400), anti-NLRP3 (Abcam, ab214185, 1:400), Cleaved caspase-1 (Thermo Fisher, PA5-99390, 1:400) overnight at 4°C. The next day, the cells were washed three times in PBS followed by further incubation with a fluorescence-conjugated antibody for 2 h at room temperature. The nuclei were counterstained with DAPI. The images were photographed by inverted fluorescence microscopy and quantified by ImageJ software.

### Western Blot Analysis

The hippocampus tissues and cells were homogenized in cold RIPA buffer containing various protease inhibitors. The equal amounts of protein were loaded and separated by 12% SDS-PAGE gels. After being transferred to PVDF and blocked by 5% skim milk powder for 2 h, and the membranes were incubated with antibodies targeting BDNF (Abcam, ab108319, 1:1000), P2X7 (Proteintech, 28207-1-AP, 1:1000), ASC (Proteintech, 10500-1-AP, 1:1000), NF-κB (CST, #8242, 1:1000), P-NF-κB (CST, #3033, 1:1000), NLRP3 (Abcam, ab214185, 1:1000), IL-18 (Proteintech, 10663-1-AP, 1:1000), IL-1β (Proteintech, 16806-1-AP, 1:1000), Cleaved GSDMD (CST, #10137, 1:1000), Cleaved caspase-1 (Thermo Fisher, PA5-99390, 1:1000), β-actin (Proteintech, 66009-1-AP, 1:5000) at 4°C overnight. After washing with TBST, the protein bands were incubated with the HRP-binding secondary antibody (1:1000) for 1 h at room temperature. Finally, blots were displayed with ECL reagent and quantified with ImageJ software.

### Statistical Analysis

Results are expressed as mean ± SEM. *p* < 0.05 means that the difference is statistically significant. All statistical analyses were analyzed by GraphPad Prism eight software. Statistical significance of various groups was analyzed by one-way ANOVA followed by Tukey’s test.

## Results

### Sal Ameliorates CORT-Induced Depression

#### Sal Attenuates CORT-Induced Depression-Like Behaviors

**FIGURE 1:**
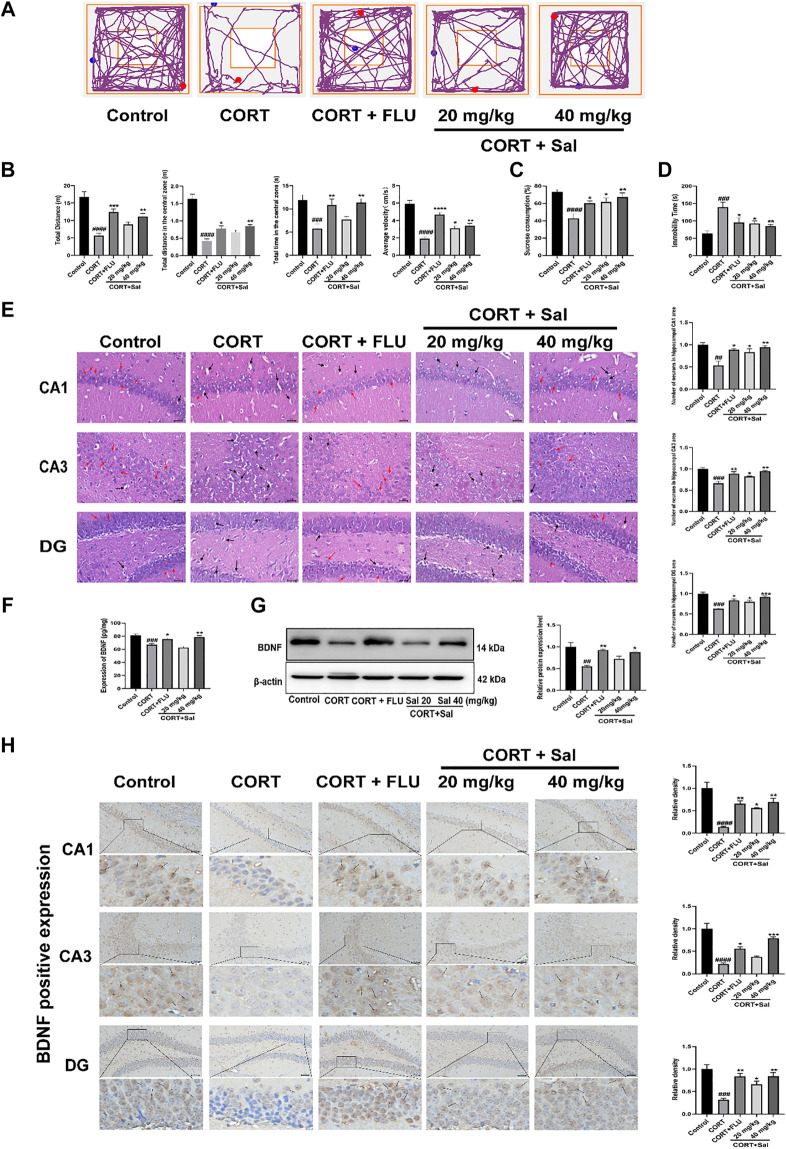
Effects of Sal in CORT-induced depression in mice. **(A)** Representative tracks of different experimental groups in OFT. **(B)** Sal improved locomotor activity in the OFT (*n* = 10). **(C)** Sal ameliorated the consumption of sucrose solution in the SPT (*n* = 10). **(D)** Sal decreased the immobility time in the FST (*n* = 5). **(E)** HE staining to evaluate the pathological changes in the hippocampus. Cells have pyknotic nuclei with vacuolated cytoplasm (black arrows) and normal neuronal cells (red arrows) (magnification: × 400, Scale bar = 25 μm, *n* = 3). **(F)** The levels of BDNF in the hippocampus was examined using ELISA kits (*n* = 5). **(G)** Sal improved CORT-induced the decrease of BDNF in the hippocampus was determined by Western blot (*n* = 3). **(H)** Immunochemical staining and the relative density of BDNF in hippocampus CA1, CA3, and DG. Brown staining represents the expression of BDNF (black arrows). Original magnification: × 200, *n* = 3. ^####^ *p* < 0.0001, ^###^ *p* < 0.001, ^##^ *p* < 0.01, ^#^ *p* < 0.05 versus the control group, ^****^ *p* < 0.0001, ^***^ *p* < 0.001, ^**^ *p* < 0.01, ^*^ *p* < 0.05 versus the CORT group.

The exploratory and motor abilities of mice from each group were evaluated using OFT. Our results showed the motion trajectory plots of different experimental groups (Figure 1A). Mice treated with CORT exhibited significantly reduced locomotor activity compared to those in the control group. Administration of Sal (40 mg/kg) ameliorated the depression-like behaviors induced by CORT, increased total distance [F (4, 45) = 17.73, *p* < 0.01], elevated distance [F (4, 45) = 30.05, *p* < 0.01] and time [F (4, 45) = 7.188, *p* < 0.01] in the central zone, as well as increased average velocity [F (4, 45) = 27.58, *p* < 0.05] (Figure 1B). These data demonstrated that Sal can reverse depression-like behaviors in CORT mice.

Repeated administration of CORT significantly reduced the sucrose preference ratio in SPT compared with that in the control group (F (4, 45) = 7.636, *p* < 0.05) (Figure 1C). In contrast, treatment with Sal and FLU significantly increased the sucrose consumption. In addition, FST results showed that compared with the blank group, treatment with CORT obviously elevated the immobility time of mice [F (4, 20) = 7.565, *p* = 0.0003] (Figure 1D **)**. However, there was a significant decline of the immobility time in FST in the groups exposed to FLU or different doses of Sal.

#### Sal Treatment Prevented CORT-Induced Hippocampal Histopathological Damage

The HE-stained sections of the mouse hippocampus in the control group demonstrated that pyramidal cells of the CA1 region were uniform in size and neatly arranged, and many cells had intact structures. In contrast, analysis of the CORT group revealed that the number of nerve cells were significantly decreased, lacked regularity and nuclear pyknosis. These results suggested that the degrees of pathological damage in the hippocampal regions were caused by CORT. Interestingly, the hippocampal changes significantly reversed after Sal treatment [CA1: F (4, 10) = 8.891, *p* < 0.05; CA3: F (4, 10) = 14.98, *p* < 0.05; DG: F (4, 10) = 17.01, *p* < 0.05] (Figure 1E).

#### Sal Improves CORT-Induced BDNF Expression in the Hippocampus

Stress reduces neurogenesis and impairs synaptic plasticity, which are attenuated by antidepressant treatment (Park 2019). BDNF is a neurotrophic factor widely dispersed in the central nervous system, whose increase is critical in mediating the antidepressant effect (Caviedes et al., 2017). We analyzed the effect of Sal on the expression of BDNF in the hippocampus. Our ELISA assay showed that exposure to CORT remarkably decreased BDNF levels in the hippocampus (Figure 1F). Administration of Sal (40 mg/kg) and FLU significantly improved BDNF protein levels in the hippocampus [F (4, 20) = 15.74, *p* < 0.05]. In addition, our findings showed that repeated CORT injections resulted in decreased BDNF protein levels in the hippocampus [F (4, 10) = 8.614, *p* < 0.01] (Figure 1G). In contrast, Sal and FLU upregulated the BDNF expression in depressed mice. Besides, immunohistochemical results demonstrated marked upregulation of BDNF in the group treated with Sal and FLU markedly increased [CA1: (F (4, 10) = 17.50, *p* < 0.05], CA3: [F (4, 10) = 27.17, *p* < 0.05], DG: [F (4, 10) = 12.33, *p* < 0.05)] (Figure 1H). Together, the data demonstrated that Sal can improve CORT-induced depressive symptoms.

#### Sal Mitigates Pyroptosis in CORT-Induced Depression in Mice

**FIGURE 2:**
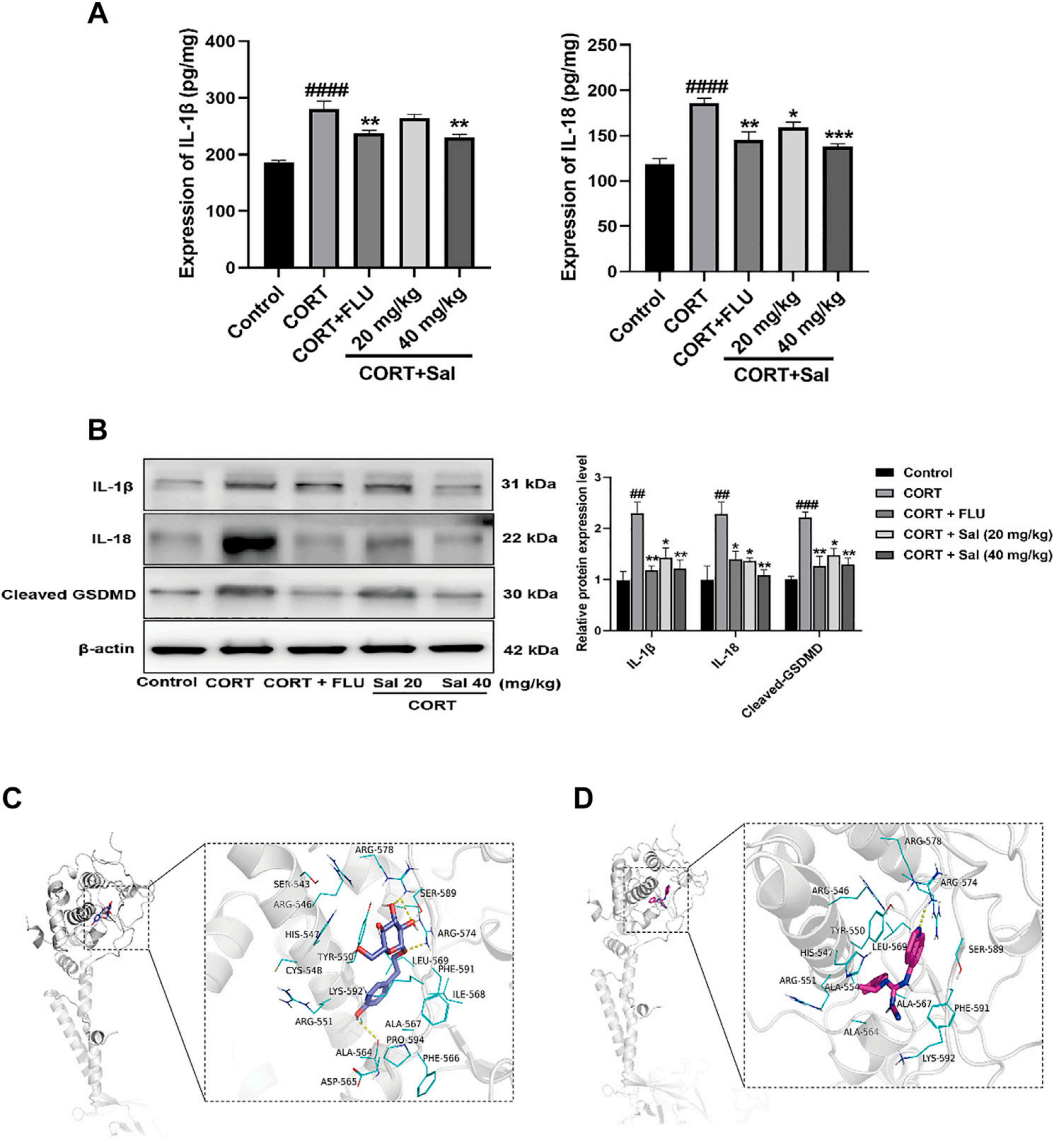
Sal mitigates pyroptosis in CORT-induced depression in mice. **(A)** The levels of IL-1β and IL-18 in the hippocampus were detected by ELISA assay (*n* = 5). **(B)** The measurement of cleaved GSDMD, IL-1β and IL-18 in the hippocampus was detected by Western blot (*n* = 3). **(C)** Structural interactions of Sal with P2X7. 3D diagram of interaction between Sal and P2X7 showed the major binding sites and bonding forces. **(D)** Structural interactions of A-804598 (a P2X7R antagonist) with P2X7. 3D diagram of interaction between P2X7 and A-804598 showed the major binding sites and bonding forces. The Structural overview (left) and the close view (right). Dotted lines (yellow) represent hydrogen bonding interactions. ^####^ *p* < 0.0001, ^##^ *p* < 0.01 versus the control group; ^***^ *p* < 0.001, ^**^ *p* < 0.01, ^*^ *p* < 0.05 versus the CORT group.

We next evaluated the effect of Sal on pyroptosis-associated proteins using Western blot and ELISA. Our ELISA results showed significantly increased expression of IL-18 [F (4, 20) = 16.53, *p* < 0.0001] and IL-1β [F (4, 20) = 21.89, *p* < 0.0001] in the hippocampus of the CORT group compared to the control mice (Figure 2A). Treatment with Sal (20 mg/kg, 40 mg/kg) markedly reversed the protein expression changes. GSDMD is a newly discovered protein, which triggers pyroptosis and promotes the secretion of IL-18 and IL-1β (Li et al., 2019). In the Western blot analysis (Figure 2B), the CORT-stimulated mice showed remarkable upregulation of IL-18 [F (4, 10) = 7.872, *p* < 0.01], IL-1β [F (4, 10) = 8.986, *p* < 0.01] and cleaved GSDMD [F (4, 10) = 12.83, *p* < 0.001], which were all reversed by 40 mg/kg Sal and FLU. Collectively, our data illustrated that CORT triggers pyroptosis, which can be relieved by Sal.

#### Docking Interaction With Sal

As shown in the Figure 2C, Sal binds to a relatively more spacious position at the GDP site of P2X7, and the amino acids interacting with it are SER543, ARG546, HIS547, TYR550, CYS548, LYS592, ARG551, ALA564, ASP565, ALA567, PHE566, ILE568, ARG578, SER589, ARG574, LEU569, PHE591, ILE568, and PRO594. Binding interactions of Sal and P2X7 protein target exhibited hydrogen-bonding interactions at ALA564, ARG574, ARG578 and SER589. Hydrophobic interactions at TYR550, CYS548, ALA564, ALA567, PHE566, ILE568, LEU569, PHE591, and ILE568. In addition, the binding energy of Sal and P2X7 protein receptor is −7.3 Kcal/mol. A-804598, a highly selective P2X7R antagonist, was shown effectively to bind with the P2X7 receptor at the binding site of the drug-binding pocket (Karasawa and Kawate, 2016). We also found that A-804598 could bind with P2X7R at ARG-574 site via hydrogen-bonding interactions. Hydrophobic interactions at TYR-550, LEU569, ALA-554, ALA-564, ALA-567 sites (Figure 2D). The molecular docking results showed that the binding affinity of A-804598 to P2X7 protein receptor is −7.4 Kcal/mol. It could be speculated that binding affinity of Sal with P2X7R is similar to that of A-804598 and P2X7R. Our data suggested that Sal could bind to P2X7 of P2X7/NF-κB/NLRP3 signal pathway.

#### Sal Suppresses P2X7/NF-κB/NLRP3 Signaling Pathway in CORT-Induced Mice

**FIGURE 3:**
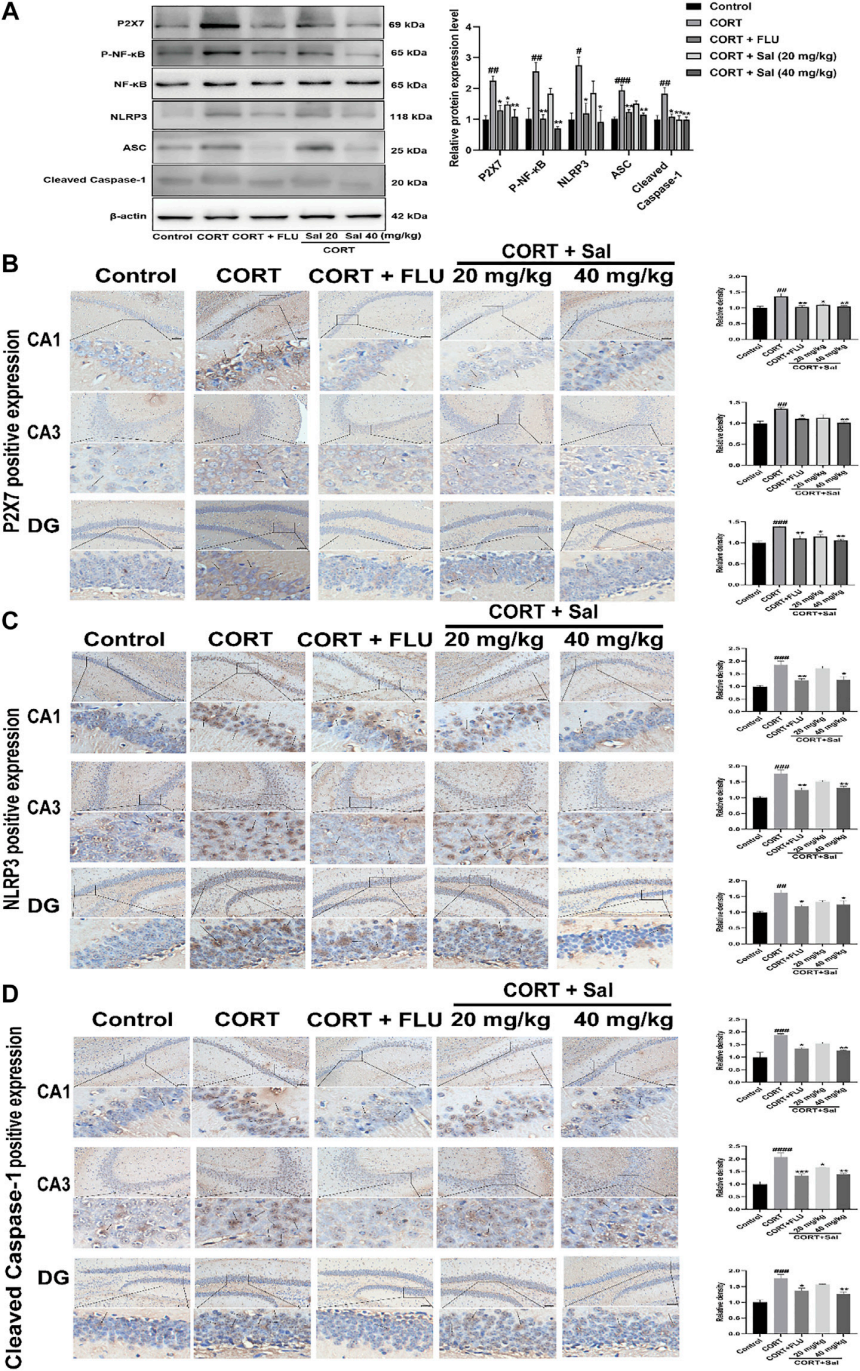
Sal suppresses P2X7/NF-κB/NLRP3 signaling pathway in CORT-induced mice. **(A)** The expression of P2X7, P-NF-κB, and NLRP3 inflammasome-associated proteins in the hippocampus was checked by Western blot (*n* = 3). **(B)** The typical expression of P2X7 in the hippocampus was detected by immunohistochemical staining. **(C)** Sal reduced NLRP3 protein concentrations in the hippocampus was examined by immunohistochemical staining. **(D)** Sal decreased Cleaved caspase-1 expression in hippocampus CA1, CA3, and DG, measured by IHC assay. The typical positive staining of P2X7, NLRP3, and Cleaved caspase-1 were represented by brown staining (black arrows). Original magnification: ×200, *n* = 3. ^####^ *p* < 0.0001, ^###^ *p* < 0.001, ^##^ *p* < 0.01 compared with the control group; ^**^ *p* < 0.01, ^*^ *p* < 0.05 compared with the CORT group.

We performed Western blot and immunohistochemical analyses to study the effect of Sal on the P2X7/NF-κB/NLRP3 signaling pathway in CORT-induced mice. Western blot assay showed increased expression of P2X7 [F (4, 10) = 9.862, *p* < 0.01], P-NF-κB [F (4, 10) = 10.75, *p* < 0.01], NLRP3 [F (4, 10) = 6.814, *p* < 0.01], ASC [F (4, 10) = 14.79, *p* < 0.001], Cleaved caspase-1 [F (4, 10) = 7.596, *p* < 0.01] in the CORT treated mice. However, Sal and FLU notably down-regulated the expression of the P2X7/NF-κB/NLRP3 signaling pathway-related proteins (Figure 3A). Besides, the increased cleaved of GSDMD and elevated caspase-1 activity in the CORT group were obviously blocked by Sal treatment (Figure 2B). These results indicated that Sal mitigated pyroptosis by inhibiting the P2X7/NF-κB/NLRP3 signaling pathway. Similarly, the immunohistochemical analyses showed that administration of CORT dramatically improved the production of P2X7 (CA1: (F (4, 10) = 8.321, *p* < 0.01), CA3: (F (4, 10) = 8.742, *p* < 0.01), DG: (F (4, 10) = 11.22, *p* < 0.001)), NLRP3 (CA1: (F (4, 10) = 13.23, *p* < 0.001), CA3: (F (4, 10) = 15.61, *p* < 0.001), DG: (F (4, 10) = 9.262, *p* < 0.01)) and cleaved Caspase-1 [CA1: (F (4, 10) = 12.82, *p* < 0.001), CA3: (F (4, 10) = 21.48, *p* < 0.001), DG: (F (4, 10) = 15.00, *p* < 0.001)], changes that were reversed by Sal and FLU (Figures 3B–D). Overall, our results demonstrated the correlation between pyroptosis and the P2X7/NF-κB/NLRP3 signaling pathway, and that Sal exerts its effect by suppressing the expression of proteins related with the signaling pathway.

### Sal Ameliorates LPS-Induced Depression

#### Sal Improves LPS-Induced Depression-Like Behaviors

LPS can cause inflammation and is widely used to induce depression-like behaviors (O'Connor et al., 2009; Sekio and Seki, 2014). In this study, LPS-induced acute mouse model was used to explore the ameliorative effects of Sal.

**FIGURE 4:**
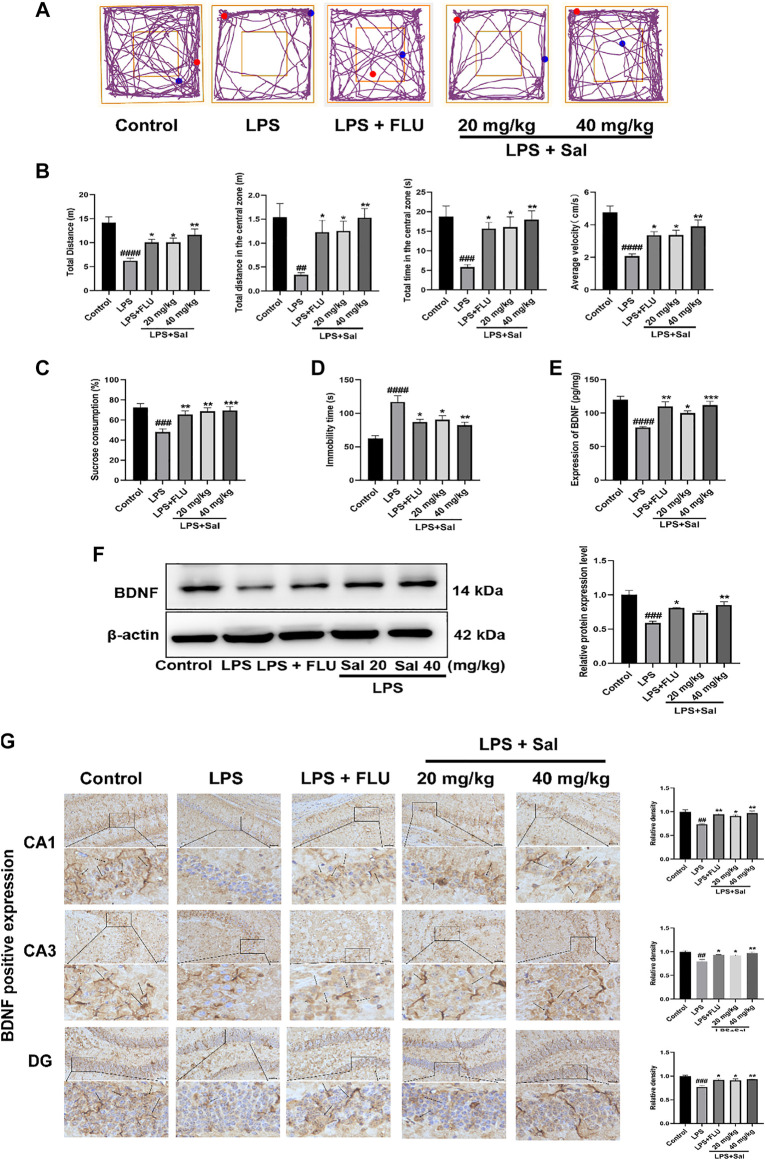
Sal improves LPS-induced depression in mice. **(A)** Representative tracks of different experimental groups in the OFT. **(B)** Sal ameliorated locomotor activity in OFT, including the distance, time and average velocity during the test (*n* = 10). **(C)** Sal prevented the decrease of the consumption of sucrose solution in the SPT (*n* = 10). **(D)** Immobility duration in the FST (*n* = 5). **(E)** Sal improved LPS-induced the decrease of BDNF in the hippocampus was measured by ELISA (*n* = 5). **(F)** Sal improved LPS-induced the decrease of BDNF in the hippocampus was assayed by Western blot (*n* = 3). **(G)** The expression of BDNF in the hippocampus was detected by Immunochemical staining. Brown staining represents the expression of BDNF (black arrows). Original magnification: × 200, *n* = 3. ^####^ *p* < 0.0001, ^###^ *p* < 0.001, ^##^ *p* < 0.01 contrast with the control group; ^***^ *p* < 0.001, ^**^ *p* < 0.01, ^*^ *p* < 0.05 contrast with the LPS group.

As shown in Figure 4A, LPS diminished the autonomous and exploratory behavior of mice in OFT, which were reversed by Sal and FLU. In our study, we showed that the distance traveled in the center [F (4, 45) = 5.472, *p* < 0.01], the time spent in the central zone [F (4, 45) = 6.046, *p* < 0.001], average velocity [F (4, 45) = 10.11, *p* < 0.0001], as well as the total distance [F (4, 45) = 10.00, *p* < 0.0001] were markedly decreased in mice administered with LPS than those in the normal group (Figure 4B).

In addition, the LPS-treated mice showed significantly decreased SPT [F (4, 45) = 7.419, *p* < 0.001] and extended inactivity time of FST [F (4, 20) = 10.71, *p* < 0.0001] in contrast with the control group (Figures 4C,D), phenomena that were restored by Sal and FLU. Thus, Sal prevented LPS-induced depressive-like behaviors.

#### Sal Alleviates LPS-Induced BDNF Expression in the Hippocampus

The effect of Sal on BDNF expression in the LPS-induced mice was assessed by ELISA, Western blot and immunohistochemical analyses. The ELISA data showed that LPS treatment significantly suppressed the BDNF expression compared with that in the control group (Figure 4E). Conversely, pro-administration of Sal and FLU notably increased the expression of BDNF [F (4, 20) = 10.66, *p* < 0.01]. Furthermore, Western blot analyses demonstrated that LPS decreased hippocampus BDNF levels, while Sal treatment remarkably enhanced the production of hippocampus BDNF [F (4, 10) = 15.97, *p* < 0.01] (Figure 4F). Besides, immunohistochemical results showed that Sal reversed the LPS-induced suppression of BDNF in the hippocampus [CA1: (F (4, 10) = 9.960, *p* < 0.05), CA3: (F (4, 10) = 9.655, *p* < 0.05), DG: (F (4, 10) = 10.97, *p* < 0.05)] (Figure 4G). These results indicated that Sal could improve LPS-induced suppression of BDNF in the hippocampus.

#### Sal Relieves Pyroptosis in LPS-Induced Depression in Mice

**FIGURE 5:**
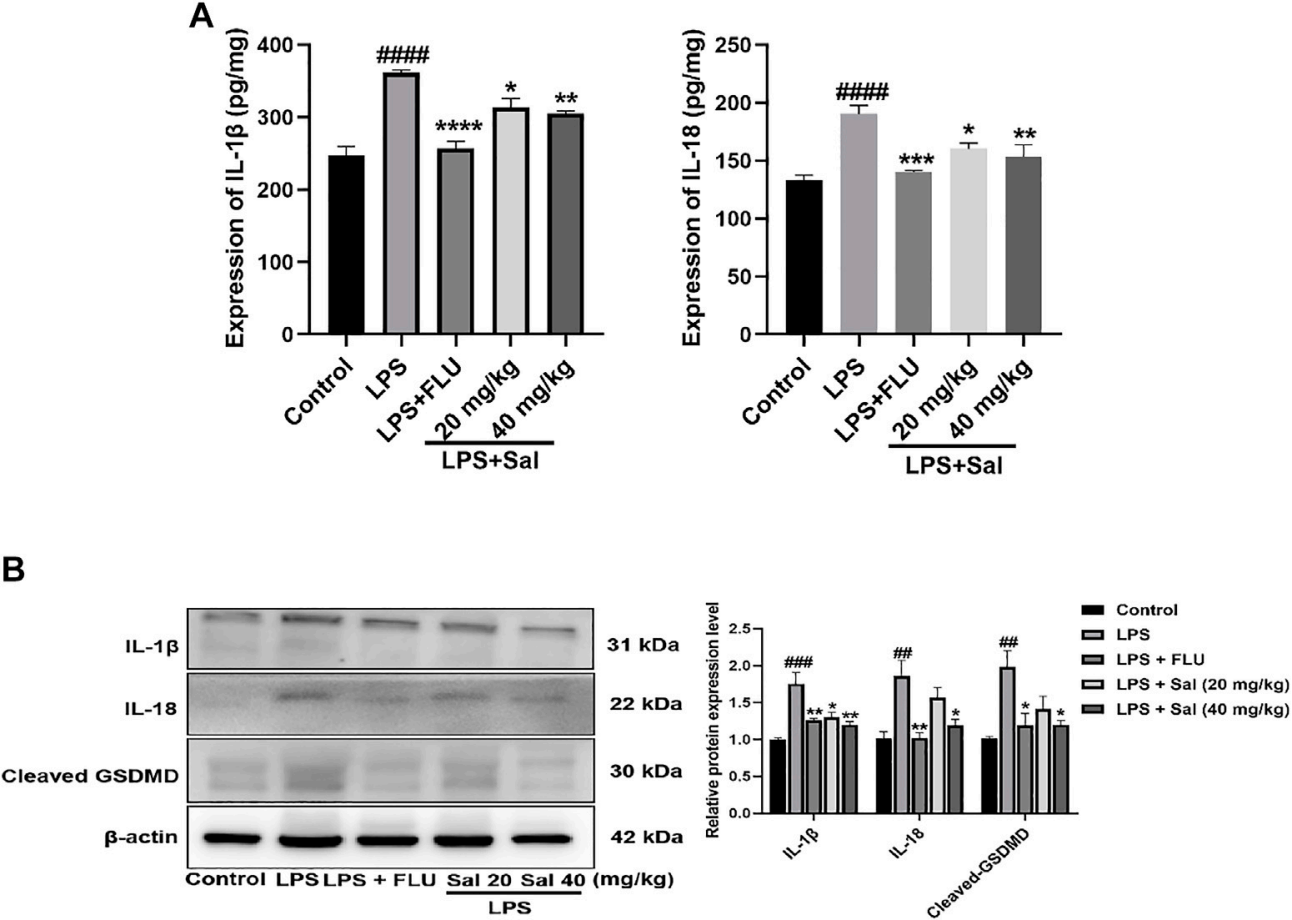
Sal relieves pyroptosis in LPS-induced depression in mice. **(A)** The expression of IL-1β and IL-18 in the hippocampus was measured by ELISA assay (*n* = 5). **(B)** The concentrations of cleaved GSDMD, IL-1β and IL-18 proteins in the hippocampus were analyzed by Western blot (*n* = 3). ^####^ *p* < 0.0001, ^###^ *p* < 0.001, ^##^ *p* < 0.01 compared with the normal group; ^***^ *p* < 0.001, ^**^ *p* < 0.01, ^*^ *p* < 0.05 compared with the LPS group.

The effect of Sal on pyroptosis-related cytokines, including IL-1β and IL-18, was evaluated using ELISA. The data showed that treatment with LPS dramatically increased the expression of IL-1β and IL-18. On the other hand, Sal inhibited the expression of IL-1β [F (4, 20) = 26.07, *p* < 0.05] and IL-18 [F (4, 20) = 12.38, *p* < 0.05] (Figure 5A). Similarly, Western blot analysis showed that Sal could inhibit the production of IL-1β [F (4, 10) = 12.35, *p* < 0.05], IL-18 [F (4, 10) = 8.367, *p* < 0.05], and the cleaved GSDMD [F (4, 10) = 6.542, *p* < 0.05] induced by LPS (Figure 5B). Our data demonstrated the inhibitory effects of Sal which relieve pyroptosis in LPS-induced mice.

#### Salidroside Inhibits P2X7/NF-κB/NLRP3 Signaling Pathway in Lipopolysaccharide-Induced Mice

**FIGURE 6:**
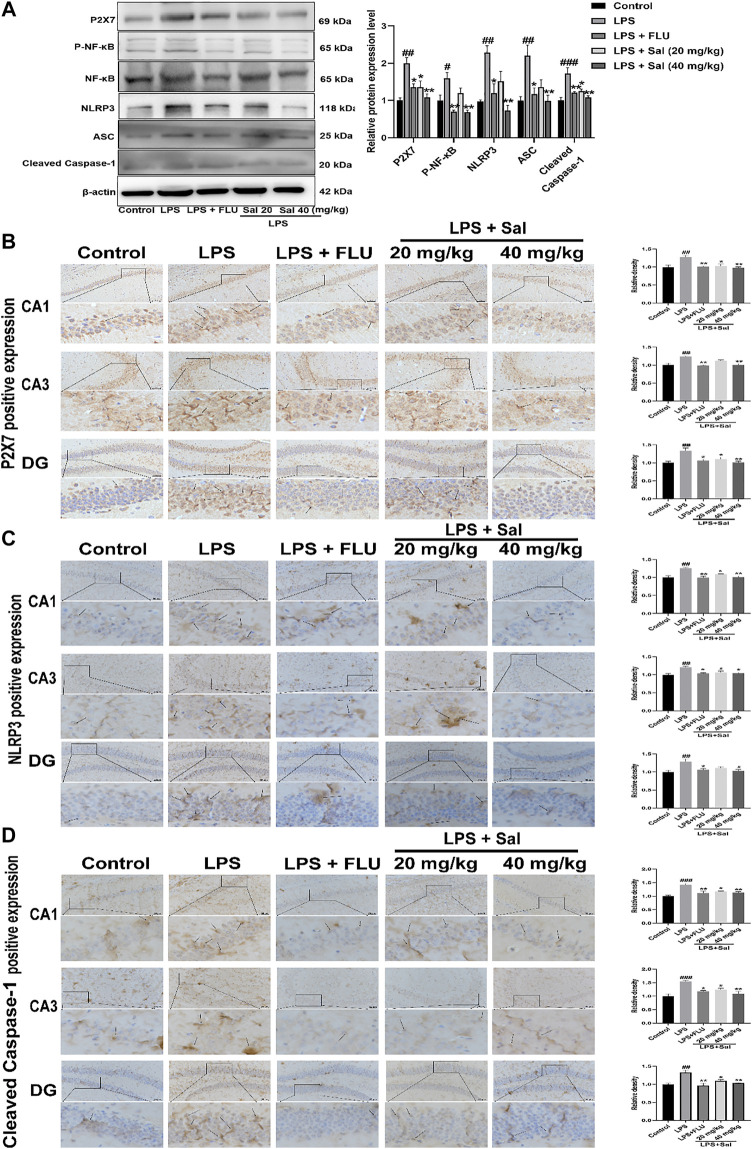
Sal inhibits the P2X7/NF-κB/NLRP3 signaling pathway in LPS-induced mice. **(A)** The levels of proteins, including P2X7, P-NF-κB, NLRP3, ASC, Cleaved caspase-1 in the hippocampus, were determined by Western blot (*n* = 3). **(B)** The levels of P2X7 in the hippocampus were detected by IHC staining. **(C)** Immunohistochemical staining analysis of NLRP3 in the hippocampus. **(D)** Immunohistochemical results of Cleaved caspase-1 in the hippocampus. The typical positive staining of P2X7, NLRP3 and Cleaved caspase-1 were represented by brown staining (black arrows). Original magnification: × 200, *n* = 3. ^###^ *p* < 0.001, ^##^ *p* < 0.01 compared to the control group; ^**^ *p* < 0.01, ^*^ *p* < 0.05 compared to the LPS group.

Mechanisms of the effects of Sal on pyroptosis in LPS-induced mice remain undefined. Western blot and immunohistochemical analyses of LPS-injected mice demonstrated increased expression of proteins such as P2X7 [F (4, 10) = 10.46, *p* < 0.01], P-NF-κB [F (4, 10) = 10.23, *p* < 0.05], NLRP3 [F (4, 10) = 9.431, *p* < 0.01], ASC [F (4, 10) = 6.874, *p* < 0.01], and Cleaved caspase-1 [F (4, 10) = 11.55, *p* < 0.001] in the LPS group. Sal markedly decreased the expression of proteins in the P2X7/NF-κB/NLRP3 signaling pathway (Figure 6A). In addition, the data showed that the P2X7 [CA1: (F (4, 10) = 8.530, *p* < 0.05); CA3: (F (4, 10) = 11.53, *p* < 0.01); DG: (F (4, 10) = 8.022, *p* < 0.05)], NLRP3 (CA1: (F (4, 10) = 10.96, *p* < 0.05); CA3: [F (4, 10) = 7.329, *p* < 0.05); DG: (F (4, 10) = 6.052, *p* < 0.05)] and Cleaved caspase-1 [CA1: (F (4, 10) = 11.56, *p* < 0.05); CA3: (F (4, 10) = 10.89, *p* < 0.05); DG: (F (4, 10) = 10.25, *p* < 0.05)] were notably up-regulated in the mice exposed to LPS. Conversely, administration of Sal and FLU effectively rescued the LPS-induced changes in the P2X7/NF-κB/NLRP3 cascade (Figures 6B–D).

### Sal Restores CORT-Induced Changes in PC12 Cells

#### Sal Ameliorates CORT-Induced Cytotoxicity in PC12 Cells

We used PC12 cells, a cell line from pheochromocytoma of rat adrenal medulla. The cell line has typical neuron characteristics, high expression of glucocorticoid receptors and is widely used in neuropharmacology research (Li M. et al., 2016). Furthermore, a high concentration of corticosterone induces impairment in the PC12 cells, which has been widely used as an *in vitro* model to screen the underlying mechanisms of antidepressants (Zheng et al., 2011). In this study, we used PC12 cells exposed to 200 μM corticosterone to study the neuroprotective effect and potential mechanisms of Sal.

**FIGURE 7:**
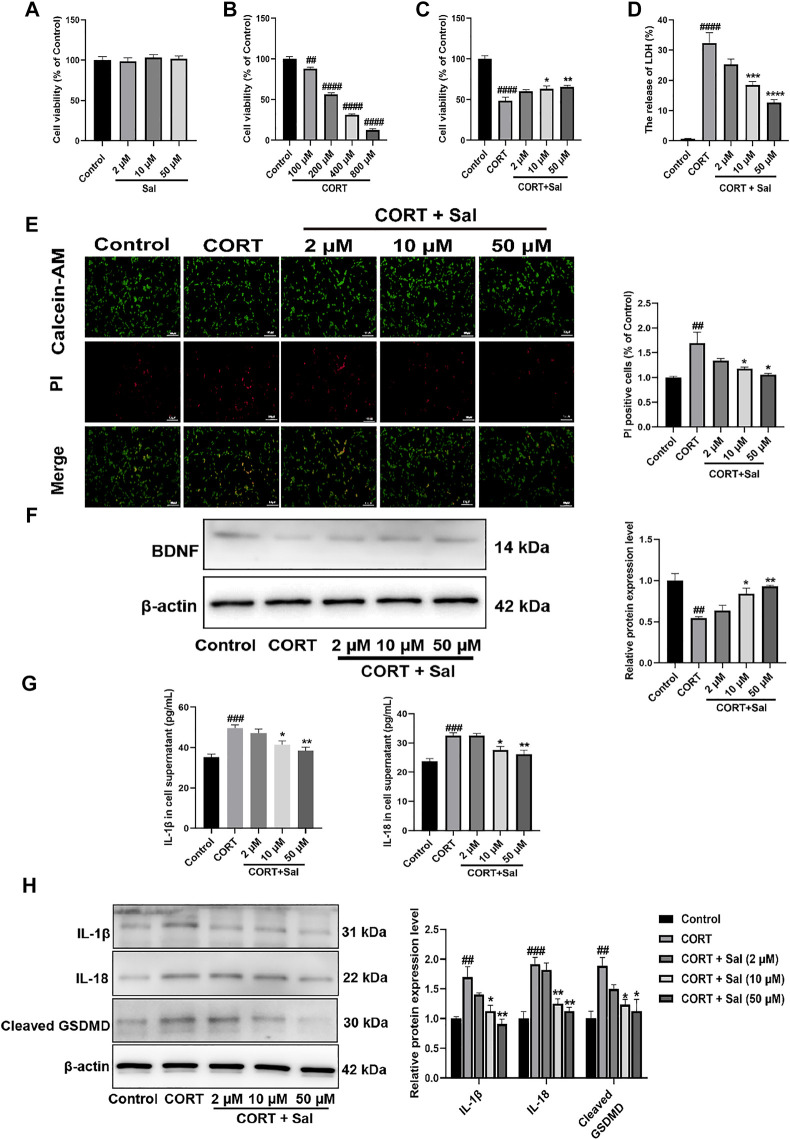
Sal ameliorates CORT-induced PC12 cells. **(A–C)** The cell viability was measured by CCK-8 assay (*n* = 6). **(D)** The release of LDH was determined by LDH assay (*n* = 6). **(E)** Calcein-AM/PI staining images were captured by fluorescence microscopy, Calcein-AM (in green), PI (in red). Original magnification: ×200, Scale bar = 100 μm (*n* = 3). **(F)** Western blot analysis shown that Sal increased BDNF expression in CORT-induced PC12 cells (*n* = 3). **(G)** The expression of IL-1β and IL-18 in PC12 cell supernatant was detected by ELISA (*n* = 5). **(H)** The levels of IL-1β, IL-18 and cleaved GSDMD in supernatant of PC12 cell were analyzed by Western blot (*n* = 3). [**(A)** PC12 cells were cultured with various concentrations of Sal (2, 10, 50 μM) for 24 h **(B)** PC12 cells were treated with increasing doses of CORT (100–800 μM) for 24 h **(C–H)** PC12 cells were incubated with Sal (2, 10, 50 μM) for 2 h, and then cultured with 200 μM CORT for 24 h] ^####^ *p* < 0.0001, ^###^ *p* < 0.001, ^##^ *p* < 0.01 compared to the control group; ^**^ *p* < 0.01, ^*^ *p* < 0.05 compared to the CORT group.

To explore the effect of Sal on cell viability, different concentrations of Sal were used in CCK-8 assay (Figure 7A **)**. We selected the optimal concentration of corticosterone, which acts on the PC12 cells as assessed by the CCK-8 assay. The data showed that 100–800 μM CORT gradually decreased the cell viability with increase in concentration [F (4, 25) = 351.0, *p* < 0.01] (Figure 7B **)**. 200 μM CORT reduced the cell viability to 56% and was then chosen in subsequent experiments. Compared with the model group, the cell survival rates of 10 μM and 50 μM Sal were 65% and 70%, respectively, which were significantly increased [F (4, 25) = 30.62, *p* < 0.05] (Figure 7C). 2 μM Sal also improved the cell viability, with no significant difference. In addition, our results showed that Sal treatment suppressed the release of LDH in the cell supernatant compared with the CORT group [F (4, 25) = 43.08, *p* < 0.001] (Figure 7D). Calcein-AM/PI staining data showed that CORT stimulation enhanced the number of PI-positive cells, which was remarkably suppressed following treatment with Sal (F (4, 10) = 6.902, *p* < 0.05) (Figure 7E). Further analyses revealed that CORT (200 μM) decreased the expression of BDNF in the PC12 cells, which is sync with the *in vivo* findings, while pretreatment with Sal remarkably restored the BDNF levels [F (4, 10) = 11.69, *p* < 0.05] (Figure 7F). These findings suggested that Sal could protect the PC12 cells against CORT-induced cytotoxicity.

#### Sal Suppresses CORT-Induced Pyroptosis in PC12 Cells

GSDMD-dependent membrane pore formation is an ultimate event of pyroptosis, which promotes the release of pro-inflammatory mediators, such as IL-18 and IL-1β (Zhang et al., 2020). We employed ELISA to analyze the levels of IL-1β [F (4, 20) = 11.45, *p* < 0.001] and IL-18 [F (4, 20) = 12.64, *p* < 0.001] in the PC12 cells as shown in Figure 7G **.** On the other hand, the expression of pyroptosis-related proteins was measured by Western blot assays (Figure 7H). In sync with the *in vivo* findings, the IL-1β [F (4, 10) = 10.92, *p* < 0.01], IL-18 [F (4, 10) = 17.93, *p* < 0.001] and cleaved GSDMD [F (4, 10) = 7.424, *p* < 0.01] proteins were upgraded in the PC12 cells, which were all significantly reversed by Sal. The cellular membrane integrality was evaluated by Calcein-AM/PI staining and LDH release. These results also indicated that CORT-induced pyroptosis in PC12 cells. Taken together, these findings demonstrated that the protective effects of Sal against CORT-induced cell injury was mediated through inhibiting of pyroptosis.

#### Sal Reverses CORT-Induced Pyroptosis by Restraining P2X7/NF-κB/NLRP3 Signaling Pathway

**FIGURE 8:**
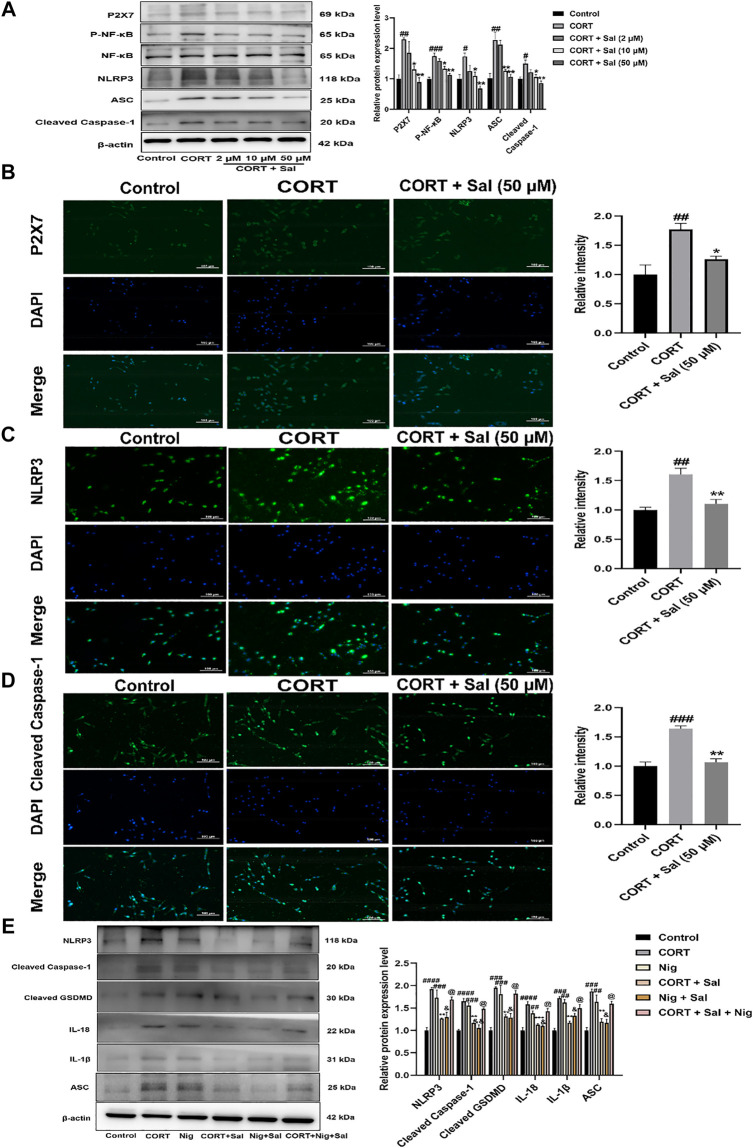
Sal ameliorates pyroptosis by inhibiting P2X7/NF-κB/NLRP3 signaling pathway in CORT-induced PC12 cells. **(A)** The protein expression of P2X7, P-NF-κB, NLRP3, ASC, Cleaved caspase-1 in PC12 cells was detected by Western blot (*n* = 3). PC12 cells were pre-treated with Sal (2, 10, 50 μM) for 2 h, and then stimulation with 200 μM CORT for 24 h. **(B)** Immunofluorescence staining of P2X7 in PC12 cells (*n* = 3). **(C)** Immunofluorescence detection of the expression of Cleaved caspase-1 in PC12 cells (*n* = 3). **(D)** The results of immunofluorescence demonstrated the expression level of NLRP3 in PC12 cells (*n* = 3). PC12 cells were pre-treated with Sal (50 μM) for 2 h, and then stimulation with 200 μM CORT for 24 h. Original magnification: ×200. **(E)** The protein levels of NLRP3, ASC, Cleaved caspase-1, IL-1β, IL-18, and Cleaved GSDMD were detected using Western blot (*n* = 3). ^####^ *p* < 0.0001, ^###^ *p* < 0.001, ^##^ *p* < 0.01 versus the control group; ^***^ *p* < 0.001, ^**^ *p* < 0.01 versus the CORT model group; ^&&^ *p* < 0.01, ^&^ *p* < 0.05 represent Nig + Sal group versus Nig group; ^@^ *p* < 0.05 represent CORT + Sal + Nig group compared with CORT + Sal group.

To further evaluate the potential molecular mechanisms of Sal in pyroptosis, we assayed the expression of proteins associated with the P2X7/NF-κB/NLRP3 signaling pathway in the PC12 cells. Western blot analysis showed that the expression levels of P2X7 [F (4, 10) = 9.217, *p* < 0.01], P- NF-κB [F (4, 10) = 18.23, *p* < 0.001], NLRP3 [F (4, 10) = 8.077, *p* < 0.05], ASC [F (4, 10) = 15.38, *p* < 0.01], and Cleaved caspase-1 [F (4, 10) = 7.748, *p* < 0.05] were significantly higher in the CORT-damaged PC12 cells, and the proteins were obviously down-regulated by Sal (Figure 8A). Our immunofluorescence results demonstrated that Sal-treatment greatly decreased the concentrations of P2X7 [F (2, 6) = 11.73, *p* < 0.05], NLRP3 [F (2, 6) = 16.85, *p* < 0.01] and Cleaved caspase-1 [F (2, 6) = 35.47, *p* < 0.01] in the PC12 cells (Figures 8B–D). These results indicated that Sal relieves pyroptosis in CORT-induced PC12 cells.

Based on the essentiality of NLRP3 in pyroptosis, we assessed the effect of nigericin (an NLRP3 agonist). Our Western blot assay showed that treatment with CORT and nigericin increased the expression of NLRP3 [F (5, 12) = 15.64, *p* < 0.001], ASC [F (5, 12) = 15.19, *p* < 0.01], Cleaved caspase-1 [F (5, 12) = 23.65, *p* < 0.001], IL-1β [F (5, 12) = 15.21, *p* < 0.01], IL-18 [F (5, 12) = 19.42, *p* < 0.01] and Cleaved GSDMD [F (5, 12) = 15.11, *p* < 0.001] *in vitro*. Nonetheless, these effects were all abolished by the Sal (Figure 8E). In addition, compared with the CORT + Sal group, the levels of NLRP3, ASC, Cleaved caspase-1, IL-1β and IL-18 were increased in the CORT + Sal + Nig group (All *p* < 0.05). Nig weakened the inhibitory effect of Sal on pyroptosis. Thus, these results demonstrated that the anti-pyroptosis effect of Sal is associated with NLRP3.

**FIGURE 9:**
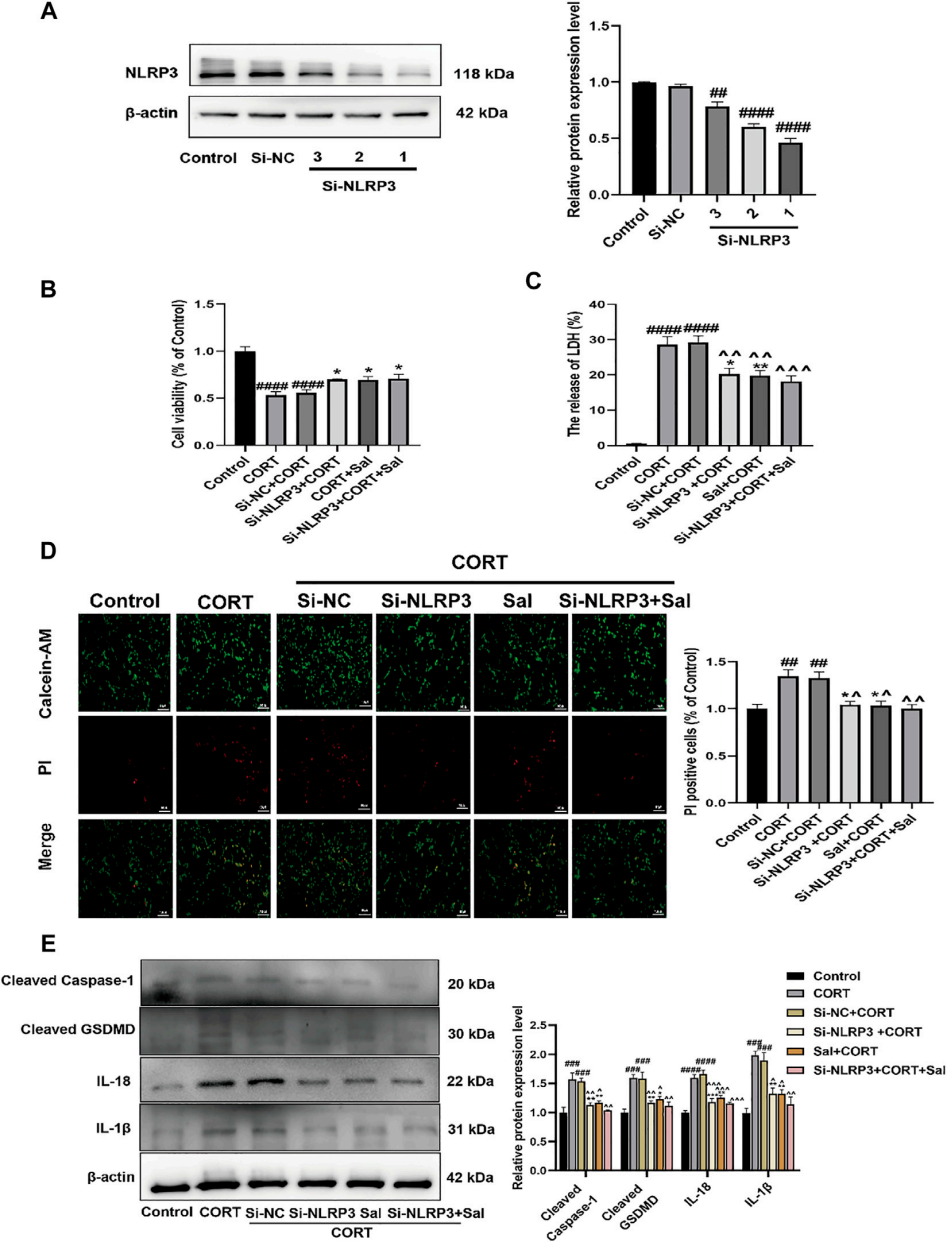
Sal and knockdown of NLRP3 attenuates CORT-induced pyroptosis in PC12 cells. **(A)** NLRP3 was knocked down by NLRP3 siRNA in PC12 cells, transfection efficiency of NLRP3 was detected by western blot (*n* = 3). **(B)** The viability of cells was detected by CCK-8 assay (*n* = 6). **(C)** Cell cytotoxicity in PC12 cells was detected by LDH cytotoxicity assay (*n* = 6). **(D)** Cell viability and cytotoxicity were measured by Calcein-AM/PI staining. Calcein-AM (in green), PI (in red). Original magnification: ×200, Scale bar = 100 μm, *n* = 3. **(E)** The levels of cleaved caspase-1, IL-1β, IL-18, and cleaved GSDMD in PC12 cells were assessed by western blot analysis (*n* = 3). ^####^ *p* < 0.0001, ^###^ *p* < 0.001, ^##^ *p* < 0.01 compared with the control group; ^**^ *p* < 0.01, ^*^ *p* < 0.05 compared with the CORT group; ^^^^^^ *p* < 0.0001, ^^^^^ *p* < 0.001, ^^^^ *p* < 0.01, ^^^ *p* < 0.05 compared with Si-NC + CORT group.

Next, to investigate the impact of NLRP3 on Sal-specific anti-pyroptosis effects, the PC12 cells were transfected with Si-NLRP3. NLRP3 was successfully knocked down by Si-RNA, as demonstrated by Western blot data (Figure 9A). We selected the Si-NLRP3 with the best knockdown effect for subsequent experiments. Our data showed that CORT decreased the viability of the cells, which was ameliorated by Si-NLRP3 [F (5, 30) = 21.02, *p* < 0.05] (Figure 9B). In addition, there was increased release of LDH following the CORT treatment. Furthermore, compared with the CORT group, Si-NLRP3 or Sal treatment significantly downregulated the LDH release [F (5, 30) = 39.81, *p* < 0.05] (Figure 9C). Our results showed increased PI positive stained cells in the CORT-group, effects that were abolished by Sal and Si-NLRP3 [F (5, 12) = 9.565, *p* < 0.05] (Figure 9D). As illustrated in Figure 9E **,** NLRP3 knocked down with SiRNA decreased the expression of pyroptosis-related proteins, including cleaved caspase-1 [F (5, 12) = 14.67, *p* < 0.01], IL-1β [F (5, 12) = 17.39, *p* < 0.01], IL-18 [F (5, 12) = 29.81, *p* < 0.01], and cleaved GSDMD [F (5, 12) = 14.44, *p* < 0.01] in the PC12 cells treated with CORT.

## Discussion

**FIGURE 10:**
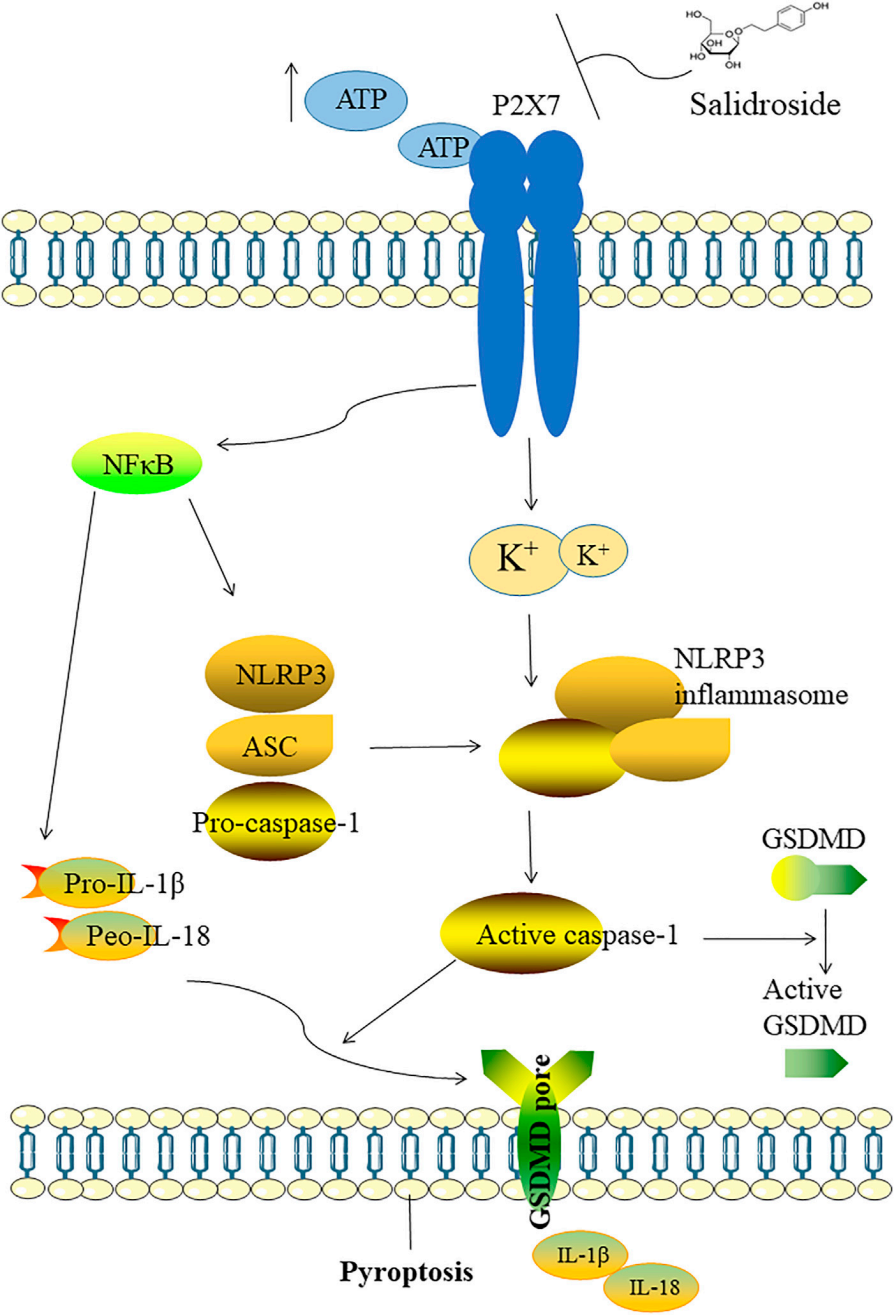
Illustration of the mechanism which Sal improves depression through suppressing NLRP3-mediated pyroptosis. The possible mechanism of Sal is as follows: Sal suppresses NLRP3-mediated pyroptosis through inhibiting P2X7/NF-κB/NLRP3 signaling pathway.

Recent studies have reported that Sal exerts various pharmacological effects such as anti-inflammatory, antioxidant activities (Grech-Baran et al., 2015; Fan et al., 2020). Other studies have demonstrated that Sal can ameliorate cognitive and motor abilities by suppressing the dysfunction of hippocampal neurons (Gao et al., 2015; Palmeri, Mammana, Tropea, Gulisano and Puzzo 2016). In our study, we explored antidepressant-like effects of Sal and its underlying mechanisms. The data showed that Sal improves depression-like behavior in mice, inhibits pyroptosis and suppresses NLRP3-mediated pyroptosis via inhibition of P2X7/NF-κB/NLRP3 signaling pathway, as illustrated in Figure 10.

The pathogenesis of depression is very complex, and the proposed “neurotropism and plasticity” theory is gradually gaining attention in recent years. According to this theory, depression is caused by decreased neurotrophic factors, impaired synaptic plasticity and reduced neural plasticity (Duman et al., 2016). BDNF is involved in the growth and maintenance of neurons and is essential for cognitive function (Phillips 2017). Research has shown that the level of BDNF in the hippocampus and prefrontal cortex is significantly reduced in patients with depression (Taliaz et al., 2010). In this study, we showed that Sal significantly ameliorates CORT or LPS induced suppression of hippocampal BDNF expression. Besides, it has been reported that patients with depression have an overactive hypothalamic-pituitary-adrenal (HPA) axis and increased cortisol levels (Keller et al., 2017). Repeated injections of CORT in animals cause deregulation of the HPA axis, neuronal damage, cognitive and memory decline, and induce depression-like behavior (Zhao et al., 2008; Badr et al., 2020). Thus, CORT-induced model has been widely used as a chronic model of depression caused by stress (Kinlein et al., 2019). In addition, high concentrations of CORT have been used to induce injury in PC12 cells, which emulates pathological mechanisms of depression (Mao et al., 2012). Therefore, CORT-induced depression model is a reliable model for screening antidepressants and exploring their pharmacological mechanisms. Our *in vivo* results showed that CORT administration significantly decreased sucrose preference rate, increased the time of immobility in FST, and reduced motility in OFT. However, Sal administration profoundly improved the behavioral defects in SPT, OFT and FST, implying that Sal alleviates depression-like behavior in mice. Furthermore, our data showed that CORT induced damage in the PC12 cells, release of LDH and increased PI staining, which are reversed by Sal. In a previous study, fluoxetine, a typical selective serotonin reuptake inhibitor (SSRI), was shown to effectively reverse anxiety/depression-like behaviors (Alboni et al., 2017). In agreement, our findings showed that chronic treatment with fluoxetine also significantly increased the mobility time in the FST as well as the time spent in OFT. Thus, treatment with Sal and FLU effectively alleviated the depression-like behavior of mice and FLU was used as a positive control in this experiment.

Neuroinflammation is an essential occurrence in the pathogenesis of psychiatric diseases, which is associated with the pathogenesis of depression Galecki and Talarowska, 2018). Normal levels of inflammatory cytokines play a critical role in signaling, but excessive stress and long-term chronic inflammation can lead to neuroinflammatory, cellular damage, and exacerbation of neurodegenerative diseases as well as psychiatric disorders (Voet et al., 2019). Evidence has shown a strong correlation between depression and peripheral inflammatory markers in blood and cerebrospinal fluid (CSF). For instance, a recent cumulative meta-analysis reported that interleukin-6 (IL-6), IL-1β and C-reactive protein (CRP) are associated with depression (Haapakoski et al., 2015). Under physiological conditions, inflammasome-induced IL-1β expression is essential as a trophic support to memory formation (Yirmiya et al., 2002). However, at high levels, IL-1β becomes excitotoxic, which alters synaptic activity (Ross et al., 2003; Huang et al., 2011). Its expression and cleavage are influenced by NF-κB and NLRP3 (Wen et al., 2012; Zhong et al., 2016). Another study showed that NLRP3 inflammasome regulated the secretion of BDNF (Ward et al., 2019). This inverse correlation between the expression of BDNF and NLRP3 was also observed in the hippocampus of diabetic rats (Ye et al., 2018). It has been demonstrated that the NLRP3 inflammasome mediates depressive-like behaviors in animals (Zhang et al., 2014). Clinical data regarding the involvement of the NLRP3 inflammasome in patients with depression are scarce. A previous report showed that caspase-1, NLRP3 mRNA expression, and NLRP3 protein levels are increased in mononuclear blood cells in patients with depression (Alcocer-Gomez et al., 2014). In our study, mice exposed to CORT showed elevated levels of IL-18, IL-1β, and NLRP3, which were all attenuated by Sal intervention.

The NLRP3 inflammasome, an important component of the immune system, is one of the most intensively studied inflammasomes, and consists of NLRP3, apoptosis speck-like protein and pro-caspase-1. The NLRP3 inflammasome is present in microglia and astrocytes in the CNS (Song et al., 2017). An increase in expression of NLRP1 and NLRP3 inflammasome proteins and IL-1β and IL-18 proteins in neurons under ischemic conditions (Fann et al., 2013). A previous study also implied that the expression of NLRP3 in neurons increased in patients with Rasmussen’s encephalitis (V. et al., 2013). Activation of the NLRP3 inflammasome complex promotes activation of caspase-1, which further induces cleavage of GSDMD to a GSDMD-N terminal structure with pore-forming activity, eventually leading to cell rupture, and further inducing pyroptosis. In parallel, pro-IL-1β and pro-IL-18 are sheared by csapase-1 and can be excreted through the channels formed by the GSDMD-N terminal domain, thus triggering the pyroptosis-associated inflammatory cascade (Shi et al., 2015). Recent studies have shown that stress participates in the pathology of depression by activating the NLRP3 complex, which induces pyroptosis (Arioz 2019). Our *in vivo* and *in vitro* results indicated that Sal treatment significantly decreased pyroptosis-related protein levels including cleaved GSDMD, cleaved caspase-1, IL-1β, and IL-18. Therefore, the protective effect of Sal against depression-like behavior may be associated with the inhibition of pyroptosis. Pyroptosis is often accompanied by inflammation, which performs an essential role in the fight against infections. The activated NLRP3 inflammasome is one of the most important mediators of pyroptosis, which is involved in various inflammatory and immune diseases (Liang et al., 2020). Interestingly, we observed the characteristic hallmarks of pyroptosis in depression, which included increased activation of NLRP3 inflammasome and the release of IL-1β and IL-18. To evaluate whether NLRP3-mediated pyroptosis in depression, nigericin (an NLRP3 agonist) was added to activate NLRP3 in Sal-protected CORT-damaged PC12 cells. The data demonstrated that nigericin (an NLRP3 agonist) could reverse the protective effect of Sal *in vitro*. In addition, knockdown of the NLRP3 with siRNA significantly inhibited cell pyroptosis and improved cell viability. These results demonstrated that NLRP3 silencing and Sal treatment could prevent cell damage and ameliorate pyroptosis in PC12 cells. Our findings implied that NLRP3 may be a potential therapeutic target for depression and Sal can improve depressive symptoms by inhibiting the NLRP3-mediated pyroptosis.

It has been reported that NLRP3 inflammasome is activated by various stimuli, including ATP and K^+^ ionophores (Kelley et al., 2019). The P2X7 receptor, a subgroup of the P2X family distributed on cell membranes, is highly expressed in various neurons and immune cells and is closely associated with neuroinflammation and immunity (Kanellopoulos and Delarasse, 2019). P2X7 receptors are activated by ATP to induce overactivation of the NLRP3 inflammasome. As a key inflammation switch, P2X7 receptor participates in the development of depression by influencing the release of IL-1β (Yue et al., 2017). In our previous study, we have demonstrated that the activation of P2X7/NF-κB signaling pathway is involved in depression (Zhang et al., 2016). NF-κB, a key transcriptional activator of the NLRP3 inflammasome, is important in the activation and assembly of the inflammasome. NF-κB and NLRP3 are interconnected inflammatory pathways, and their interaction promotes the emission of inflammatory factors, leading to pyroptosis (Swanson et al., 2019). In addition, the NF-κB-mediated transcription can regulate the ability of the P2X7 receptor to initiate caspase-1 and fuel secretion of IL-18 (Kahlenberg et al., 2005). In our study, the molecular docking analysis predicted the interaction of Sal with P2X7, a upstream of P2X7/NF-κB/NLRP3. To study the underlying mechanisms of NLRP3-mediated pyroptosis, we analyzed the expression of P2X7R, ASC, and other proteins in hippocampus and cell suspensions by Western blot analysis. The data showed that the expression of proteins related with P2X7/NF-κB/NLRP3 signaling pathway-related and pyroptosis was significantly inhibited after Sal treatment. At the same time, there was improvement in the neurological function and histopathology. Our findings suggested that Sal could significantly alleviate depressive symptoms, and it may be dependent on the P2X7/NF-κB/NLRP3 pathway-mediated pyroptosis.

Previous studies demonstrated that LPS stimulation leads to pyroptosis and secretion of active IL-1β (Russo et al., 2021). In addition, the LPS-induced depression model was based on an inflammation-associated model of acute depression, and lower doses were more likely to trigger symptoms such as inflammatory response, reduced sucrose preference rate, and reduced exploratory activity in the OFT (Arioz 2019; Zheng et al., 2021). To further investigate the anti-inflammatory and antidepressant-like effect mechanisms of Sal, our study used a mice model with acute injection of LPS. The data showed that treatment with Sal led to decreased immobility and increased sucrose preference rate in the LPS-induced mice, and significantly improved exploratory and locomotor abilities in the OFT. In addition, the expression of NLRP3, ASC, Cleaved caspase-1 and pyroptosis-related proteins increased significantly under stimulation by LPS, which were ameliorated by Sal. Taken together, these data suggest that Sal reverses LPS-induced depressive behavior.

## Conclusions

In conclusion, our findings indicated that NLRP3-mediated pyroptosis exerted an essential effect on depressive symptoms. Precisely, Sal can ameliorate pyroptosis via suppressing the P2X7/NF-κB/NLRP3 pathway. Furthermore, we revealed that Sal provides novel therapeutic strategies for the treatment of depression.

## Data Availability

The original contributions presented in the study are included in the article/Supplementary Material, further inquiries can be directed to the corresponding authors.

## Abbreviations

ATP, Adenosine triphosphate; ANOVA, Analysis of variance; BDNF, Brain Derived Neurotrophic Factor; CCK-8, Cell Counting Kit-8; CORT, Corticosterone; Caspase-1, Cysteinyl aspartate specific proteinase; ELISA, Enzyme-linked immunosorbent assay; FLU, fluoxetine; FST, Forced swim test; GSDMD, Gasdermin D; HPA, Hypothalamic-pituitary-adrenal axis; IF, Immunofluorescence assay; IL-1β, Interleukin 1 beta; LPS, Lipopolysaccharide; NF-κB, Nuclear Factor Kappa B; Nig, Nigericin; NLRs, NOD-like receptors; OFT, Open field test; PFA, Paraformaldehyde; PVDF, Polyvinylidene difluoride; Sal, Salidroside; SPT, Sucrose preference test.
