# Supplementary material for: Salidroside Ameliorates Depression by Suppressing NLRP3-Mediated Pyroptosis via P2X7/NF-κB/NLRP3 Signaling Pathway
Source: Front Pharmacol. 2022 Apr 12;13:812362. doi: 10.3389/fphar.2022.812362 (PMC9039222; doi:10.3389/fphar.2022.812362)
Supplement: Supplementary file 1 [file DataSheet1.ZIP › Supplementary Materials/supplementary materials ( original western blot figures)/supplementary materials ( original western blot figures).pdf]

Supplementary materials (original western blot figures):

Legends of the figure

FIGURE 1 | Effects of Sal in CORT-induced depression in mice.

The original western blot bands of BDNF, β-actin protein in CORT-induced depressive mice.

The lane order from left to right on blot is: (1) the control group, (2) the CORT group, (3) the CORT + FLU group, (4) the CORT + Sal (20 mg/kg) group, (5) the CORT + Sal (40 mg/kg) group.

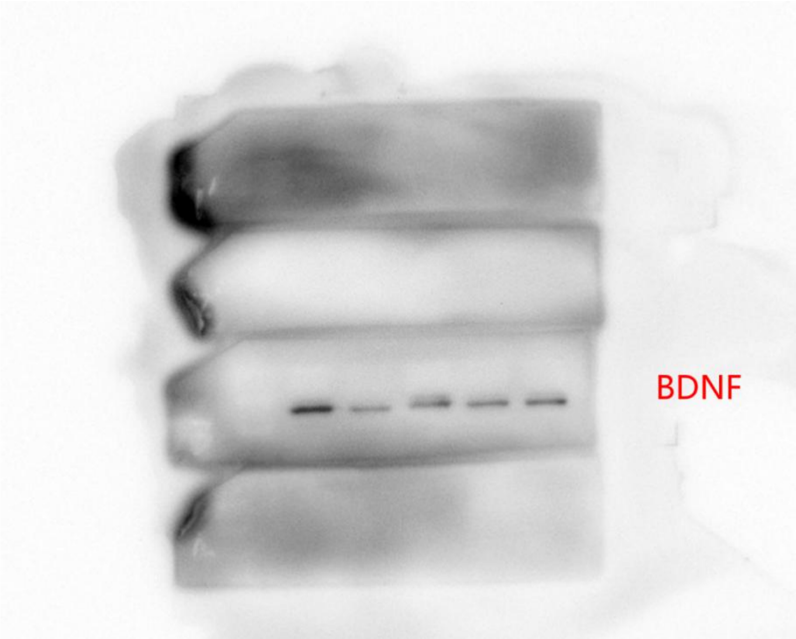

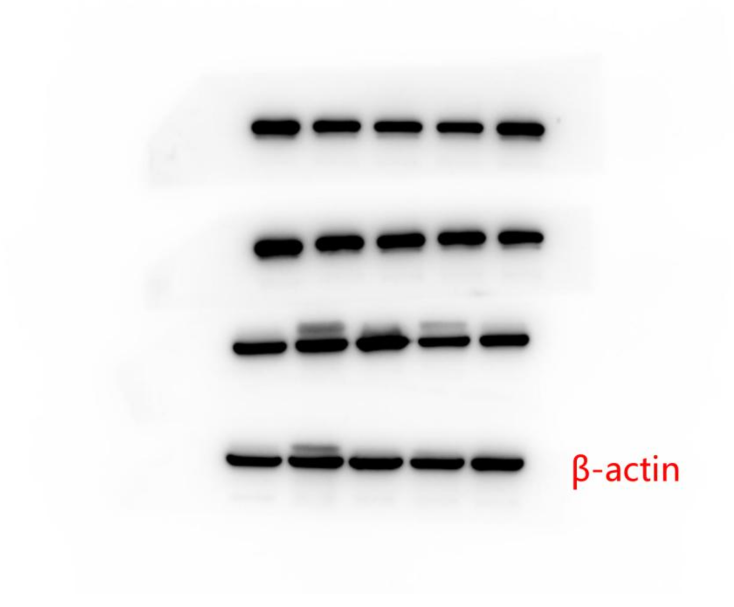

FIGURE 2 | Sal ameliorated pyroptosis in CORT-induced depression in mice.

The original western blot bands of IL-1β, IL-18, cleaved GSDMD and β-actin protein in CORT-induced depressive mice.

The lane order from left to right on blot is: (1) the control group, (2) the CORT group, (3) the CORT + FLU group, (4) the CORT + Sal (20 mg/kg) group, (5) the CORT + Sal (40 mg/kg) group.

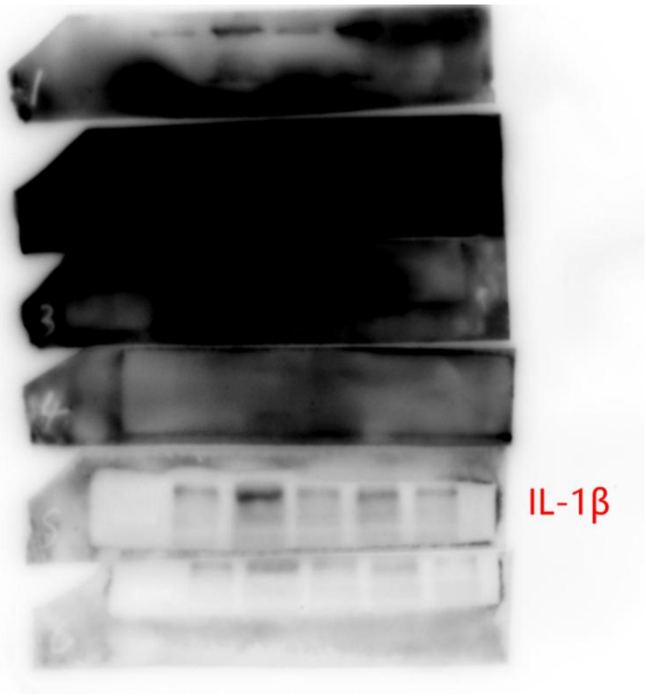

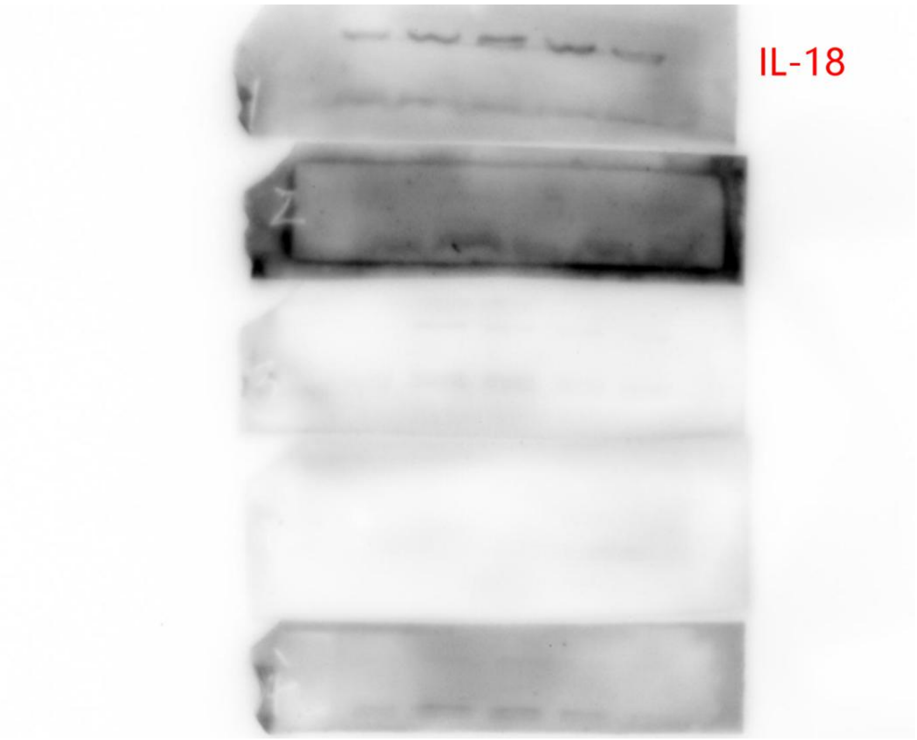

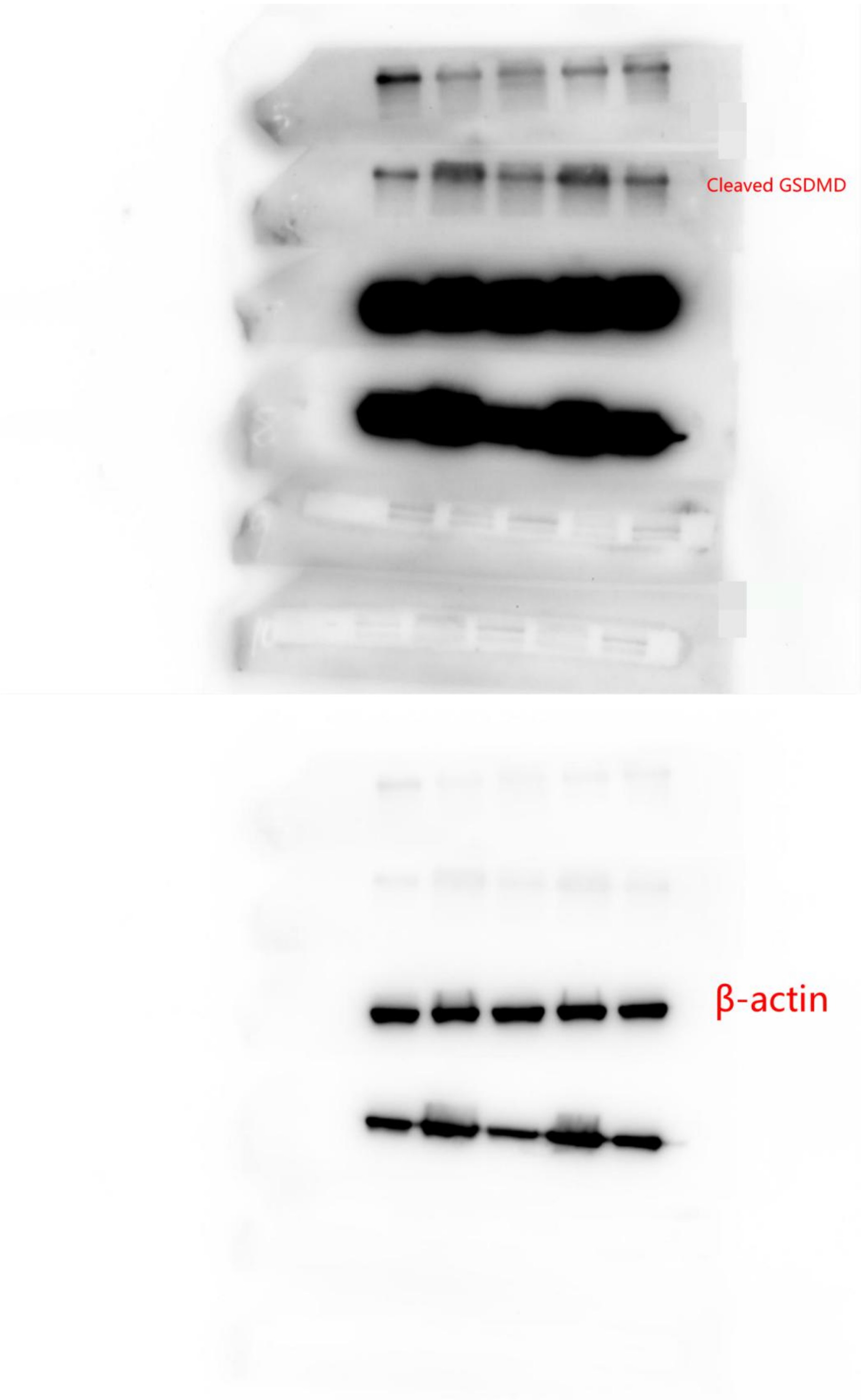

FIGURE 3 | Sal relieved pyroptosis via the P2X7/NF-κB/NLRP3 signaling pathway in CORT-induced depression in mice.

The original western blot bands of P2X7, P-NF-κB, NF-κB, NLRP3, ASC, Cleaved caspase-1 and β-actin protein in CORT-induced depressive mice.

The lane order from left to right on blot is: (1) the control group, (2) the CORT group, (3) the CORT + FLU group , (4) the CORT + Sal (20 mg/kg) group, (5) the CORT +

Sal (40 mg/kg) group.

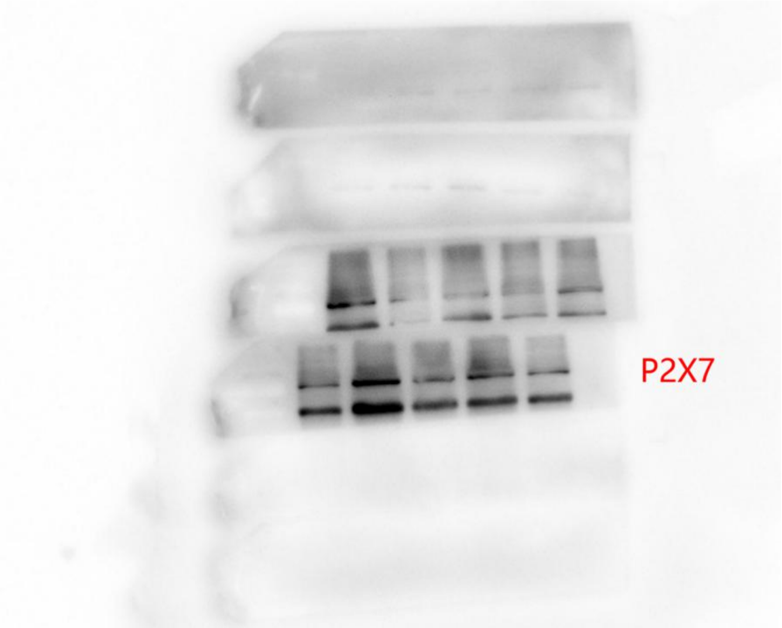

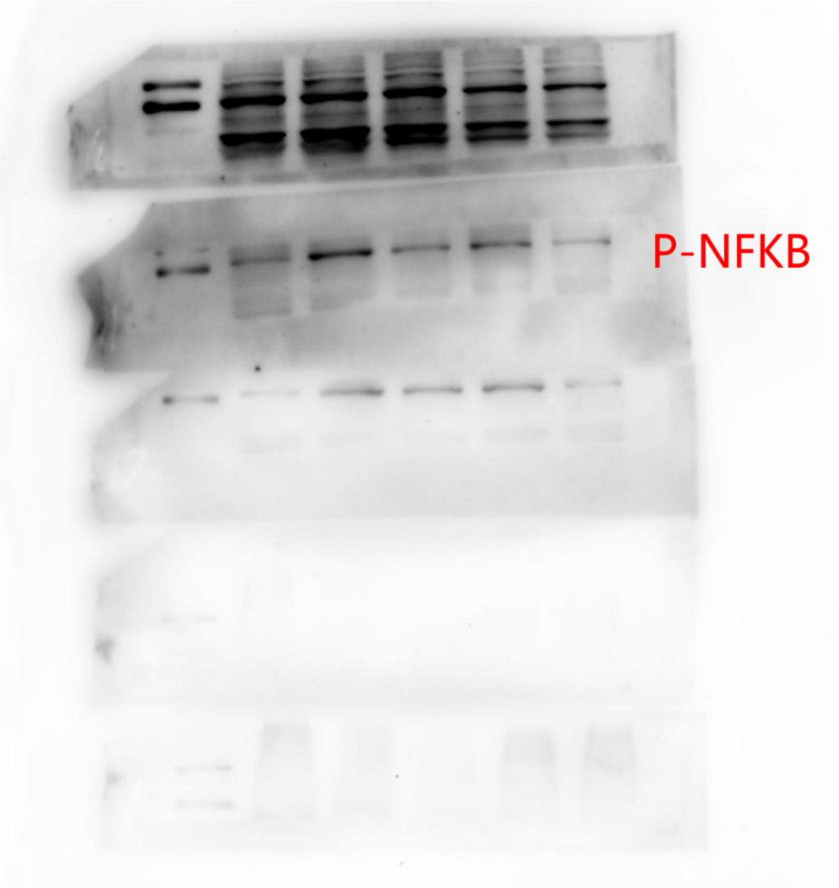

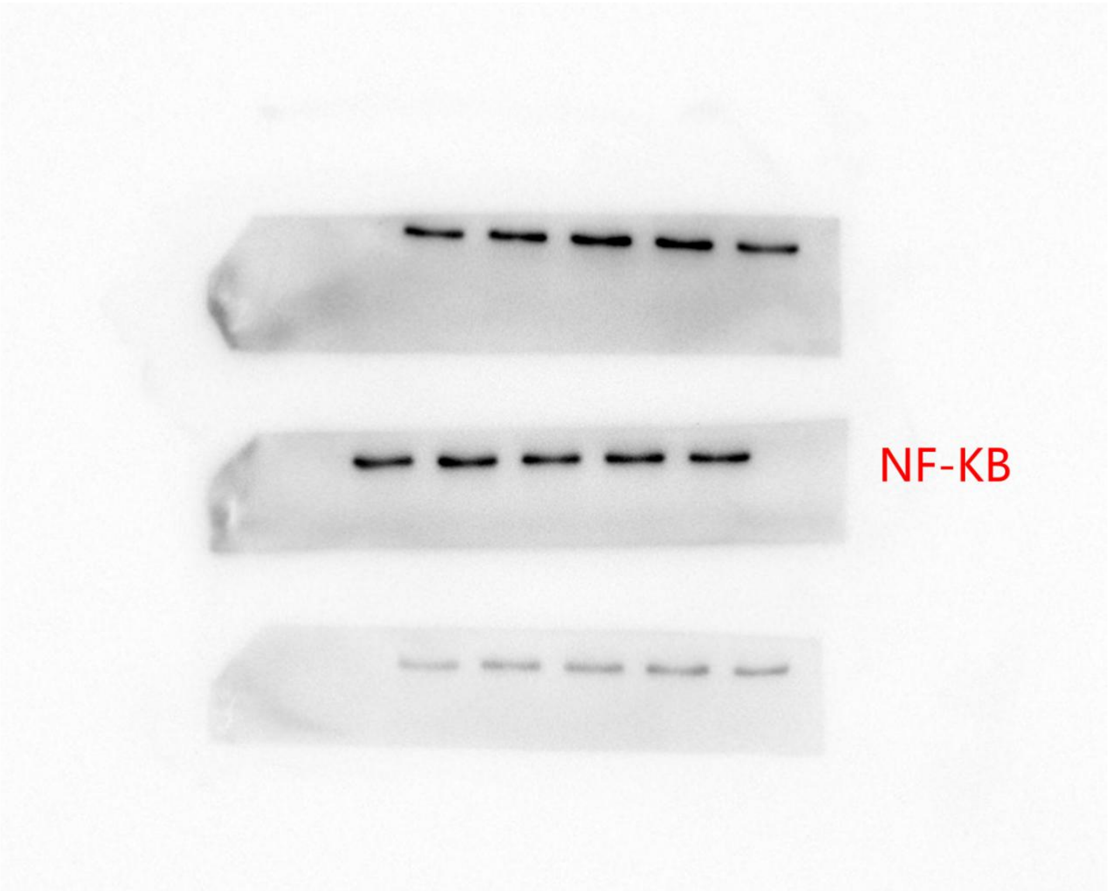

NF-KB

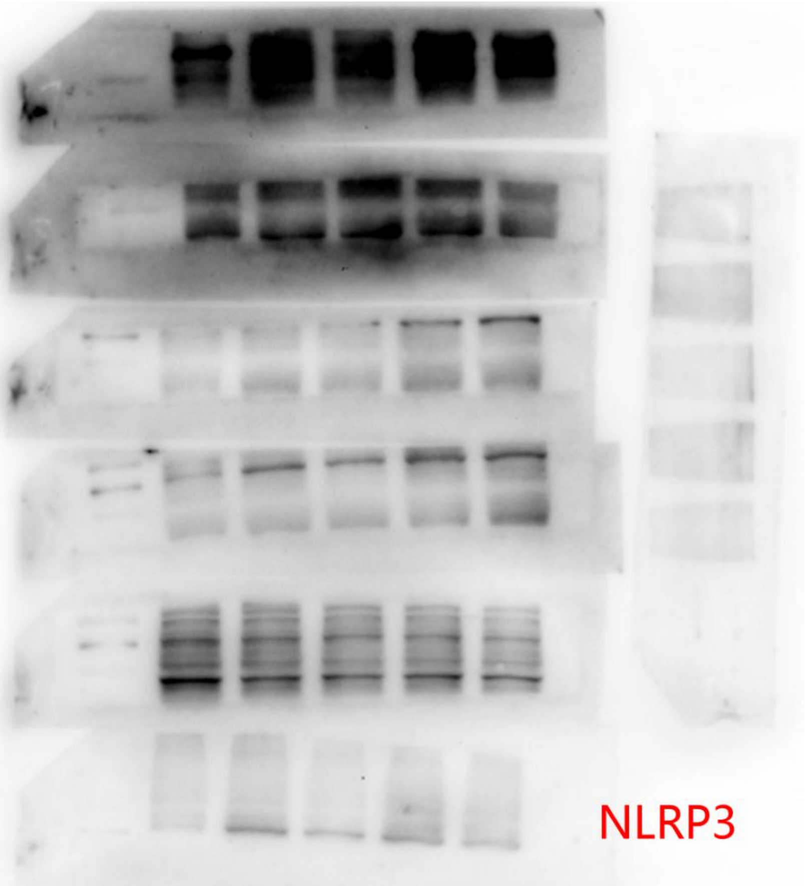

NLRP3

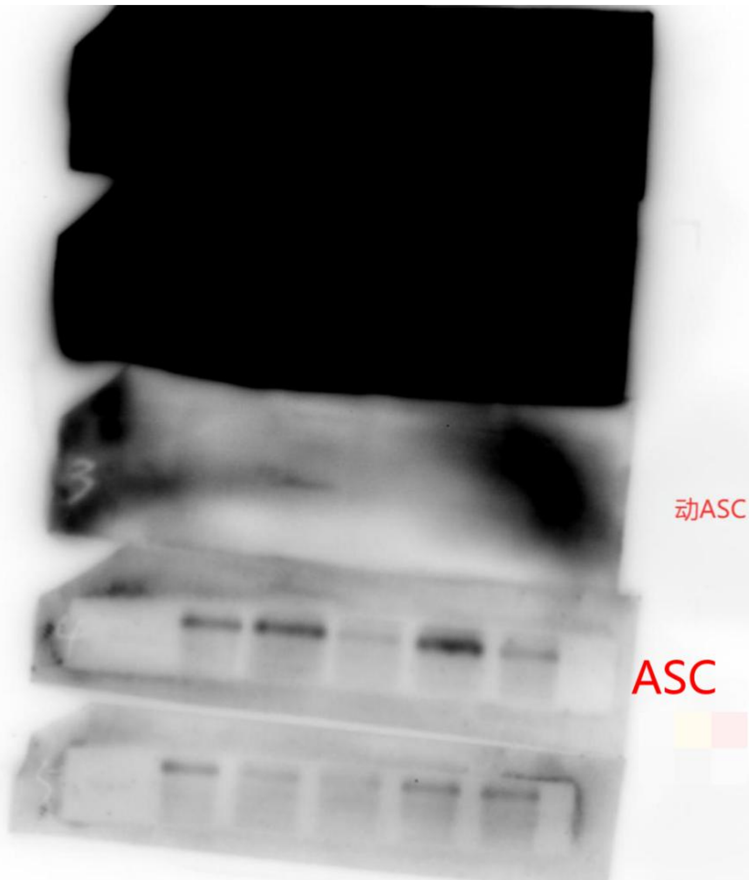

动ASC

ASC

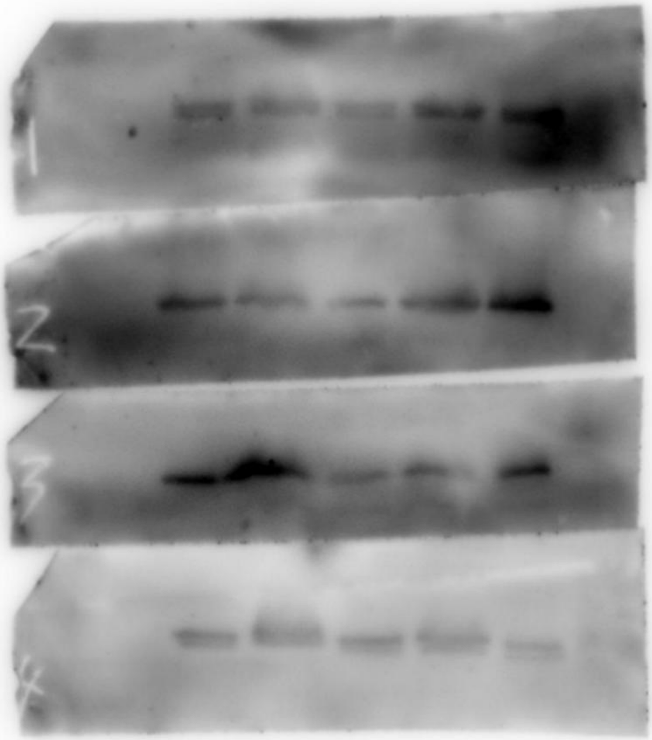

Cleaved caspase-1

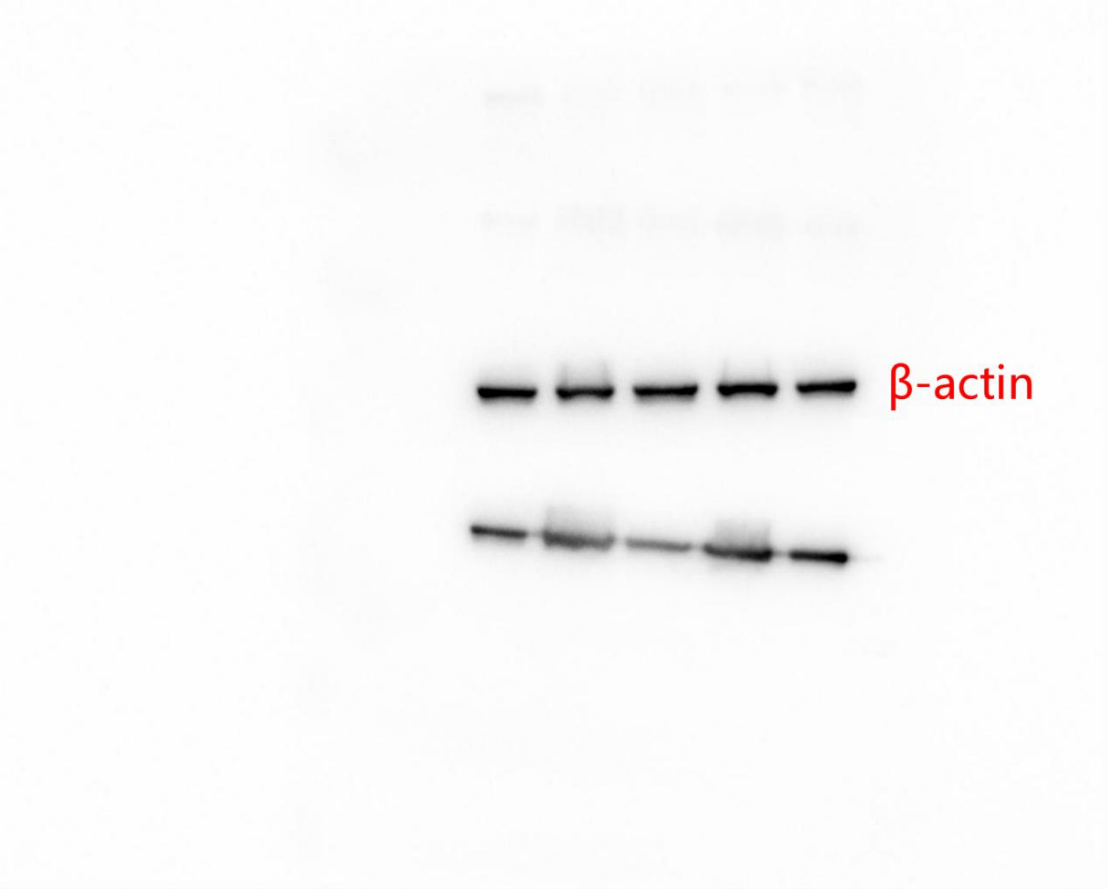

FIGURE 4 | Sal improved LPS-induced depression in mice.

The original western blot bands of BDNF, β-actin protein in LPS-induced depressive mice.

The lane order from left to right on blot is: (1) the control group (2) the LPS group, (3) the LPS + FLU group, (4) the LPS + Sal (20 mg/kg) group, (5) the LPS + Sal (40 mg/kg) group.

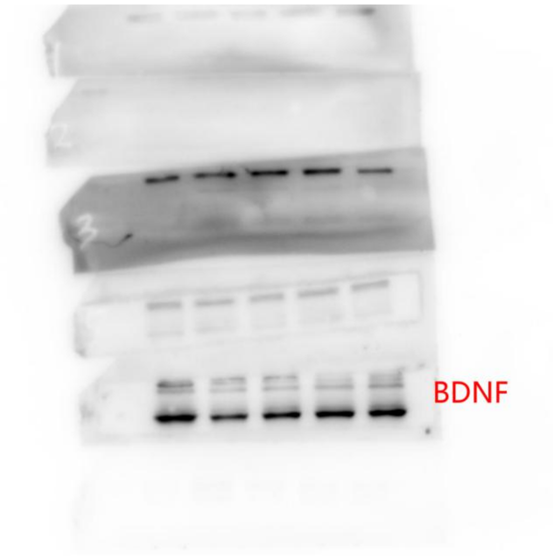

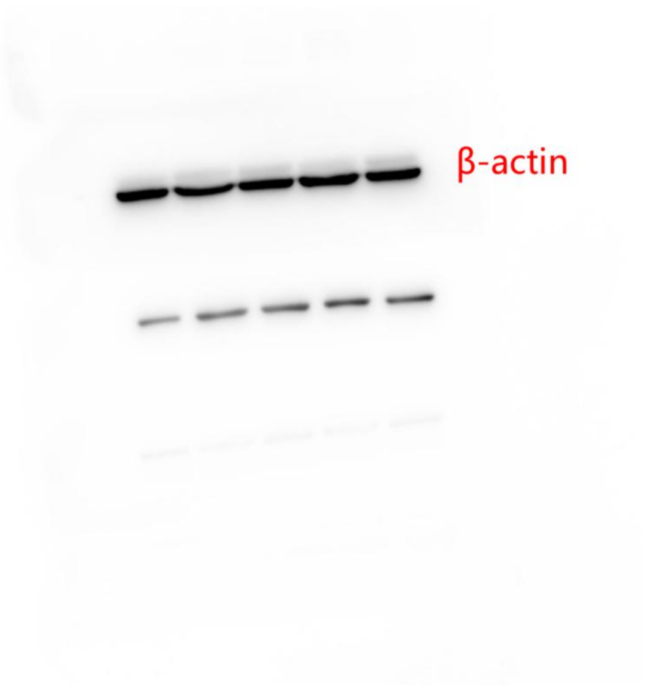

FIGURE 5 | Sal ameliorated pyroptosis in LPS-induced depression in mice.

The original western blot bands of IL-1β, IL-18, cleaved GSDMD and β-actin protein in LPS-induced depressive mice.

The lane order from left to right on blot is: (1) the control group, (2) the LPS group, (3) the LPS + FLU group, (4) the LPS + Sal (20 mg/kg) group, (5) the LPS + Sal (40 mg/kg) group.

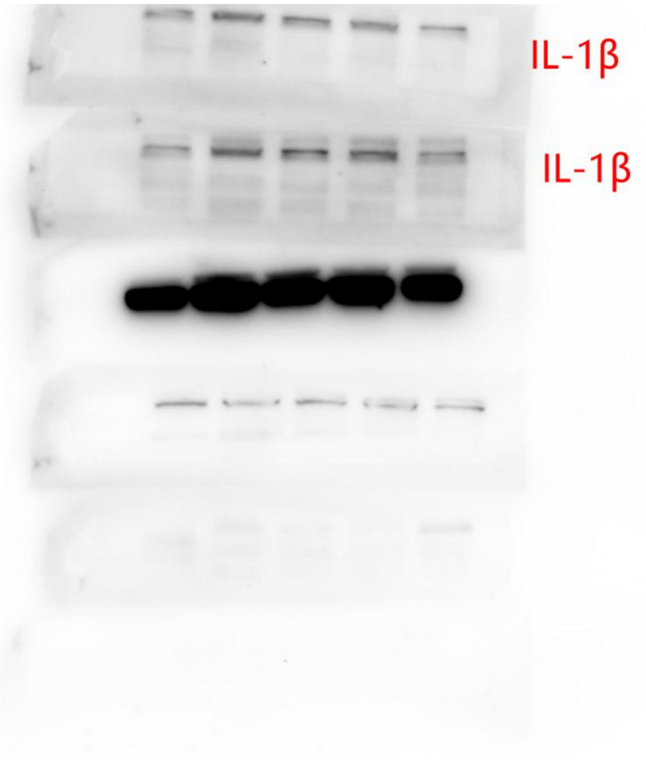

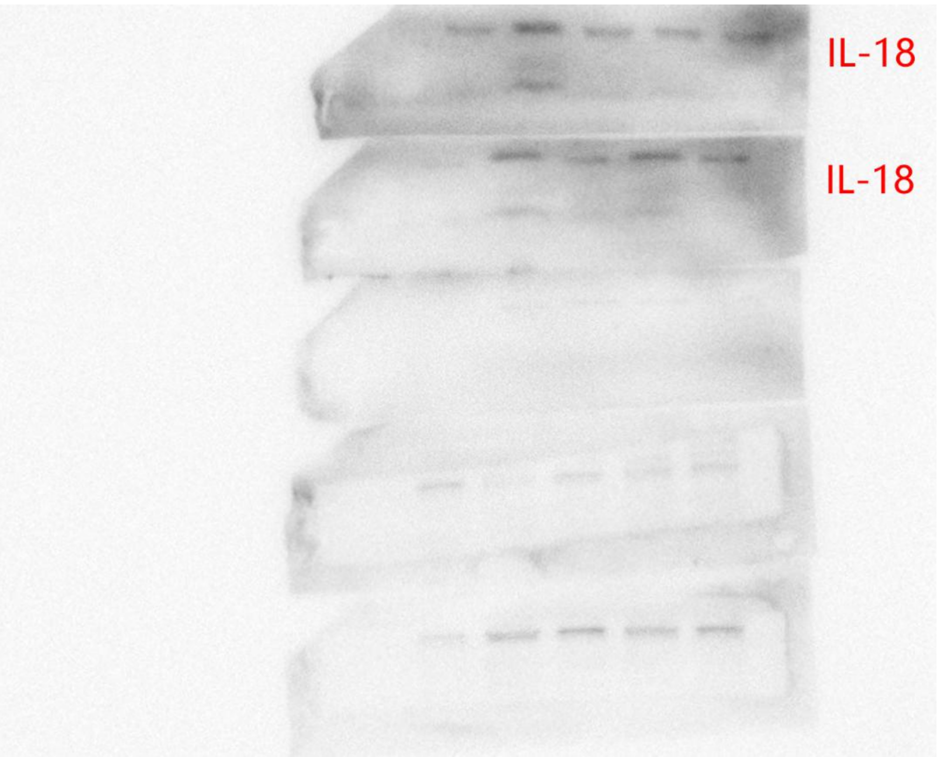
IL-18
IL-18

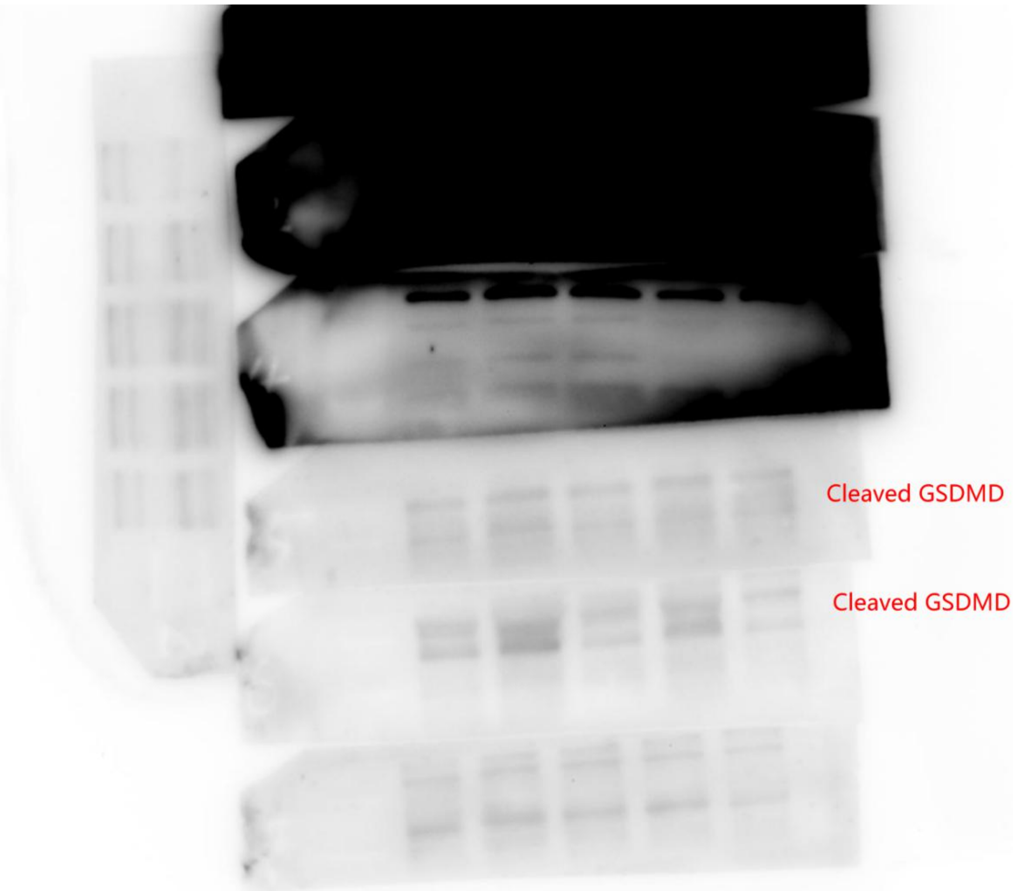
Cleaved GSDMD
Cleaved GSDMD

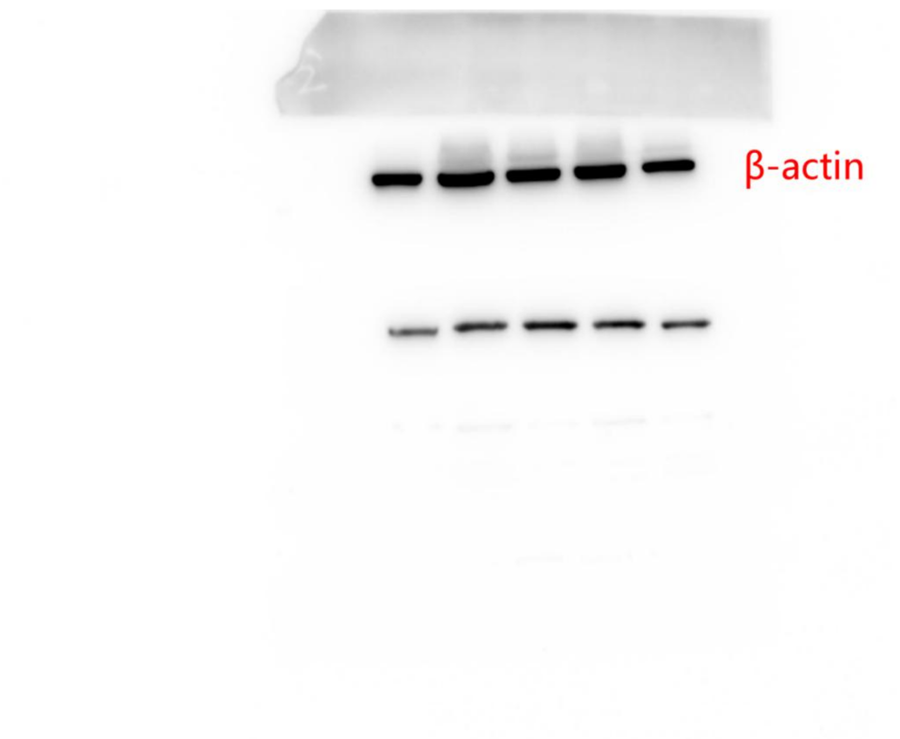

FIGURE 6 | Sal suppressed pyroptosis via the P2X7/NF-κB/NLRP3 signaling pathway in LPS-induced depression in mice.

The original western blot bands of P2X7, P-NF-κB, NF-κB, NLRP3, ASC, Cleaved caspase-1 and β-actin protein in LPS-induced depressive mice.

The lane order from left to right on blot is: (1) the control group, (2) the LPS group, (3) the LPS + FLU group, (4) the LPS + Sal (20 mg/kg) group, (5) the LPS + Sal (40 mg/kg) group.

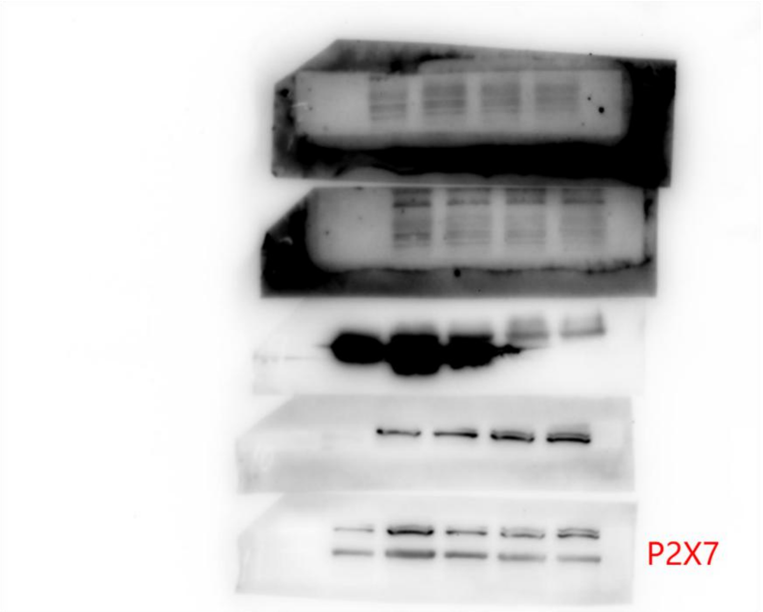

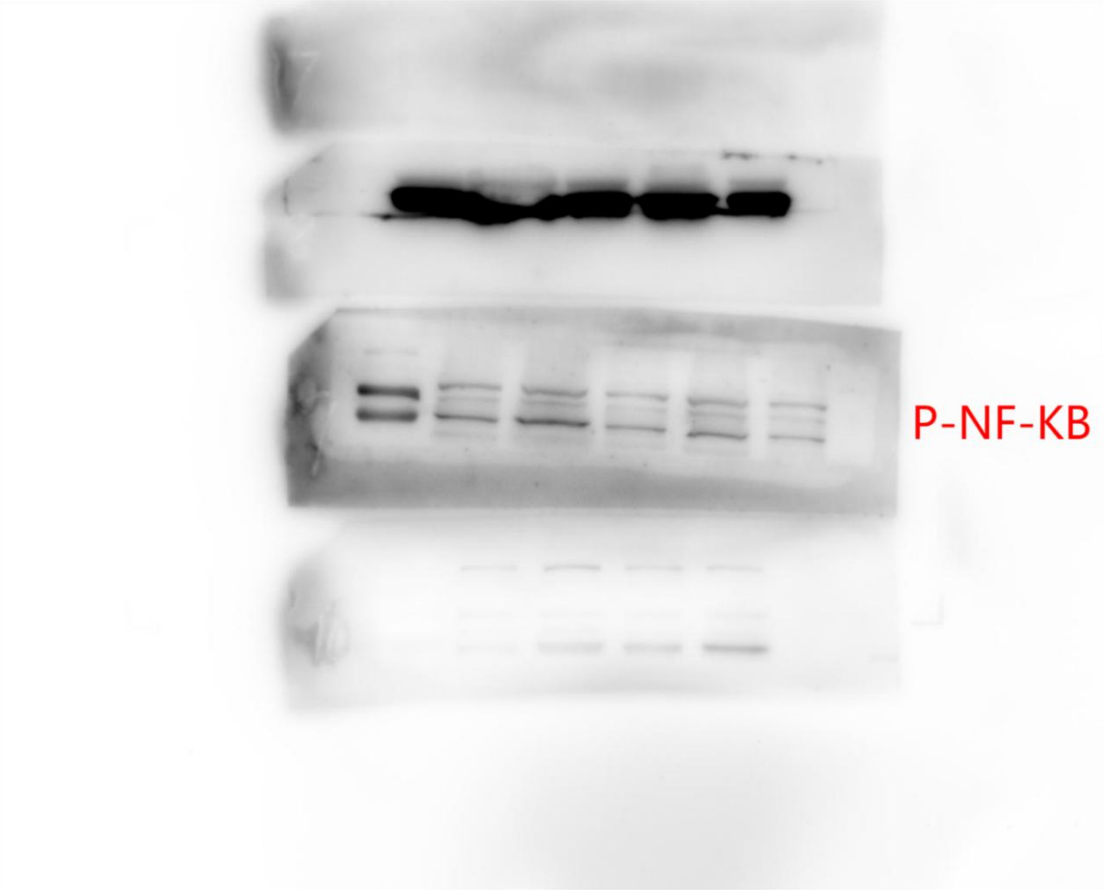

P-NF-KB

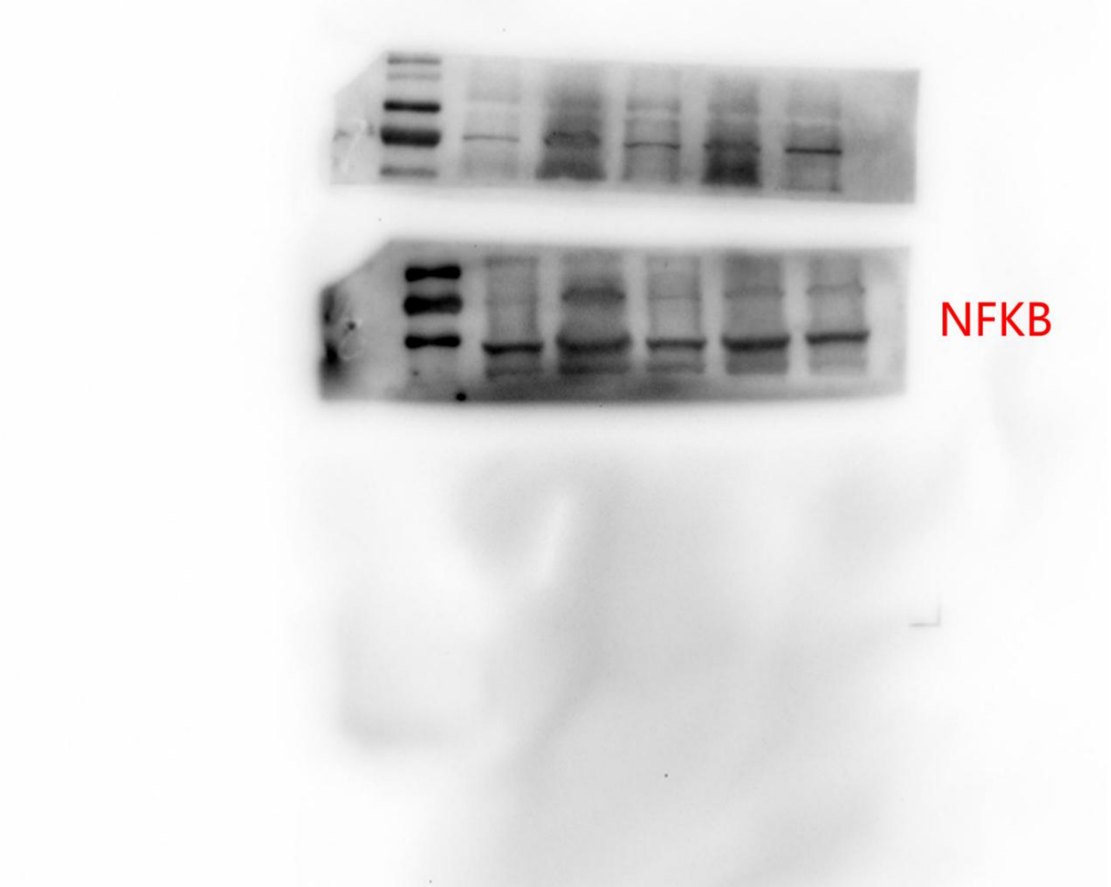

NFKB

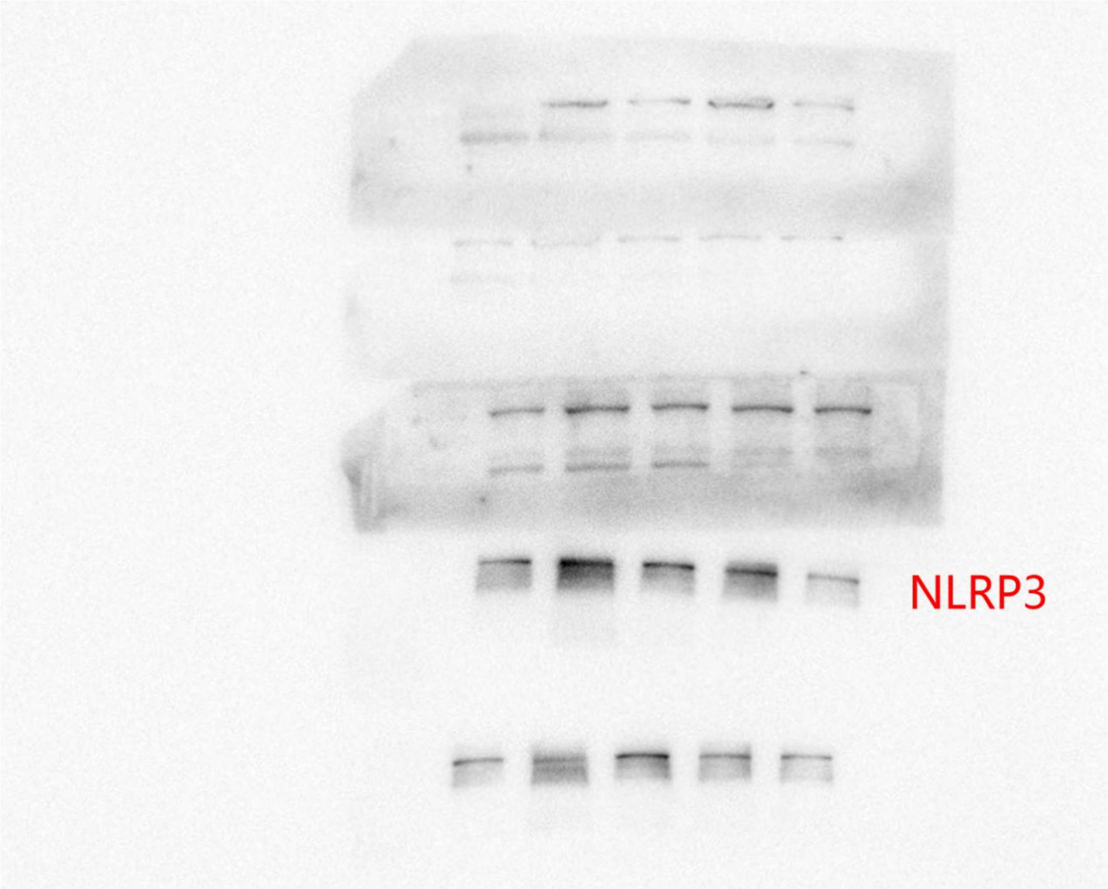
NLRP3

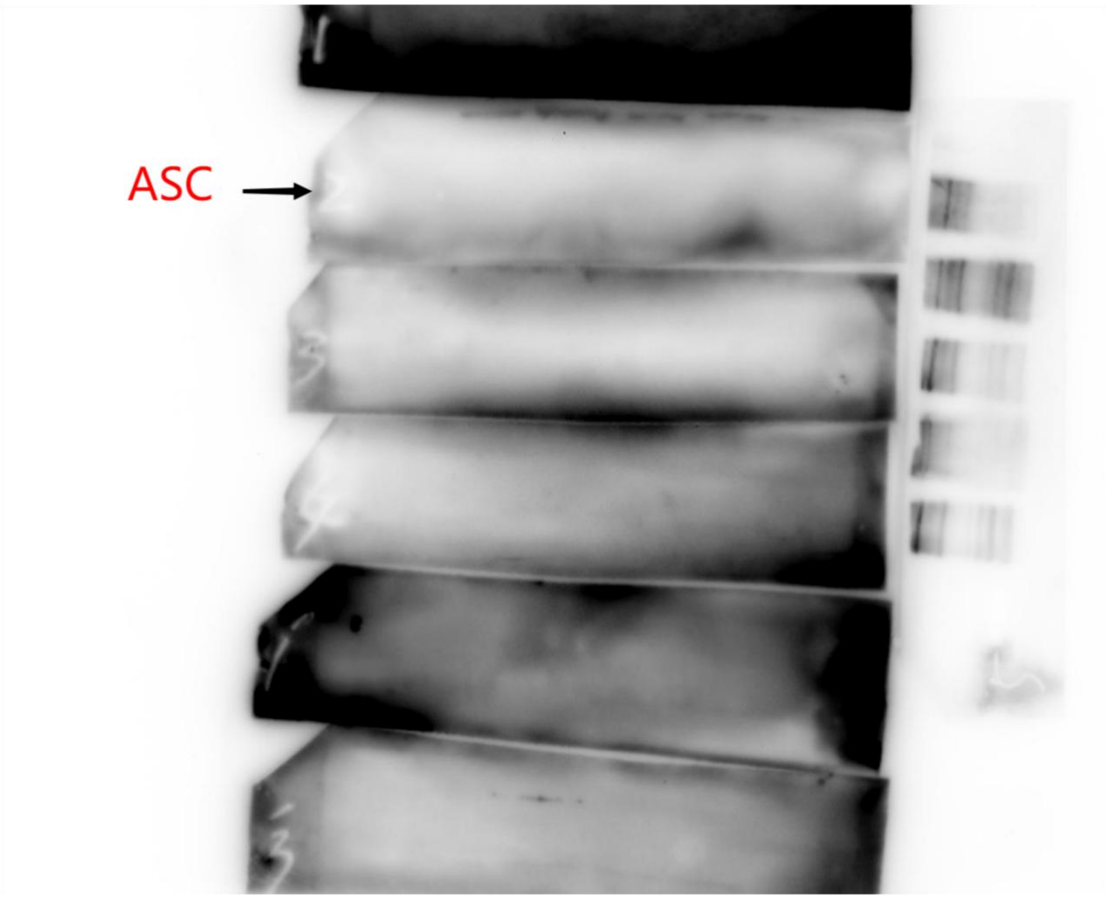
ASC

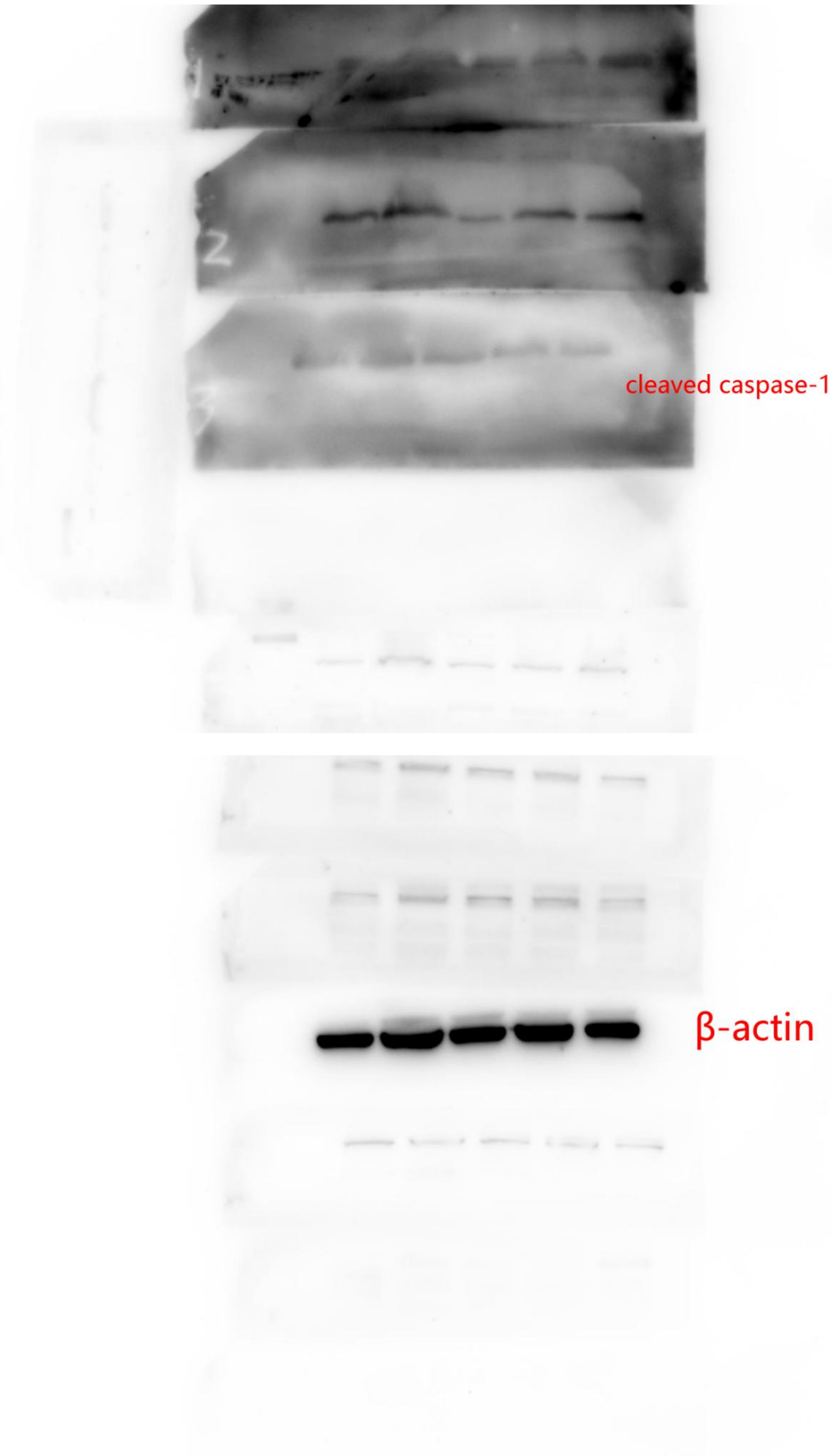

cleaved caspase-1
β-actin

FIGURE 7 | Sal ameliorated CORT-induced PC12 cells.

(B) Sal inhibited CORT-induced decreased BDNF in PC12 cells by Western blotting (n=3).

The original western blot bands of BDNF and β-actin protein in CORT-induced PC12 cells.

The lane order from left to right on blot is: (1) the control group, (2) the CORT group, (3) the CORT + Sal (2 μM) group, (4) the CORT + Sal (10 μM) group, (5) the CORT + Sal (40 μM) group.

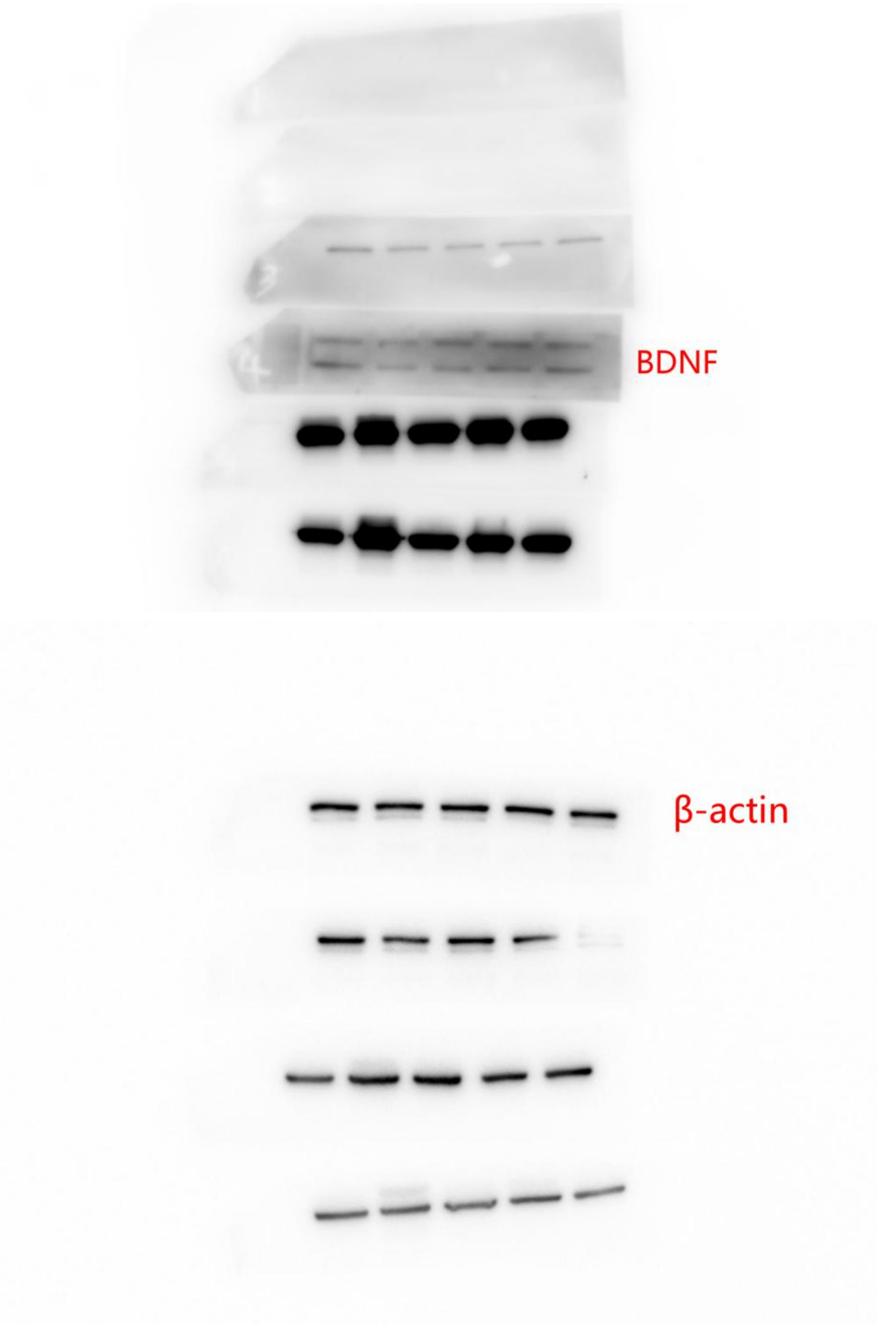

(D) Sal inhibited CORT-induced decreased BDNF in PC12 cells by Western blotting (n=3).

The original western blot bands of cleaved GSDMD, IL-1β, IL-18 and β-actin protein in CORT-induced PC12 cells.

The lane order from left to right on blot is: (1) the control group, (2) the CORT group, (3) the CORT + Sal (2 μM) group, (4) the CORT + Sal (10 μM) group, (5) the CORT + Sal (50 μM) group.

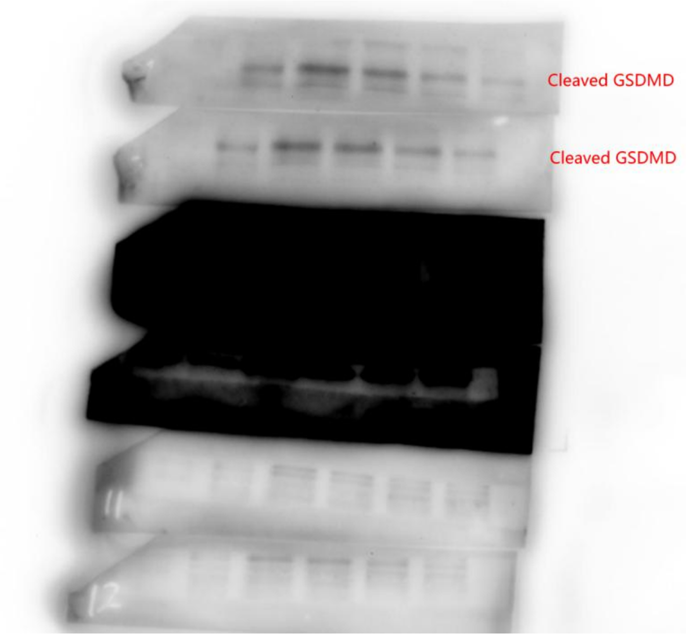

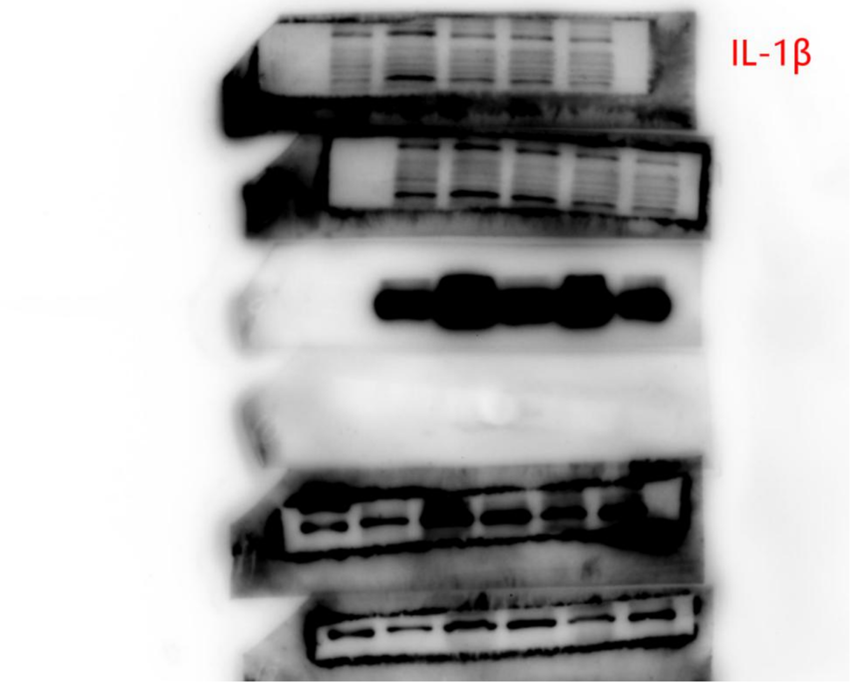

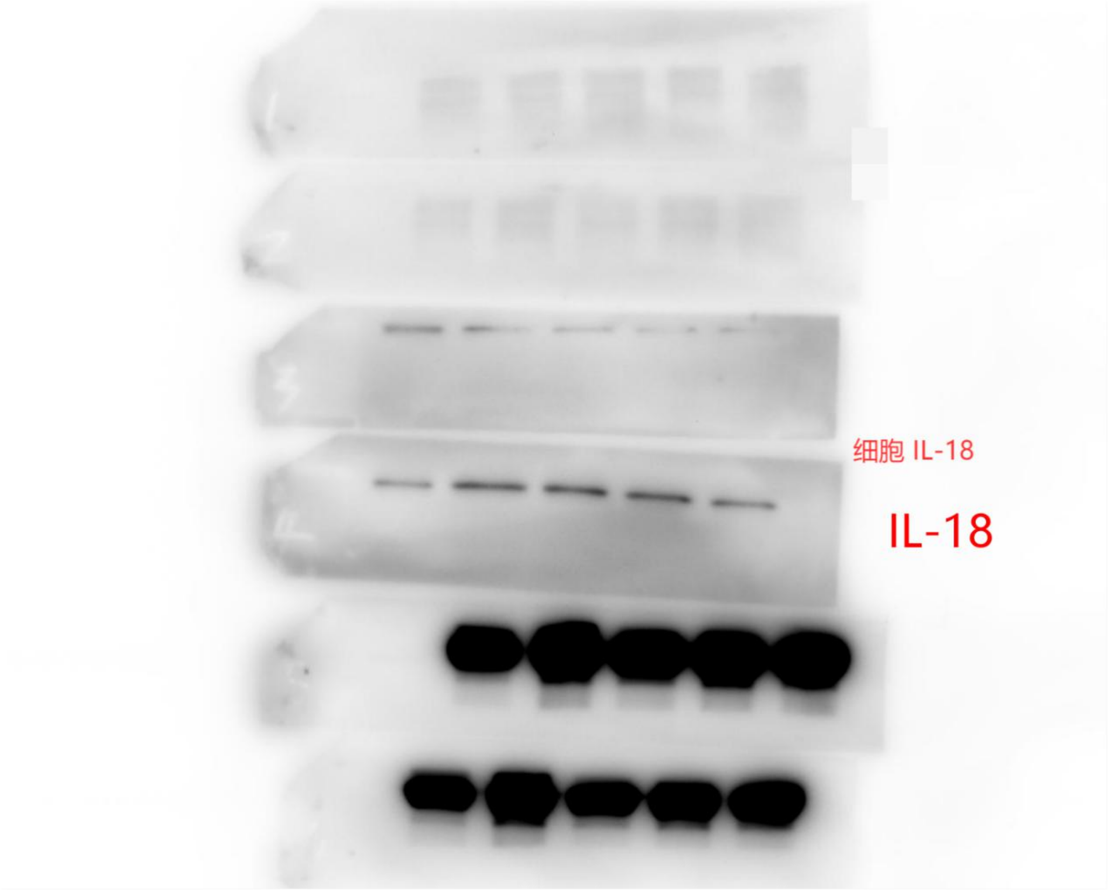
细胞 IL-18
IL-18

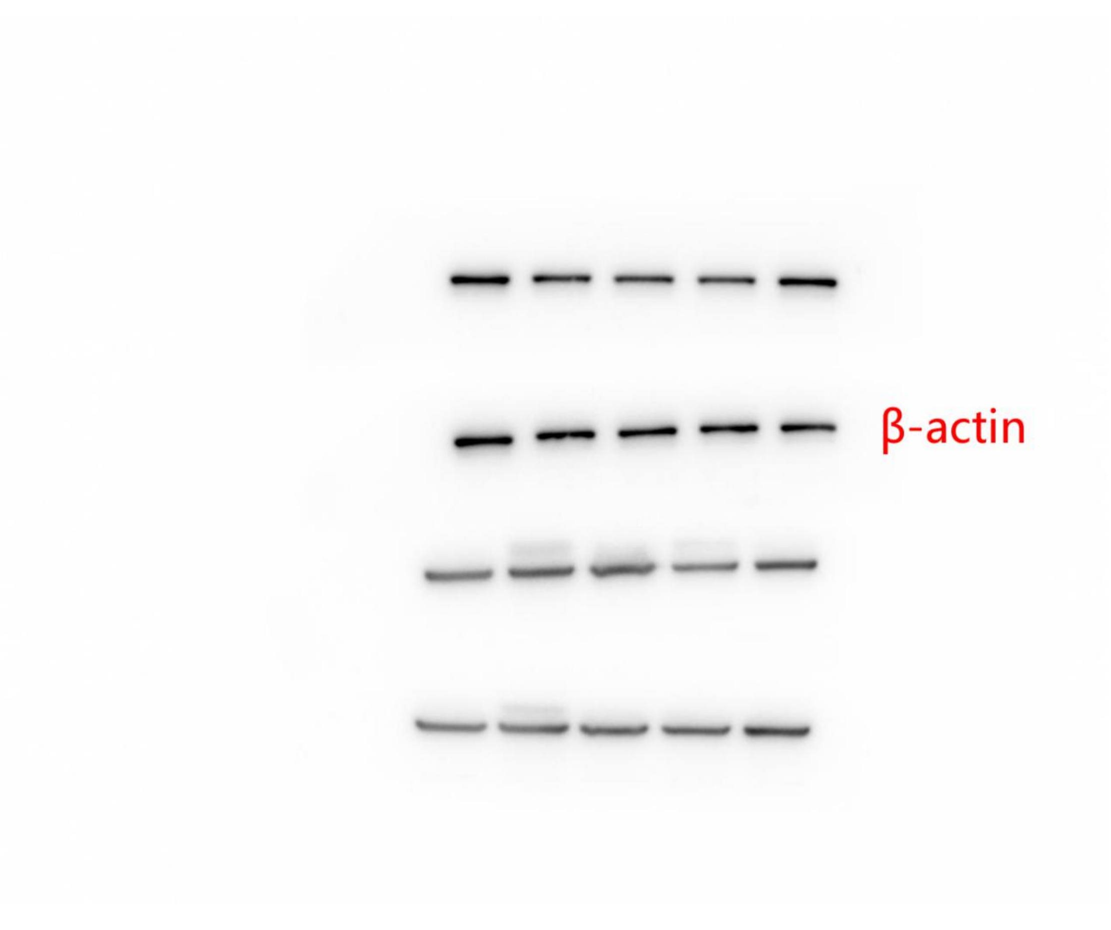
β-actin

FIGURE 8 | Sal inhibited CORT-induced PC12 cells pyroptosis via P2X7/NF-κB/NLRP3 signaling pathway.

(A) Sal inhibited P2X7, P-NF-κB, NLRP3, ASC, Cleaved caspase-1 in PC12 cells supernatant by Western blotting (n=3).

The original western blot bands of P2X7, P-NF-κB, NF-κB, NLRP3, ASC, Cleaved caspase-1 and β-actin protein in CORT-induced PC12 cells.

The lane order from left to right on blot is: (1) the control group, (2) the CORT group, (3) the CORT + Sal (2 μM) group, (4) the CORT + Sal (10 μM) group, (5) the CORT + Sal (50 μM) group.

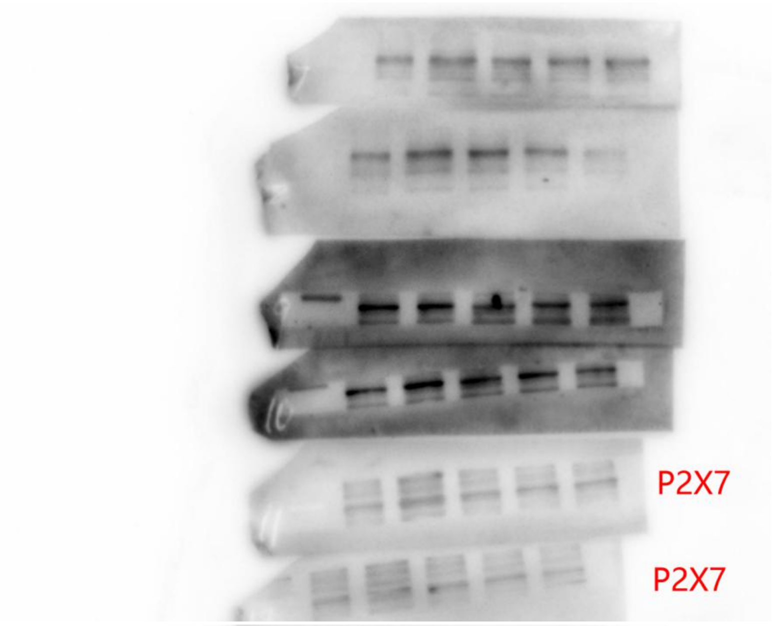

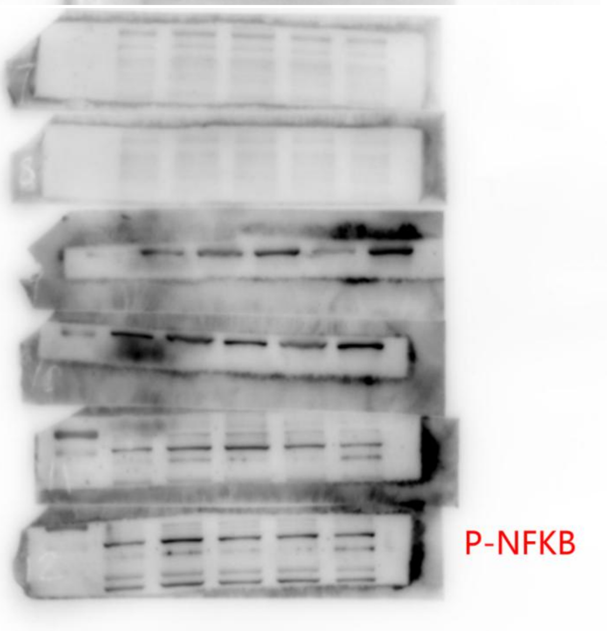

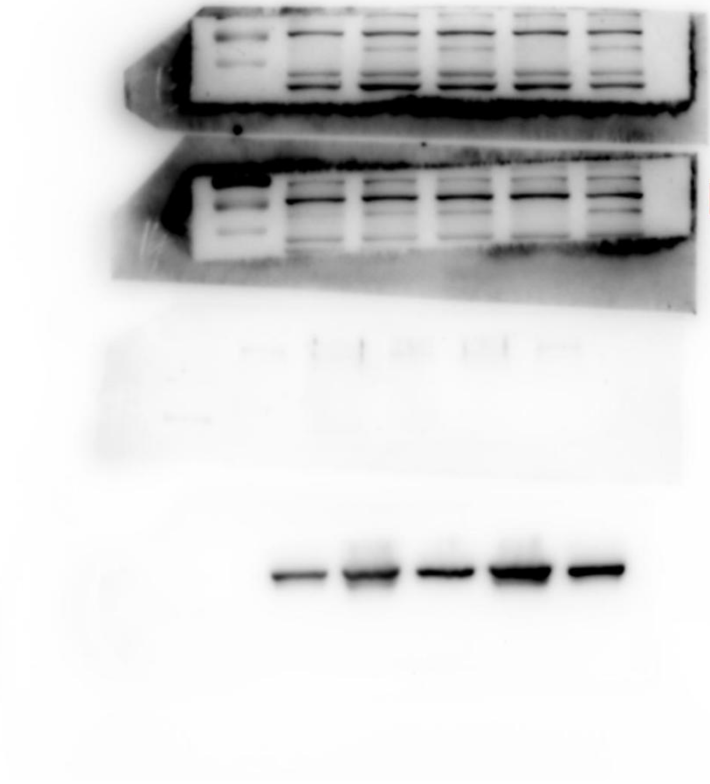
NFKB

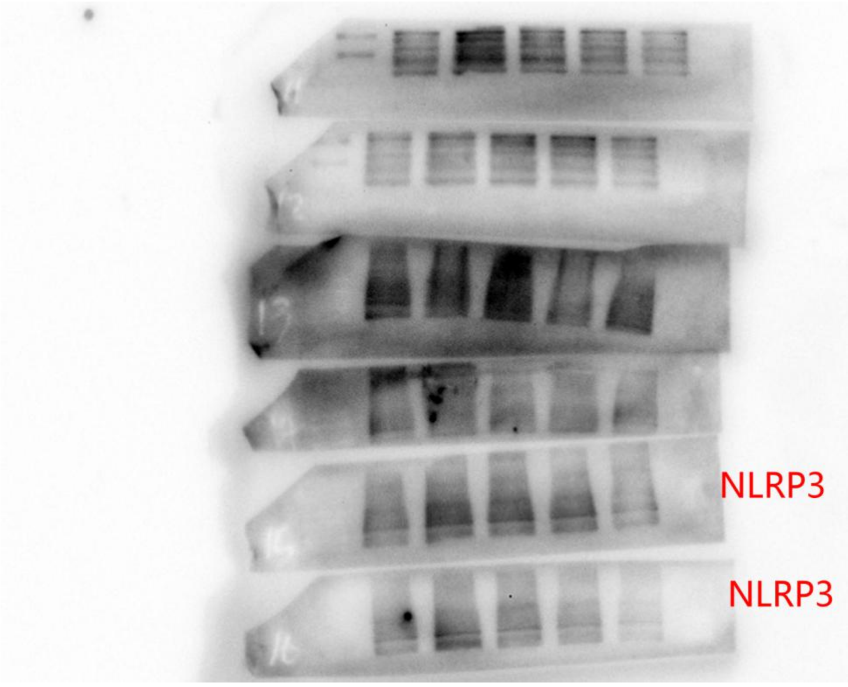

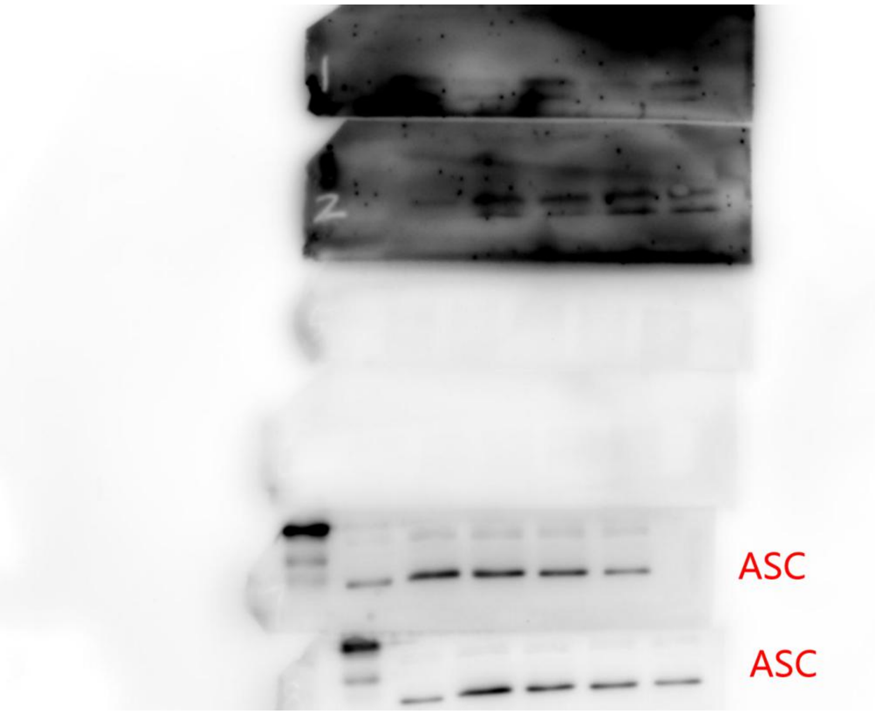
ASC
ASC

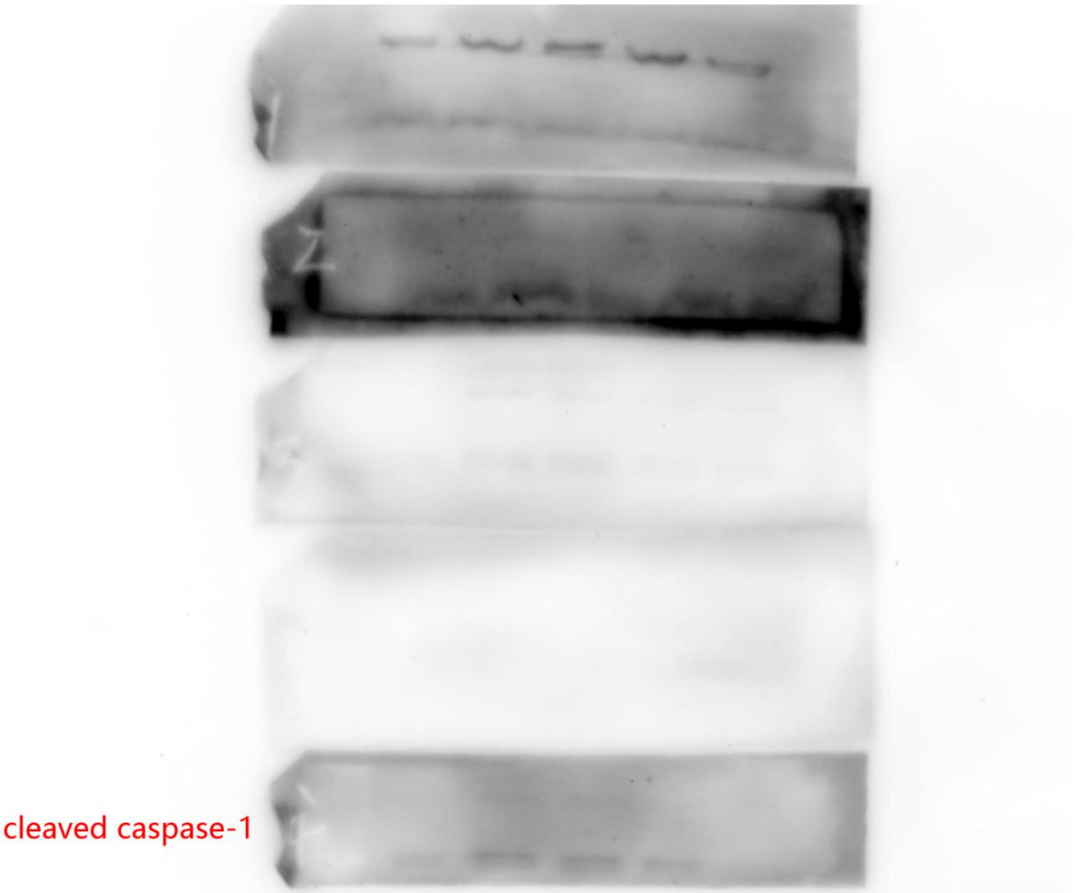
cleaved caspase-1

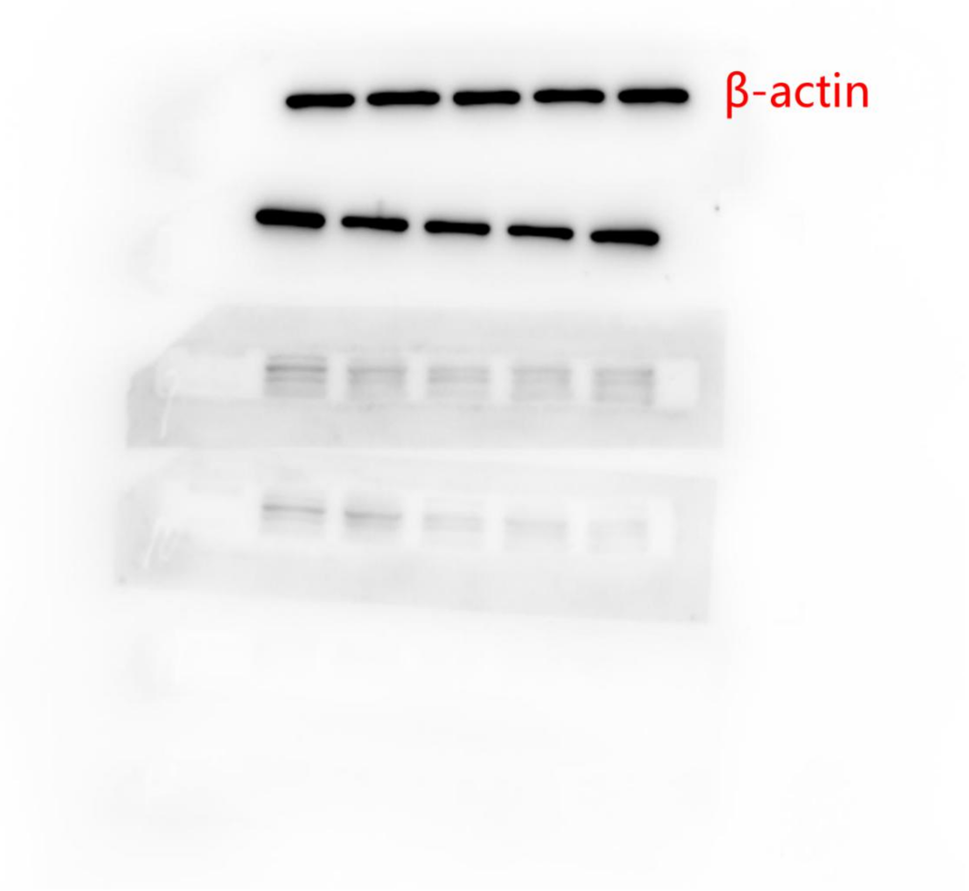

(E) NLRP3 plays a vital role in CORT-induced pyroptosis: NLRP3 agonist reversed the protective effect of Sal on CORT-induced PC12 cells (n=3).

The original western blot bands of NLRP3, ASC, Cleaved caspase-1, Cleaved GSDMD, IL-18, IL-1β and β-actin protein in CORT-induced PC12 cells.

The lane order from left to right on blot is: (1) the control group; (2) the 200 μM CORT group; (3) the 200 μM CORT + 50 μM Sal (CORT + Sal) group; (4) the 200 μM CORT + 50μM Sal + 10 μM nigericin (CORT + Sal + Nig)

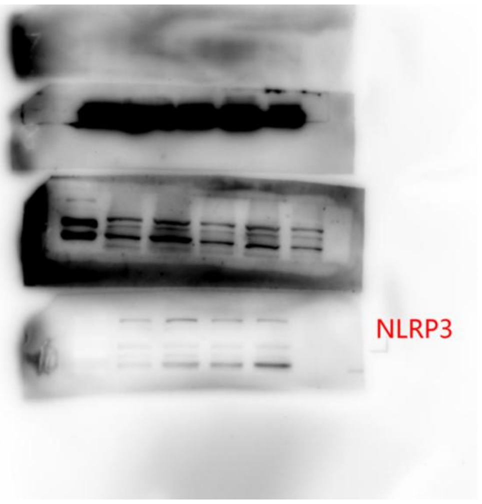

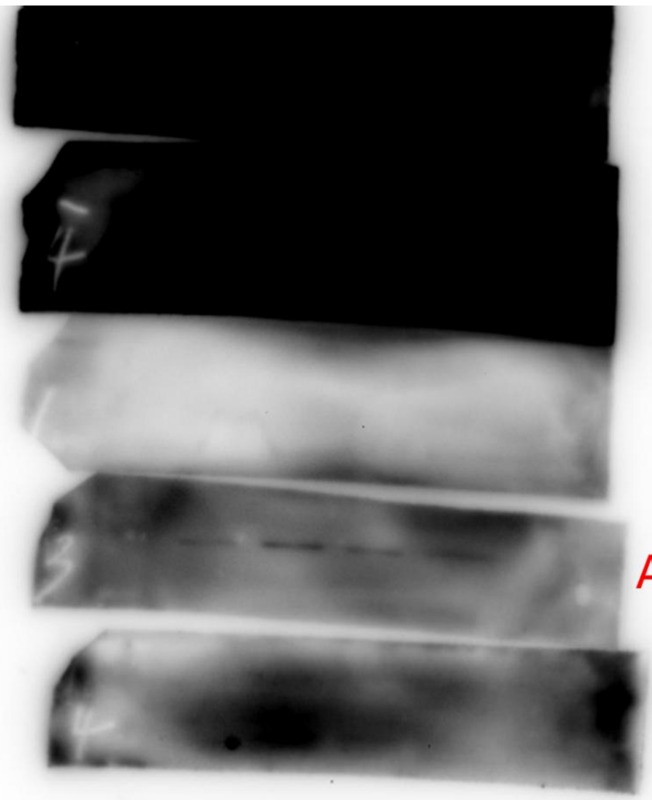

ASC

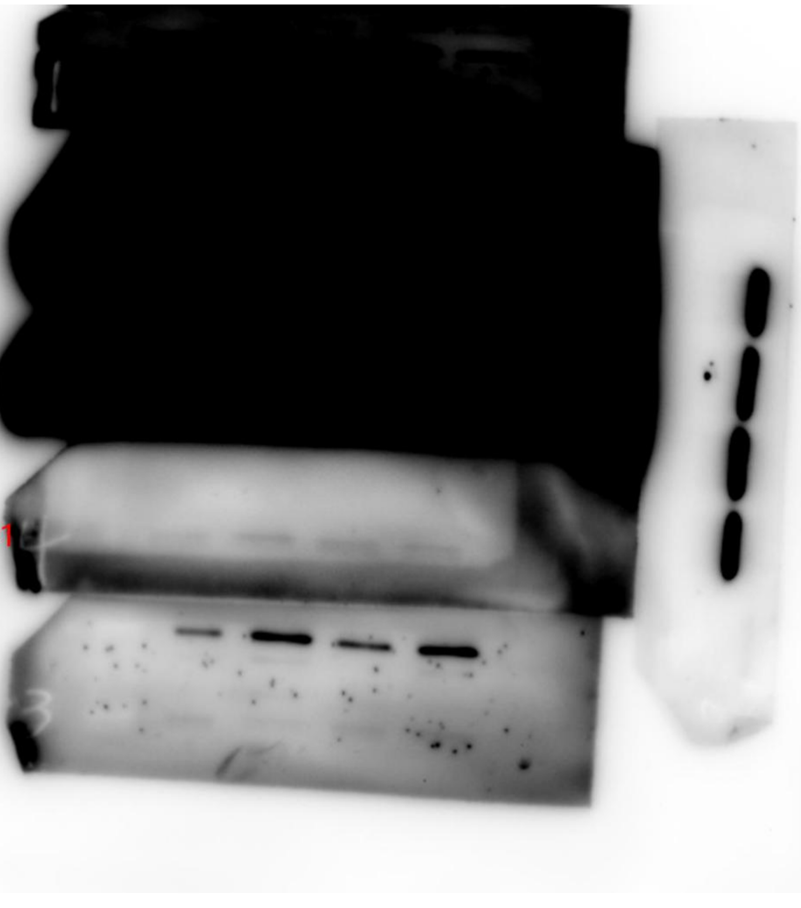

Cleaved caspase-1

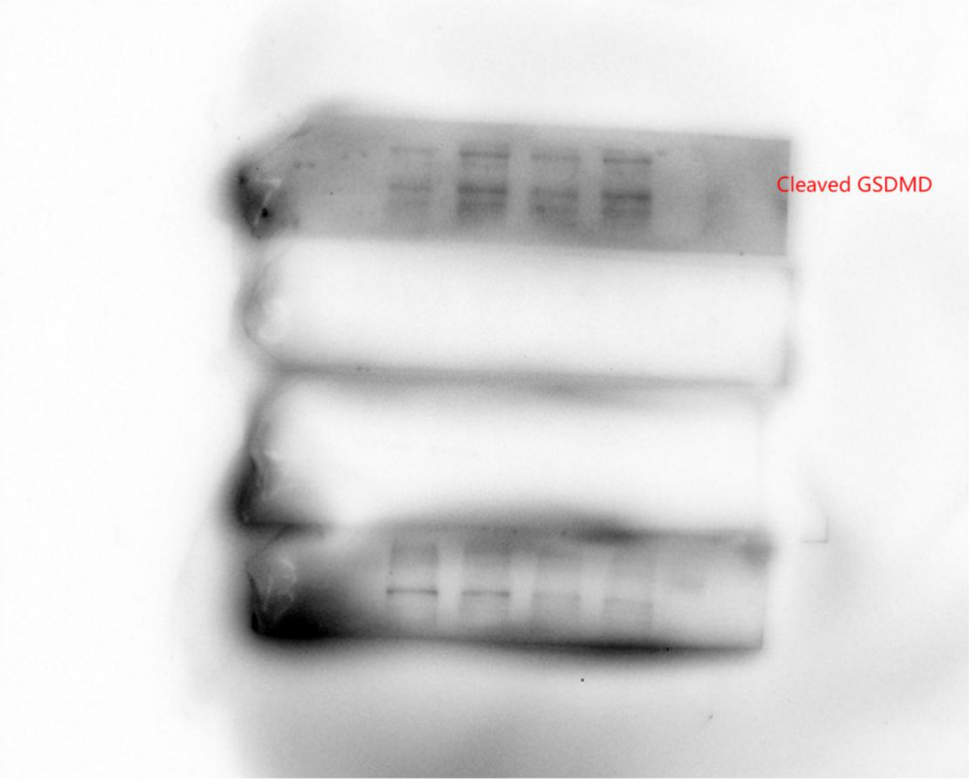
Cleaved GSDMD

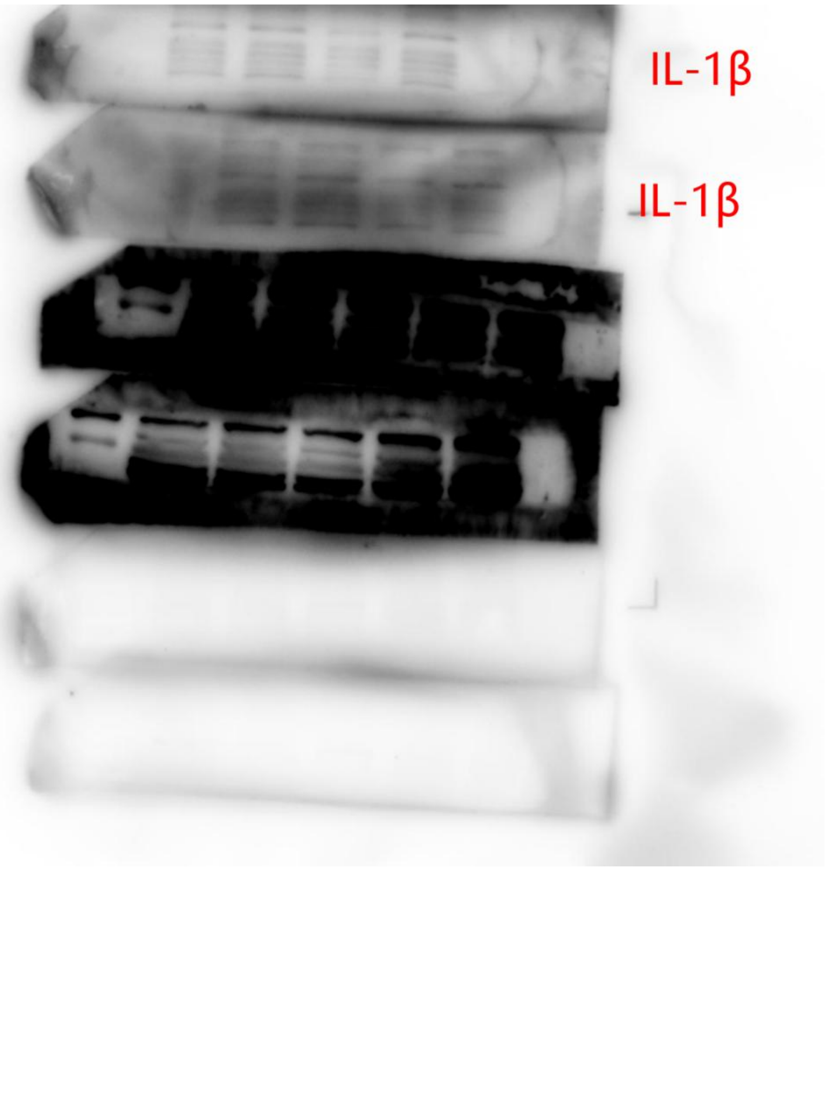
IL-1β
IL-1β

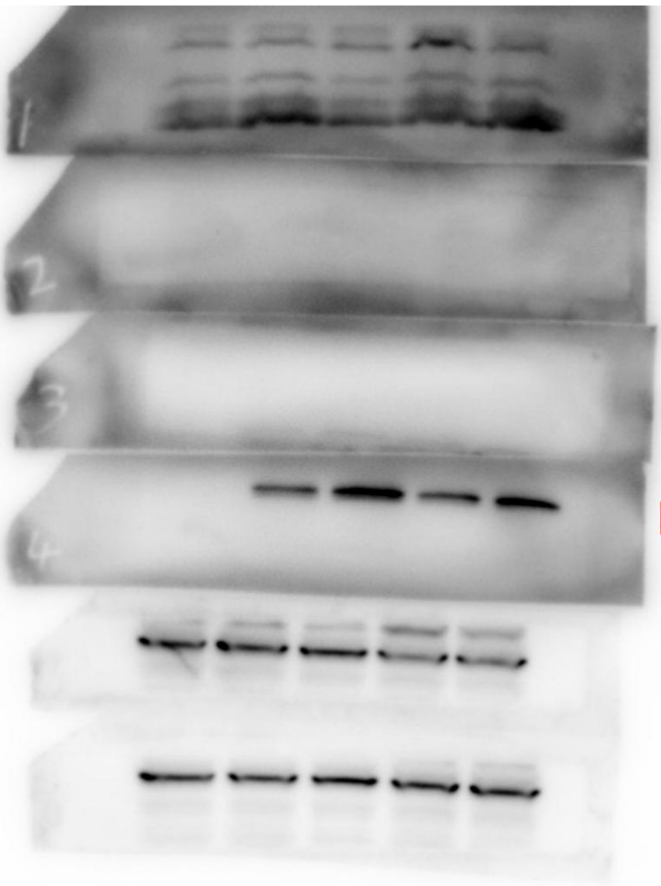

IL-18

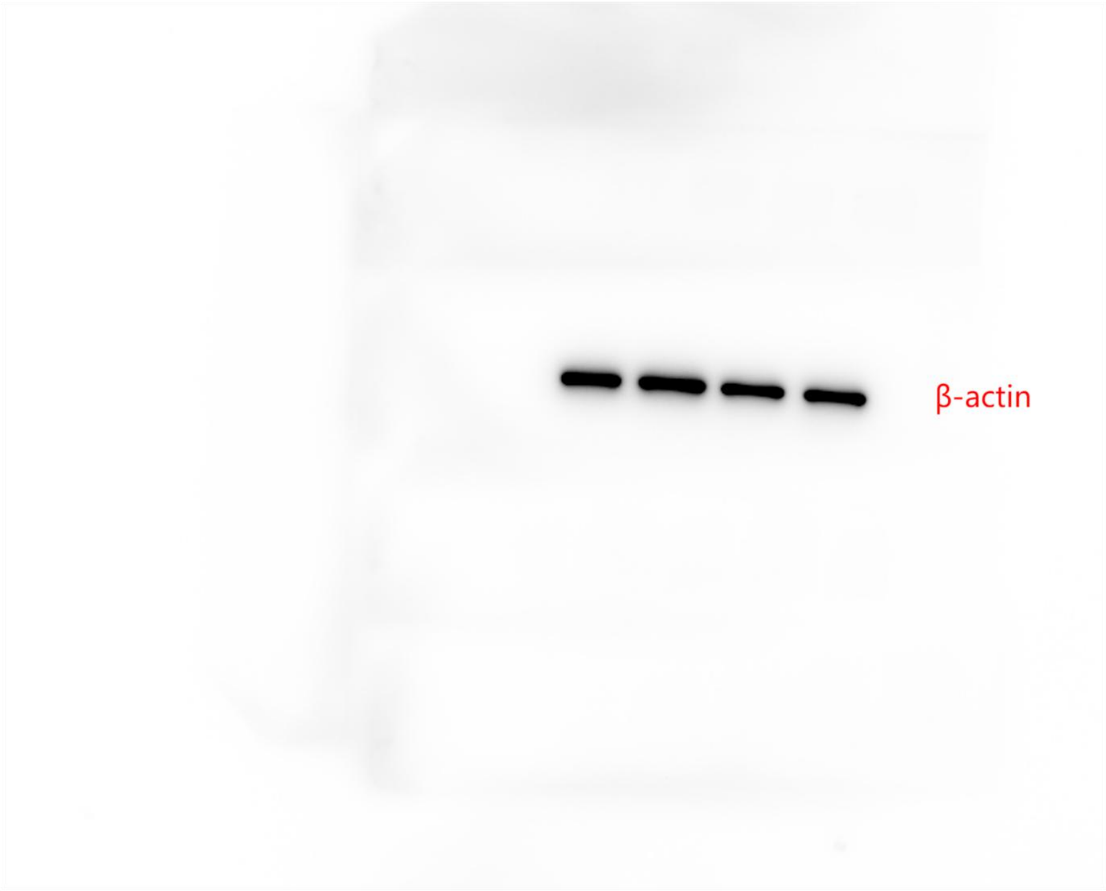

β-actin
