# Supplementary material for: Salidroside Ameliorates Depression by Suppressing NLRP3-Mediated Pyroptosis via P2X7/NF-κB/NLRP3 Signaling Pathway
Source: Front Pharmacol. 2022 Apr 12;13:812362. doi: 10.3389/fphar.2022.812362 (PMC9039222; doi:10.3389/fphar.2022.812362)
Supplement: Supplementary file 1 [file DataSheet1.ZIP › Supplementary Materials/Supplementary materials Supplementary Table S1.pdf]

Supplementary materials：

Supplementary Table S1. Main materials and reagents

Reagents	Source	Identifier
Corticosterone	Aladdin	C104537
Lipopolysaccharide	Sigma	L2630
Fluoxetine	Aladdin	F131623
Salidroside	Meilunbio	MB5843-1
Nigericin	MCE	HY-127019
Sucrose	Aladdin	S112231
PMSF	Aladdin	P105539
Tween 20	Aladdin	T104863
Tween-80	Aladdin	T118633
RIPA	Beyotime	P0013B
1.5M Tris	Beyotime	ST789
1.0M Tris	Beyotime	ST768
10% SDS	Beyotime	ST628
Protease and phosphatase inhibitor	Beyotime	P1045
PVDF	Milipore	IPVH00010
Skim milk	BD	232100
TBST	Beyotime	ST673
ECL western blotting detection reagents	Tanon	180-5001
DAPI	Thermo Fisher	R37606
PBS	Beyotime	ST447
Triton X-100 Solution	Beyotime	ST797
Fetal Bovine Serum	Gibco	10099-141
Dulbecco's Modified Eagle Medium	Gibco	C11995500BT
Cell counting kit-8	Beyotime	C0039
BCA protein assay kit	Beyotime	P0010
Rat BDNF ELISA Kit	Elabscience	E-EL-R1235c
Mouse BDNF ELISA Kit	Elabscience	E-EL-M0203c
Rat IL-18 ELISA Kit	Elabscience	E-EL-R0567c
Mouse IL-18 ELISA Kit	Elabscience	E-EL-M0730c
Rat IL-1β ELISA Kit	Elabscience	E-EL-R0012c
Mouse IL-1β ELISA Kit	Elabscience	E-EL-M0037c
Rabbit Polyclonal anti-BDNF	Proteintech	28205-1-AP
Rabbit Polyclonal anti-P2X7	Proteintech	28207-1-AP
Rabbit Polyclonal anti-ASC	Proteintech	10500-1-AP
Rabbit Polyclonal anti-IL-18	Proteintech	10663-1-AP
Rabbit Polyclonal anti-IL-1β	Proteintech	16806-1-AP
Rabbit polyclonal anti-NLRP3	Abcam	ab214185
Rabbit monoclonal anti-NF-κB p65	Cell Signaling Technology	#8242
Rabbit Polyclonal anti-Cleaved caspase-1	Thermo Fisher	PA5-99390
Rabbit monoclonal anti-P-NF-κB p65	Cell Signaling Technology	#3033

Rabbit polyclonal anti-Gasdermin D	Cell Signaling Technology	#93709
Mouse monoclonal anti-β-actin	Proteintech	66009-1-AP
Anti-rabbit IgG, HRP-linked Antibody	Cell Signaling Technology	#7074
Anti-mouse IgG, HRP-linked Antibody	Cell Signaling Technology	#7076
Anti-rabbit IgG (H+L) Alexa Fluor 488	Thermo Fisher	A11008
