# Supplementary figures and images for: Salidroside Ameliorates Depression by Suppressing NLRP3-Mediated Pyroptosis via P2X7/NF-κB/NLRP3 Signaling Pathway
Source: Front Pharmacol. 2022 Apr 12;13:812362. doi: 10.3389/fphar.2022.812362 (PMC9039222; doi:10.3389/fphar.2022.812362)

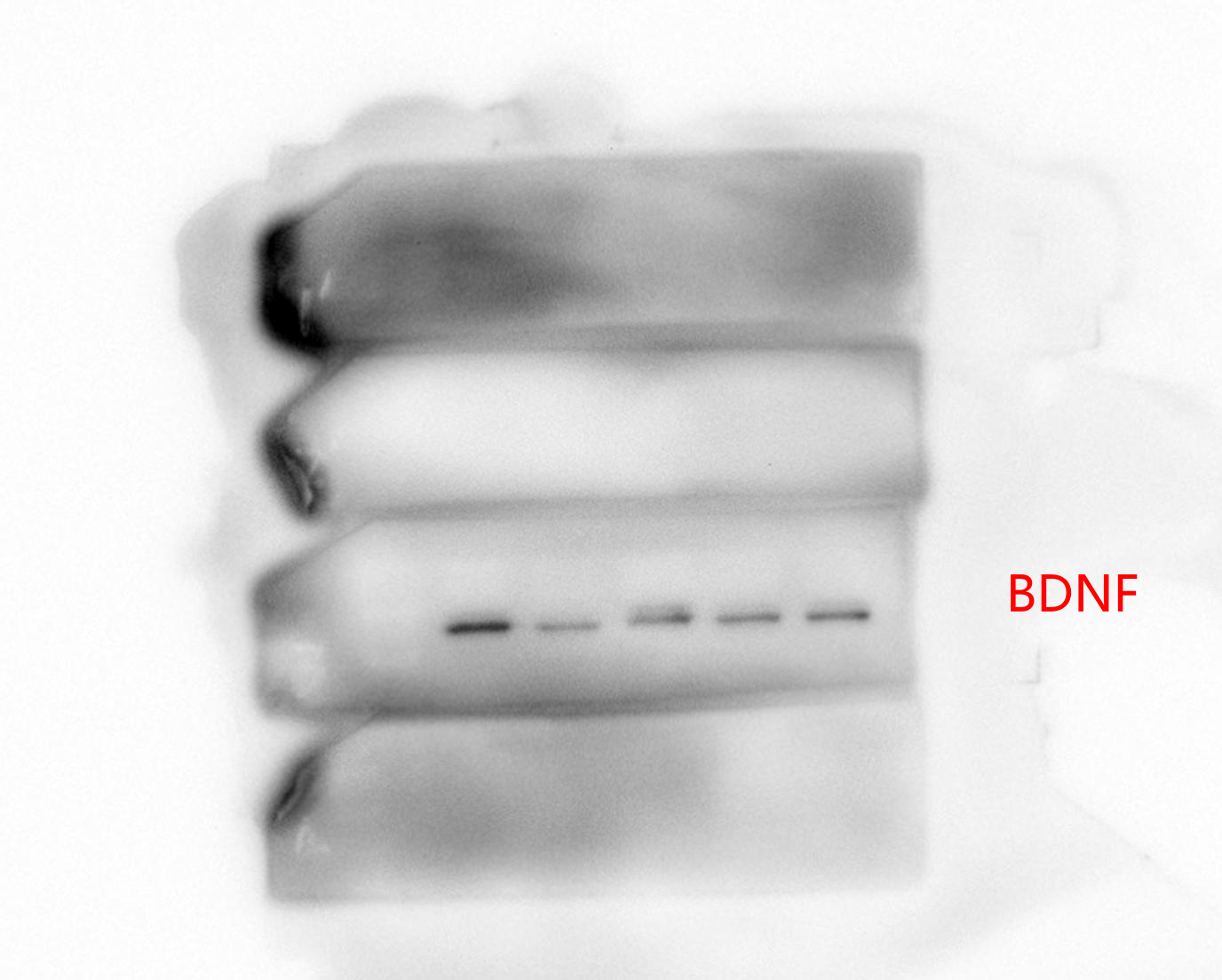

Supplement: Supplementary file 1 [file DataSheet1.ZIP › Supplementary Materials/supplementary materials ( original western blot figures)/Figure 1(original western blot figures)/BDNF(hippocampus of CORT model) .tif]

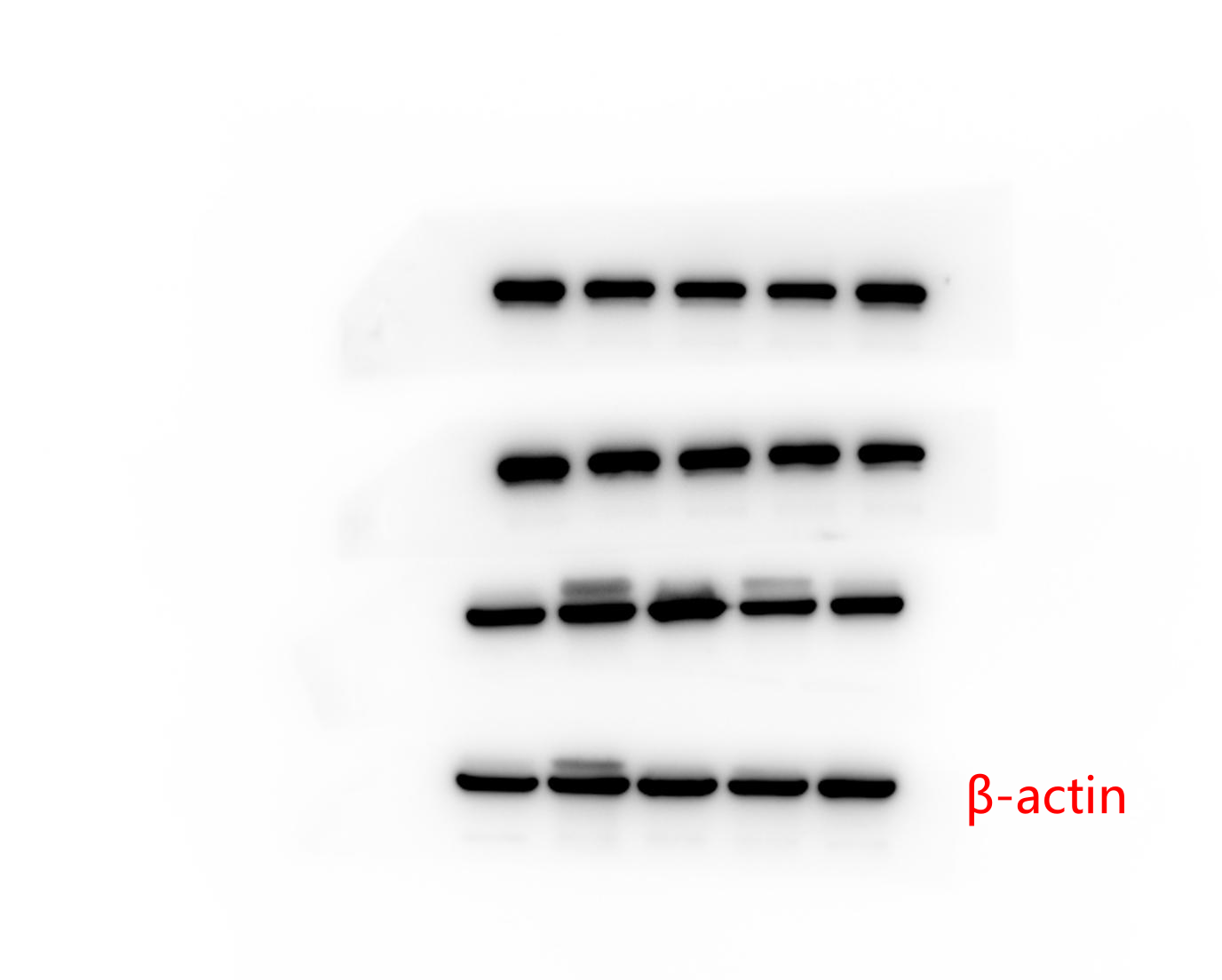

Supplement: Supplementary file 1 [file DataSheet1.ZIP › Supplementary Materials/supplementary materials ( original western blot figures)/Figure 1(original western blot figures)/β-actin(hippocampus of CORT model).tif]

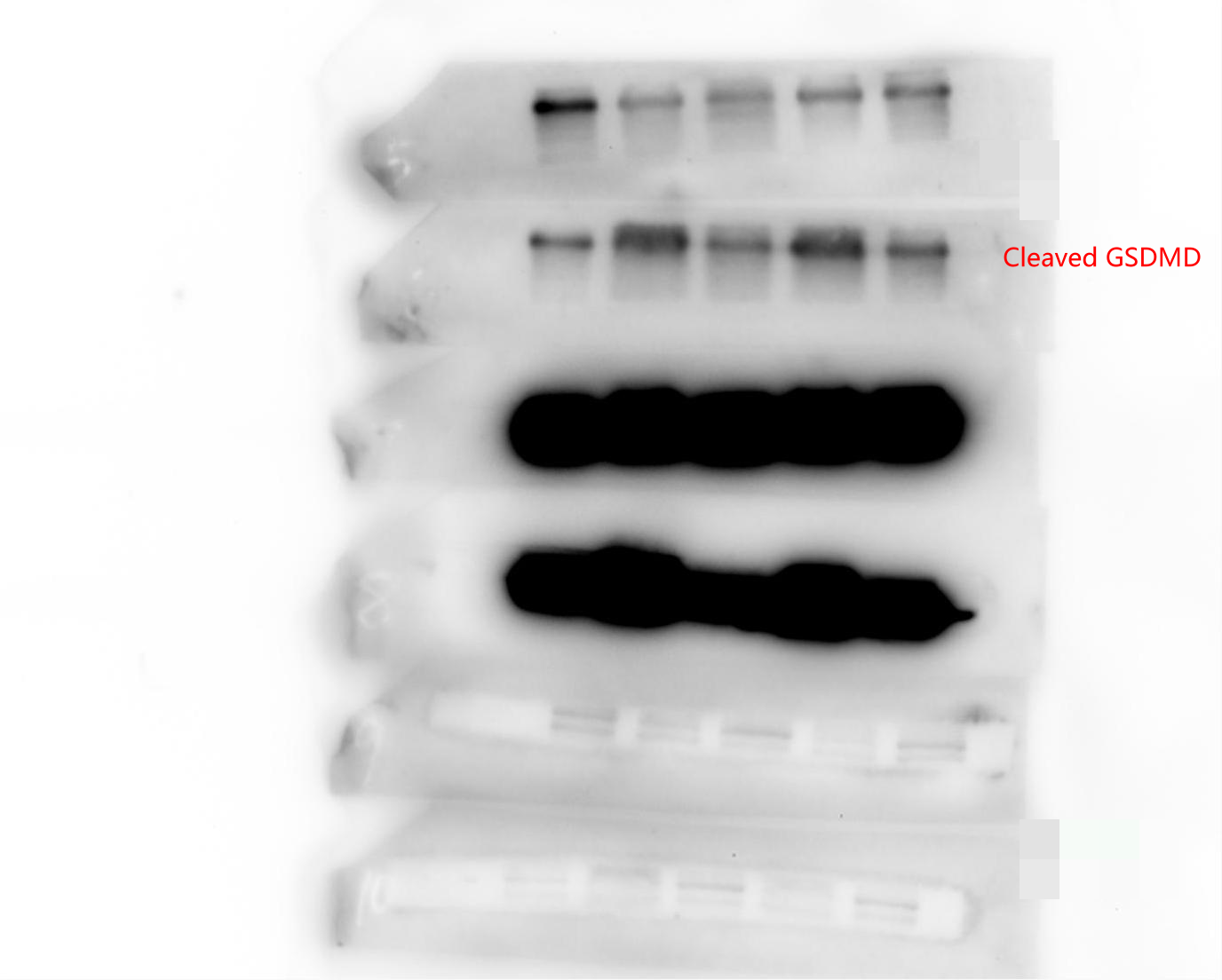

Supplement: Supplementary file 1 [file DataSheet1.ZIP › Supplementary Materials/supplementary materials ( original western blot figures)/Figure 2(original western blot figures)/Cleaved GSDMD(hippocampus of CORT model).tif]

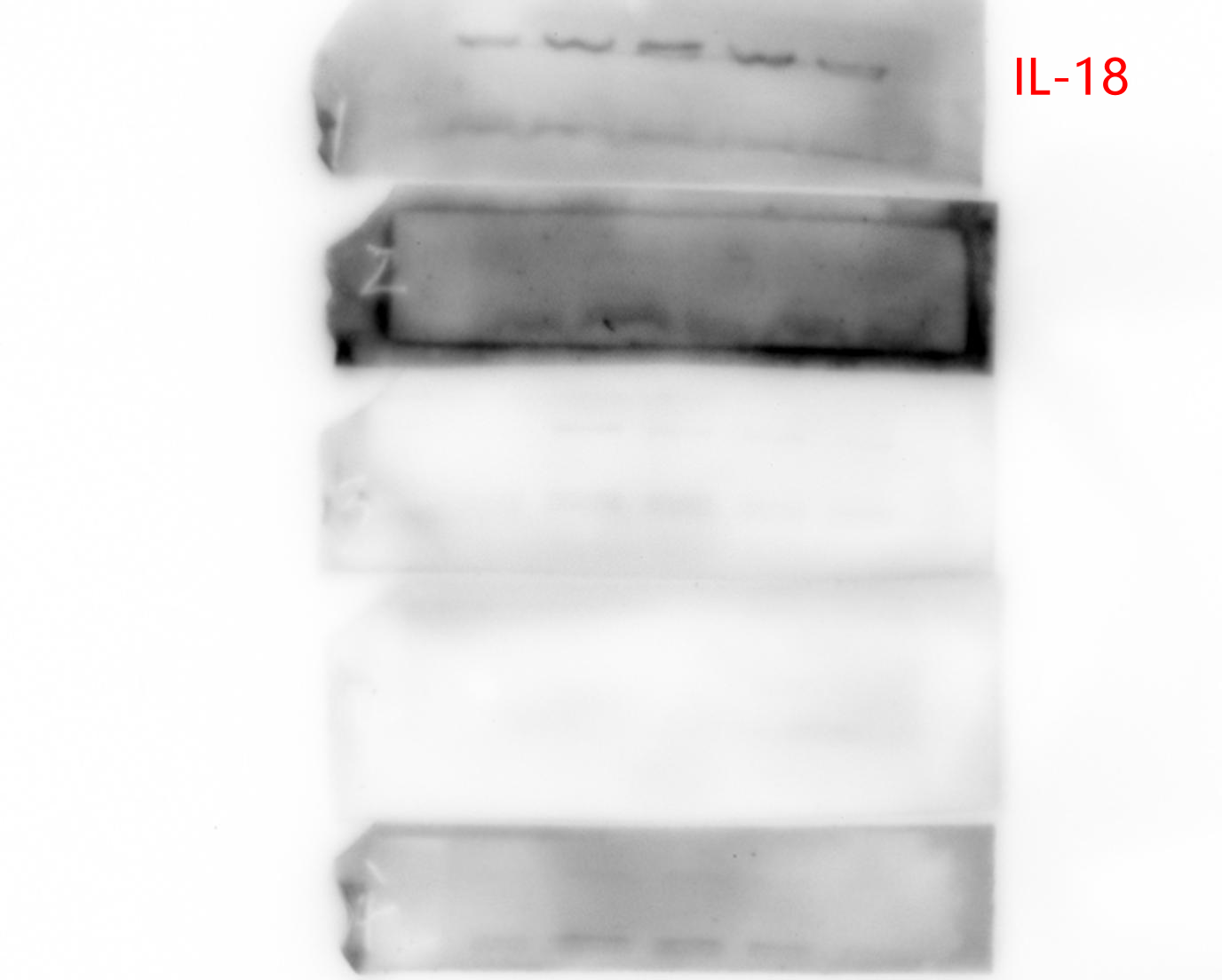

Supplement: Supplementary file 1 [file DataSheet1.ZIP › Supplementary Materials/supplementary materials ( original western blot figures)/Figure 2(original western blot figures)/IL-18 (hippocampus of CORT model).tif]

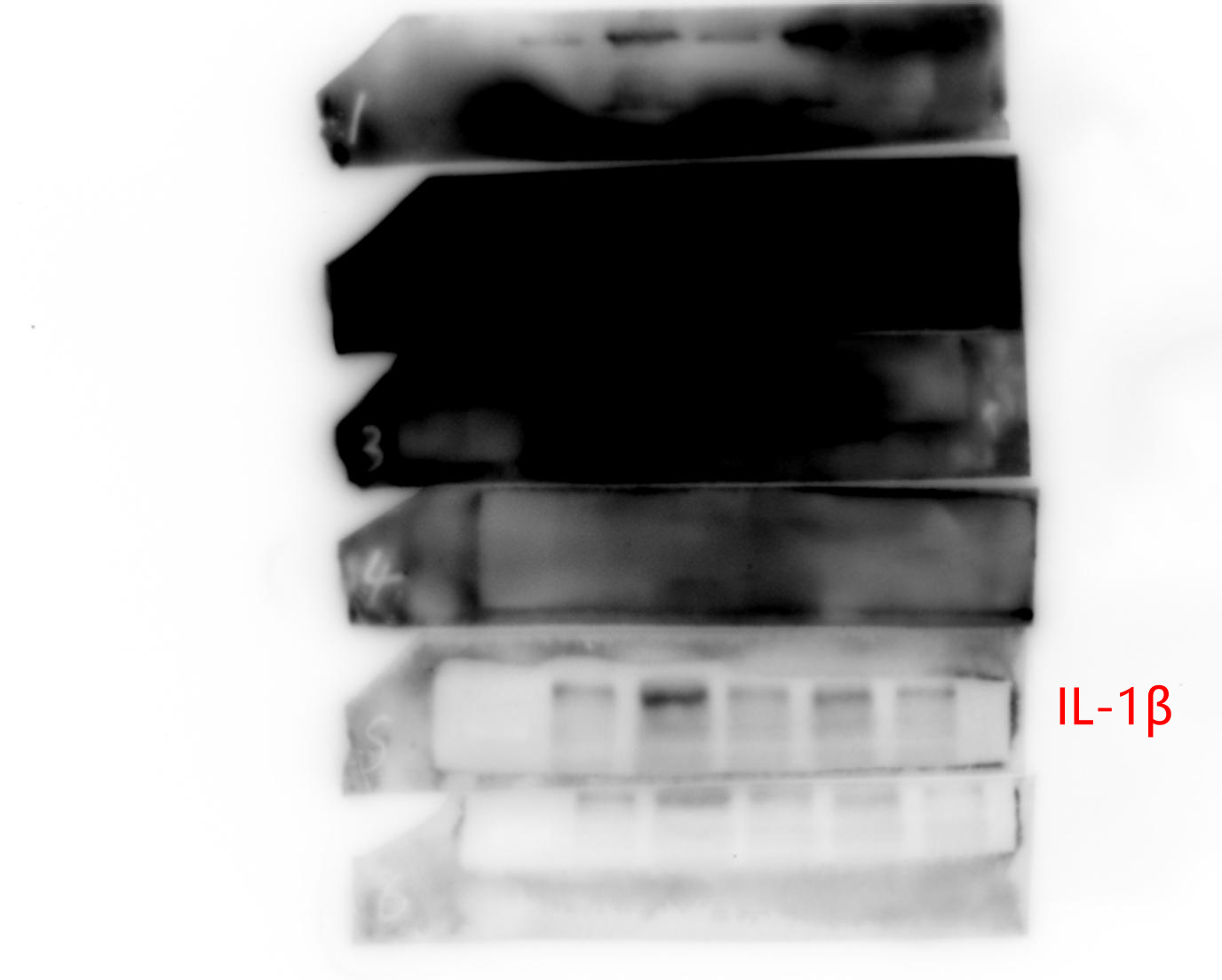

Supplement: Supplementary file 1 [file DataSheet1.ZIP › Supplementary Materials/supplementary materials ( original western blot figures)/Figure 2(original western blot figures)/IL-1β(hippocampus of CORT model).tif]

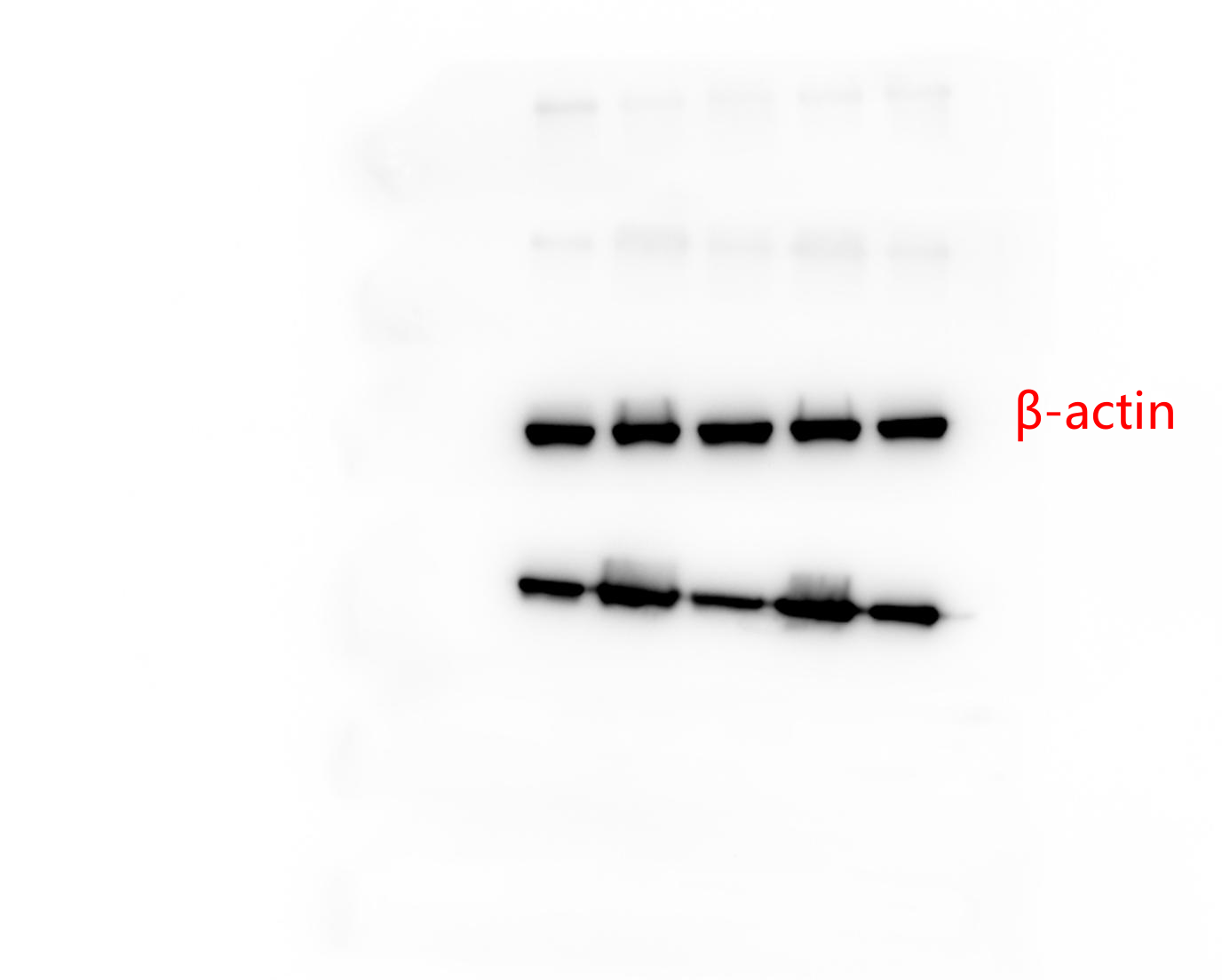

Supplement: Supplementary file 1 [file DataSheet1.ZIP › Supplementary Materials/supplementary materials ( original western blot figures)/Figure 2(original western blot figures)/β-actin (hippocampus of CORT model).tif]

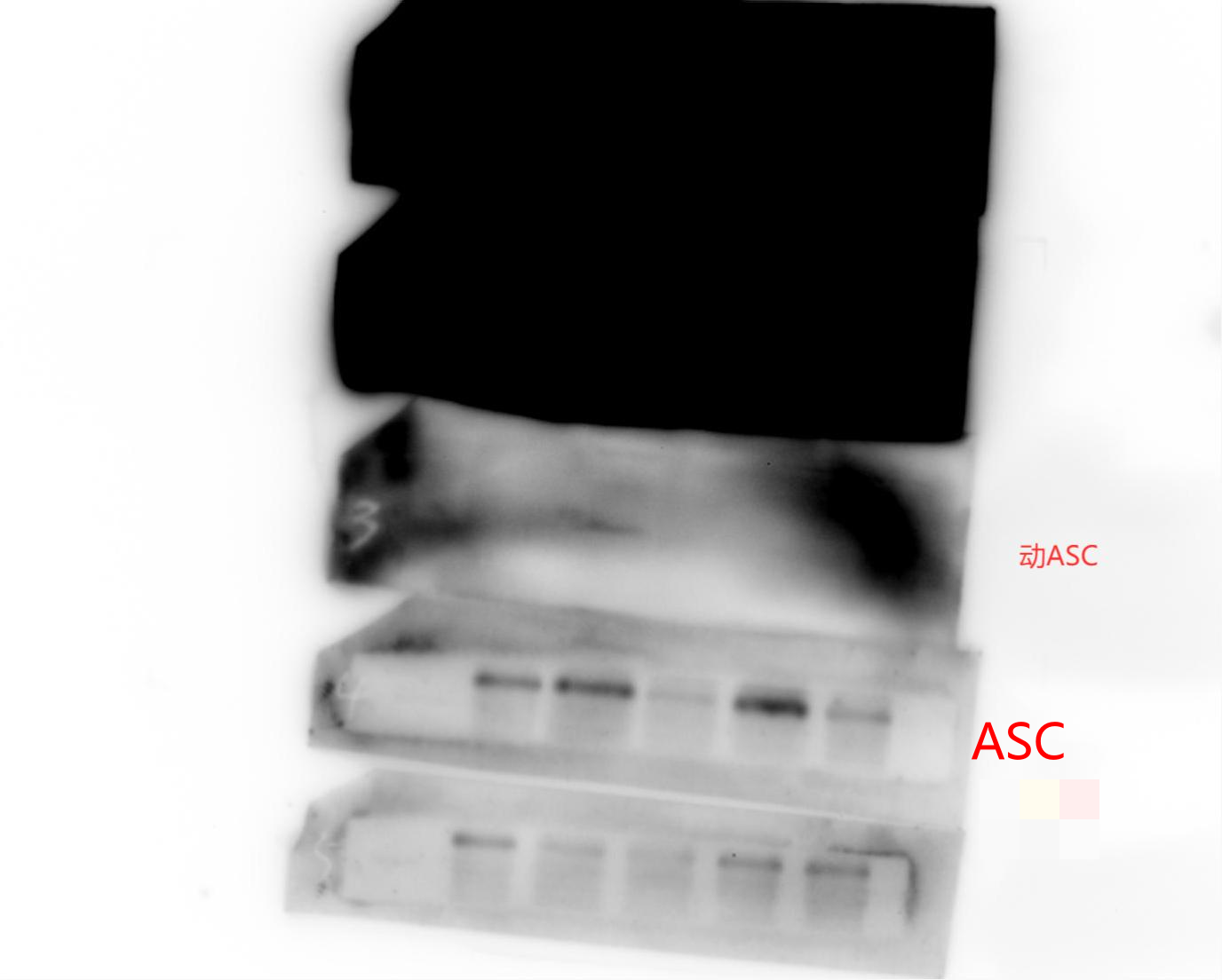

Supplement: Supplementary file 1 [file DataSheet1.ZIP › Supplementary Materials/supplementary materials ( original western blot figures)/Figure 3 (original western blot figures)/ASC(hippocampus of CORT model).tif]

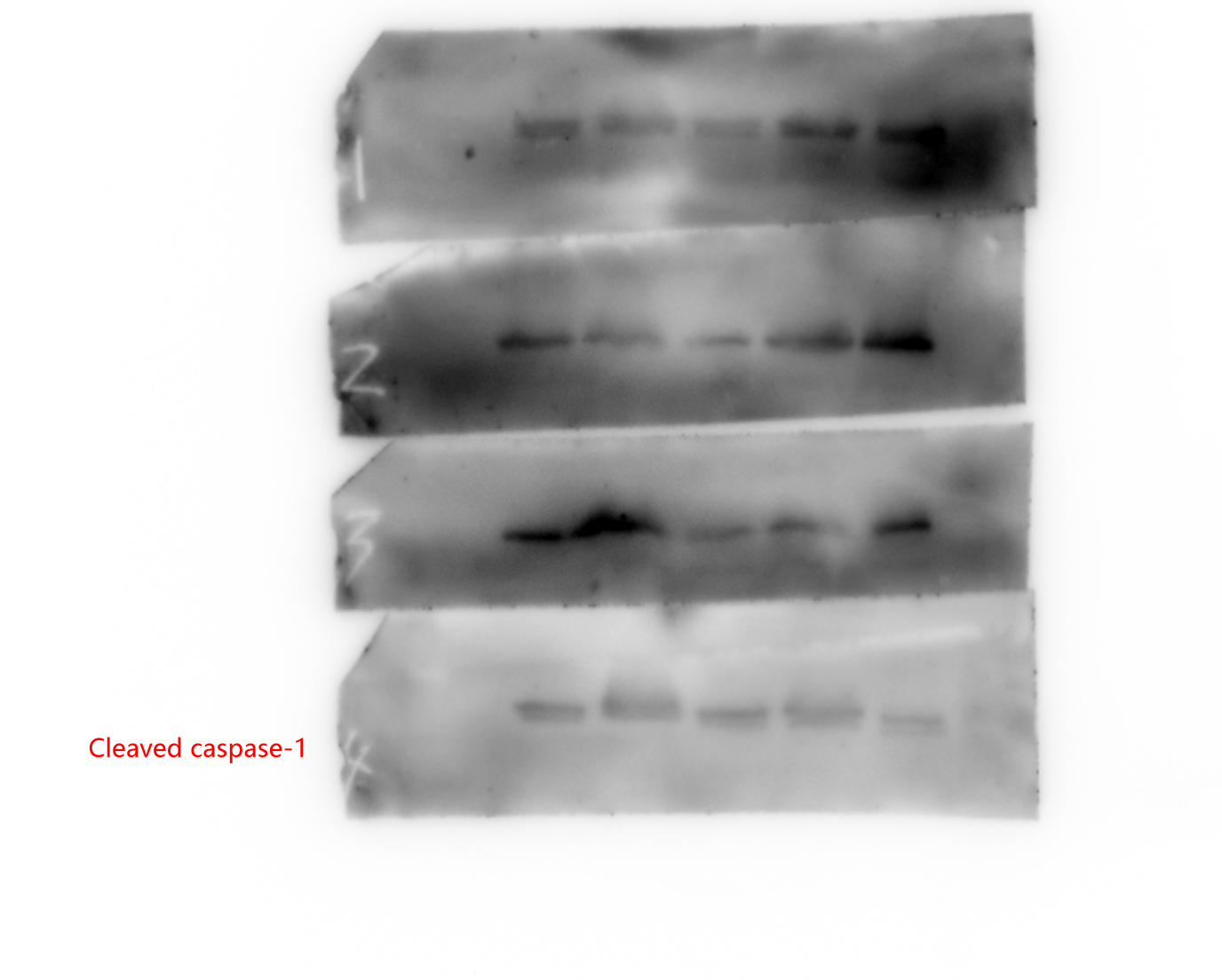

Supplement: Supplementary file 1 [file DataSheet1.ZIP › Supplementary Materials/supplementary materials ( original western blot figures)/Figure 3 (original western blot figures)/Cleaved caspase-1(hippocampus of CORT model).tif]

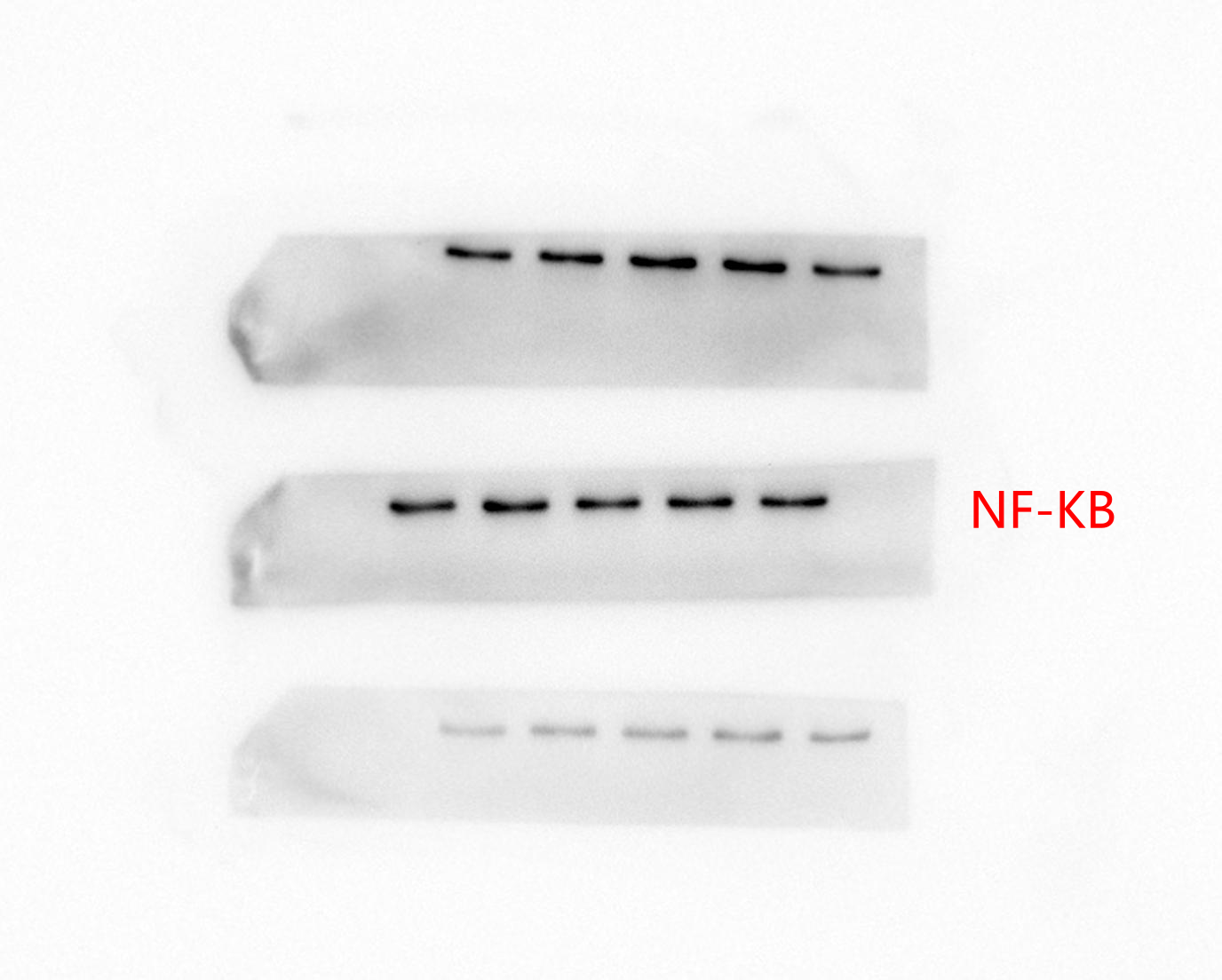

Supplement: Supplementary file 1 [file DataSheet1.ZIP › Supplementary Materials/supplementary materials ( original western blot figures)/Figure 3 (original western blot figures)/NF-κB(hippocampus of CORT model).tif]

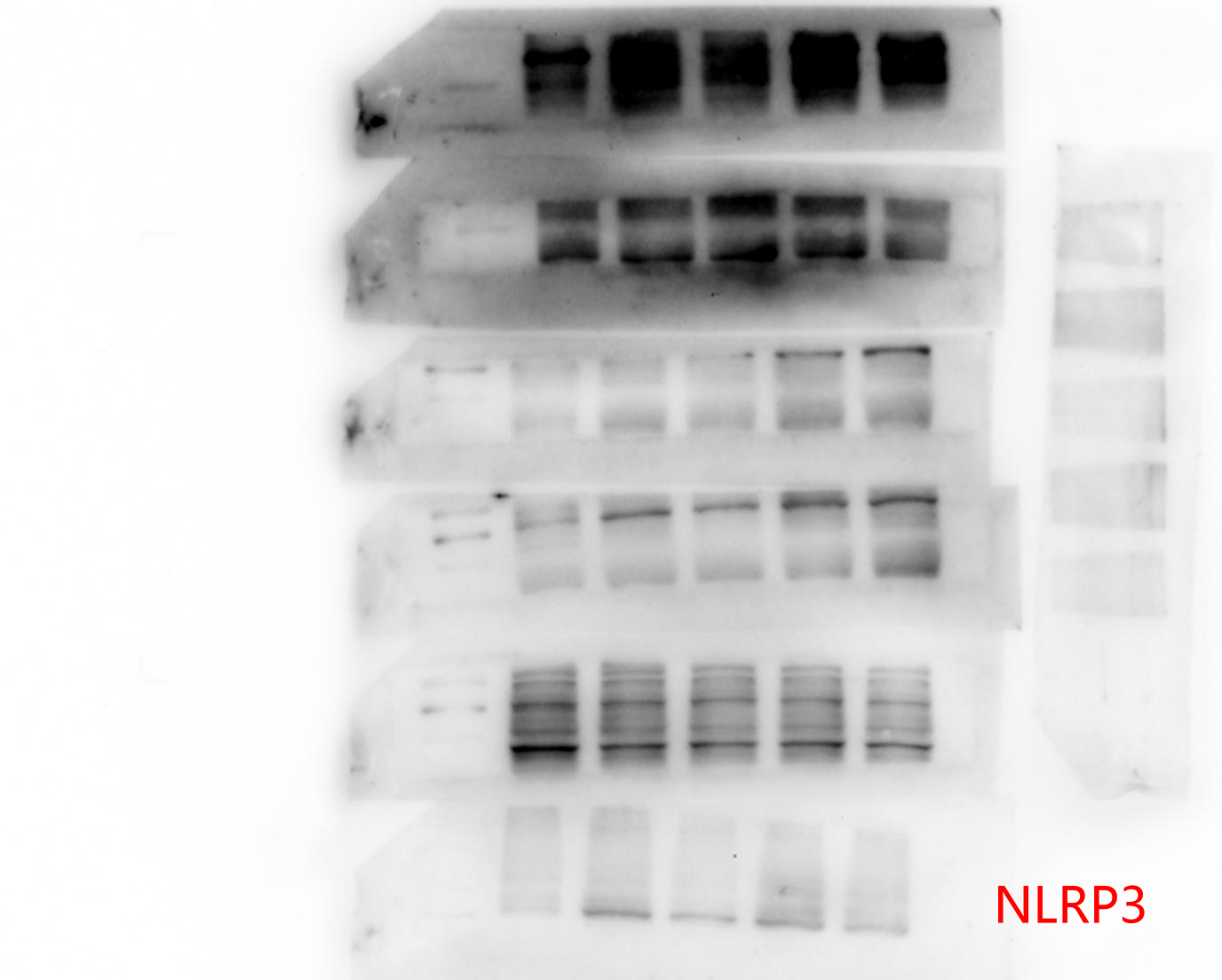

Supplement: Supplementary file 1 [file DataSheet1.ZIP › Supplementary Materials/supplementary materials ( original western blot figures)/Figure 3 (original western blot figures)/NLRP3 (hippocampus of CORT model).tif]

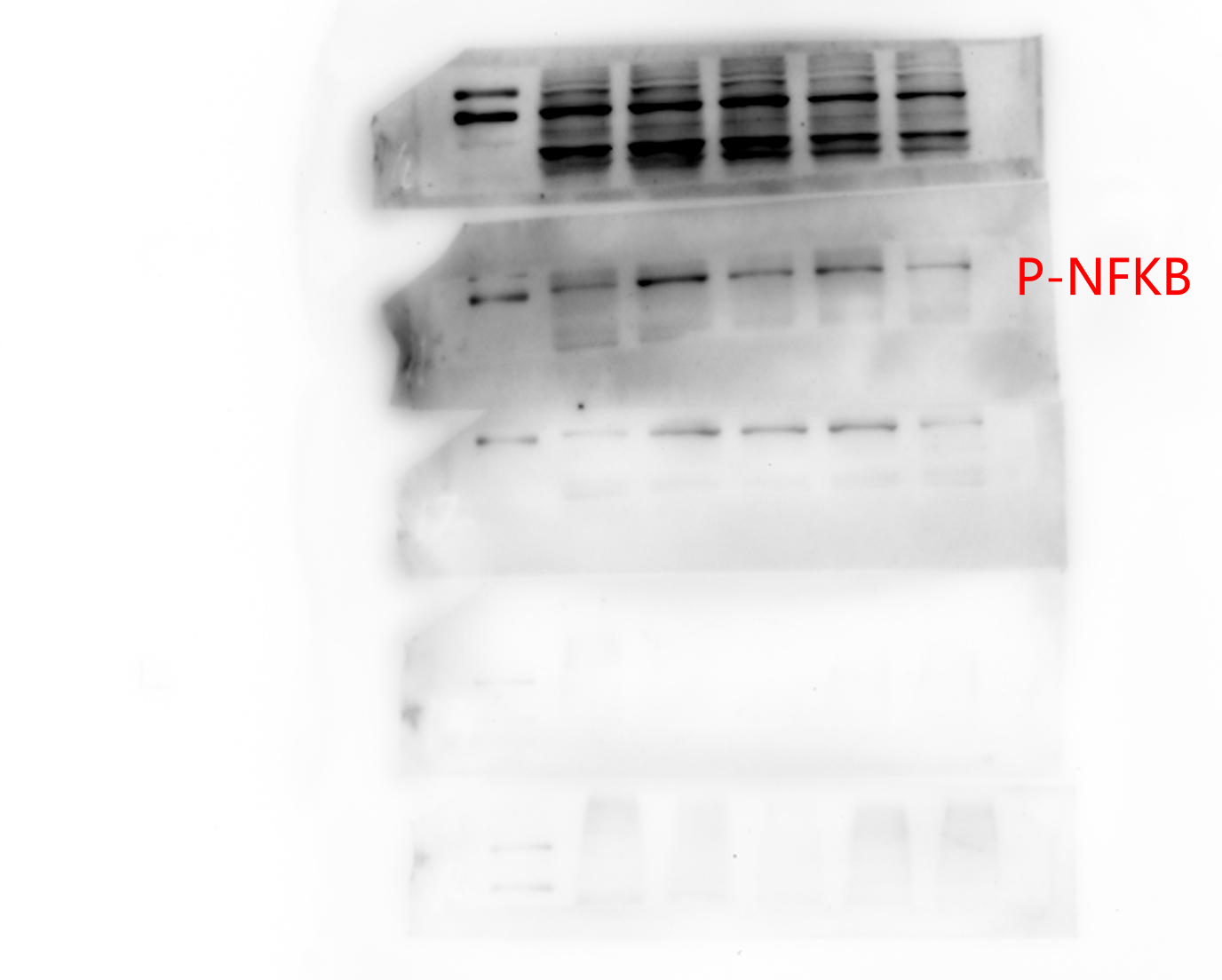

Supplement: Supplementary file 1 [file DataSheet1.ZIP › Supplementary Materials/supplementary materials ( original western blot figures)/Figure 3 (original western blot figures)/P-NF-κB (hippocampus of CORT model).tif]

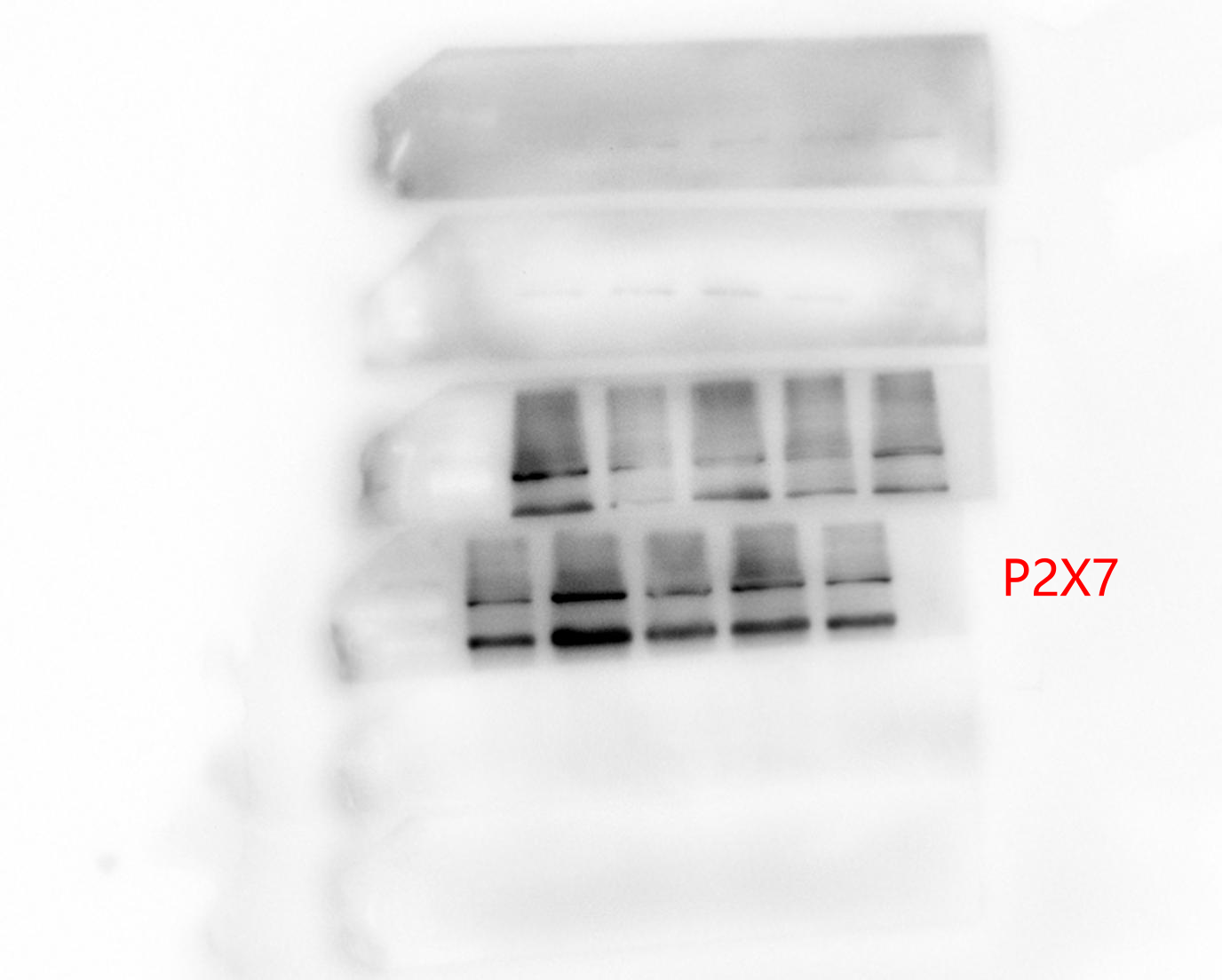

Supplement: Supplementary file 1 [file DataSheet1.ZIP › Supplementary Materials/supplementary materials ( original western blot figures)/Figure 3 (original western blot figures)/P2X7 (hippocampus of CORT model).tif]

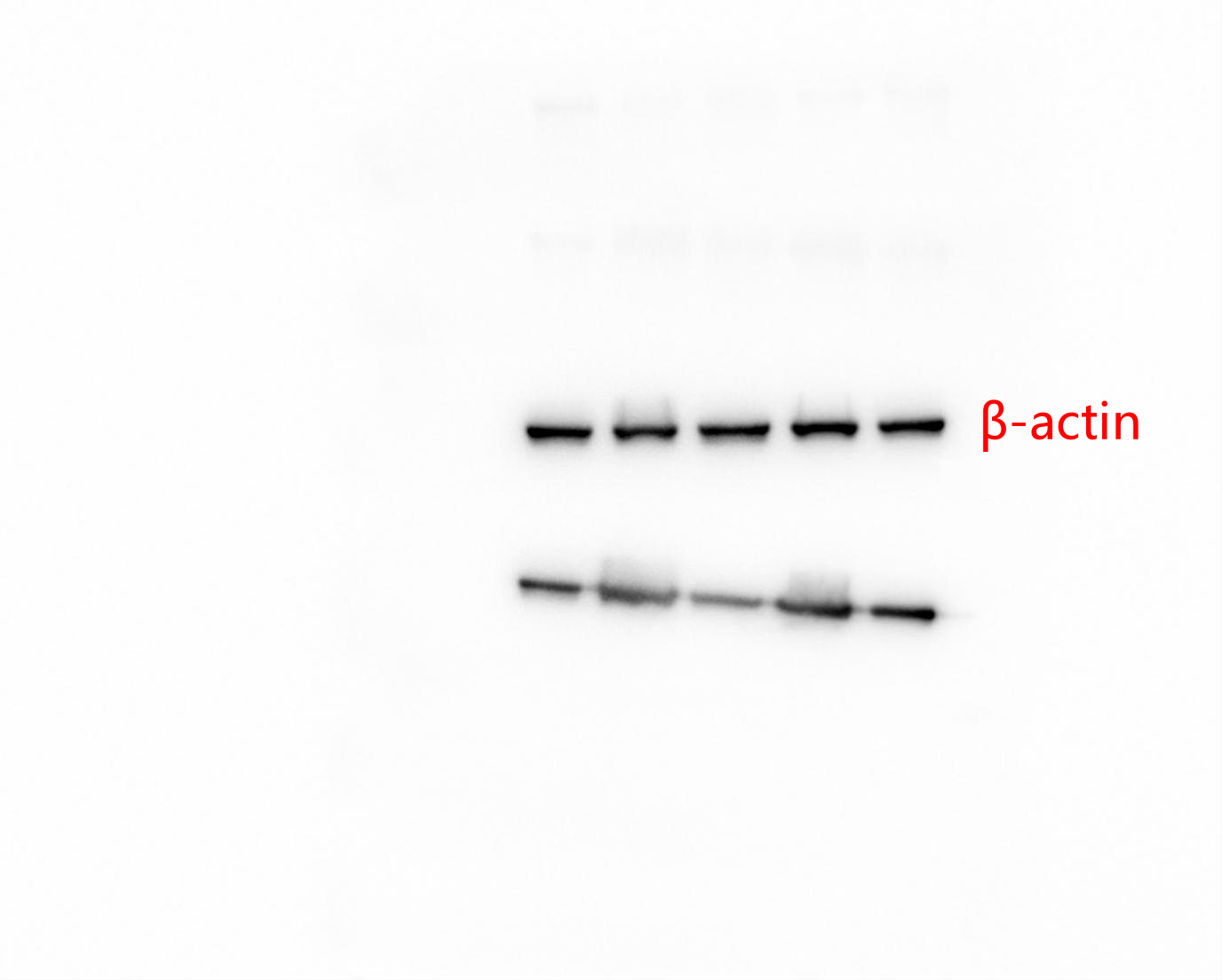

Supplement: Supplementary file 1 [file DataSheet1.ZIP › Supplementary Materials/supplementary materials ( original western blot figures)/Figure 3 (original western blot figures)/β-actin (hippocampus of CORT model).tif]

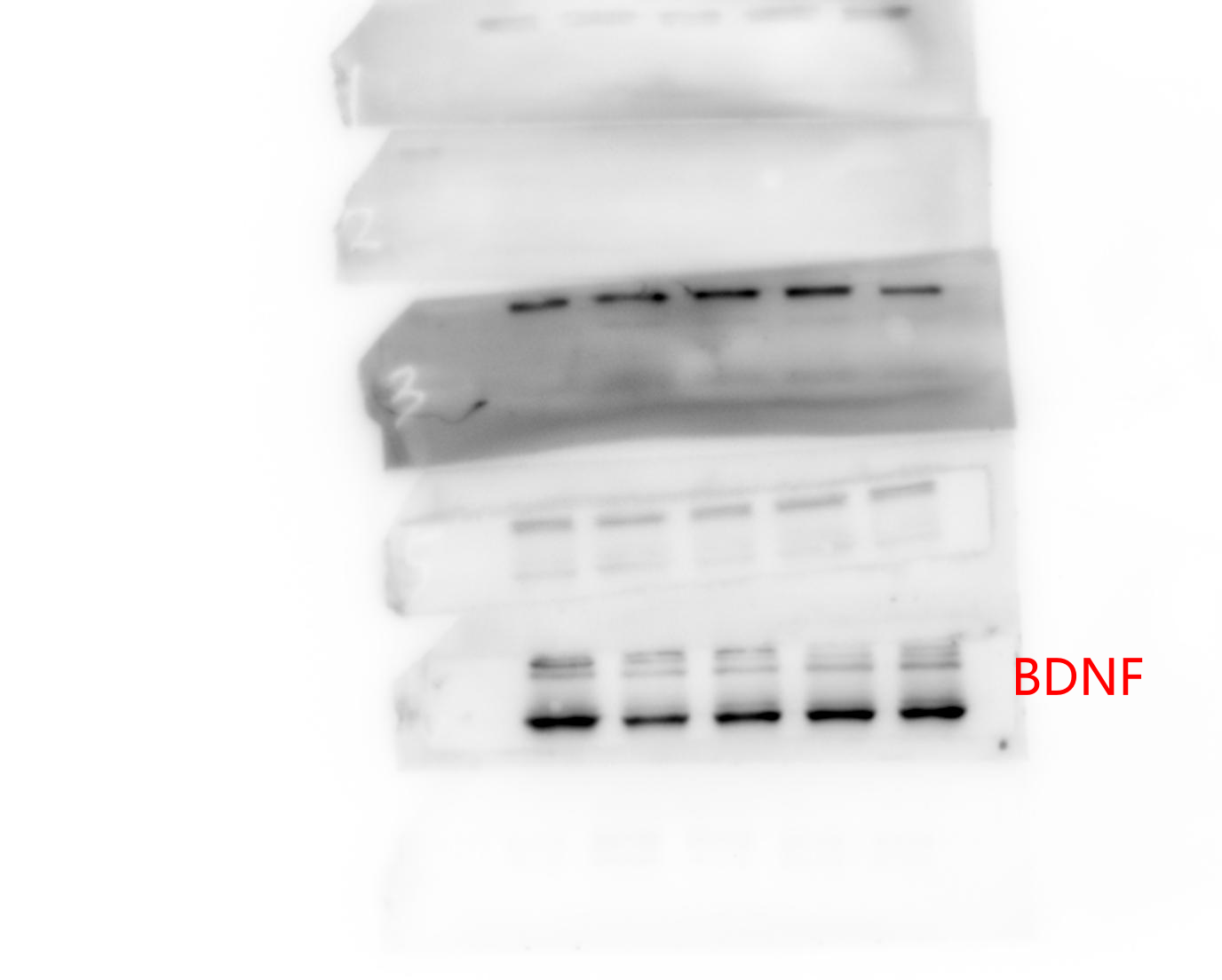

Supplement: Supplementary file 1 [file DataSheet1.ZIP › Supplementary Materials/supplementary materials ( original western blot figures)/Figure 4 (original western blot figures)/BDNF (hippocampus of LPS model).tif]

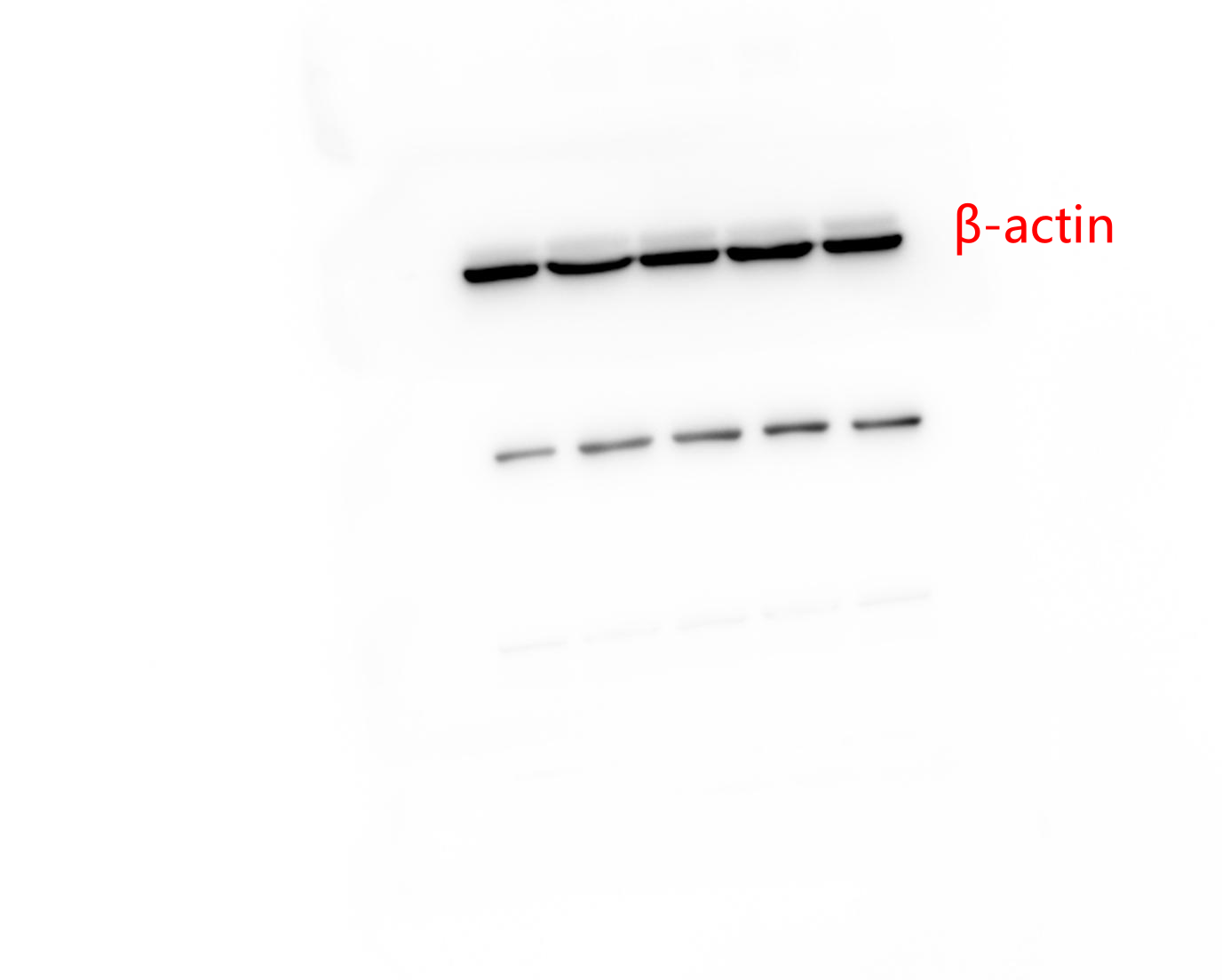

Supplement: Supplementary file 1 [file DataSheet1.ZIP › Supplementary Materials/supplementary materials ( original western blot figures)/Figure 4 (original western blot figures)/β-actin (hippocampus of LPS model).tif]

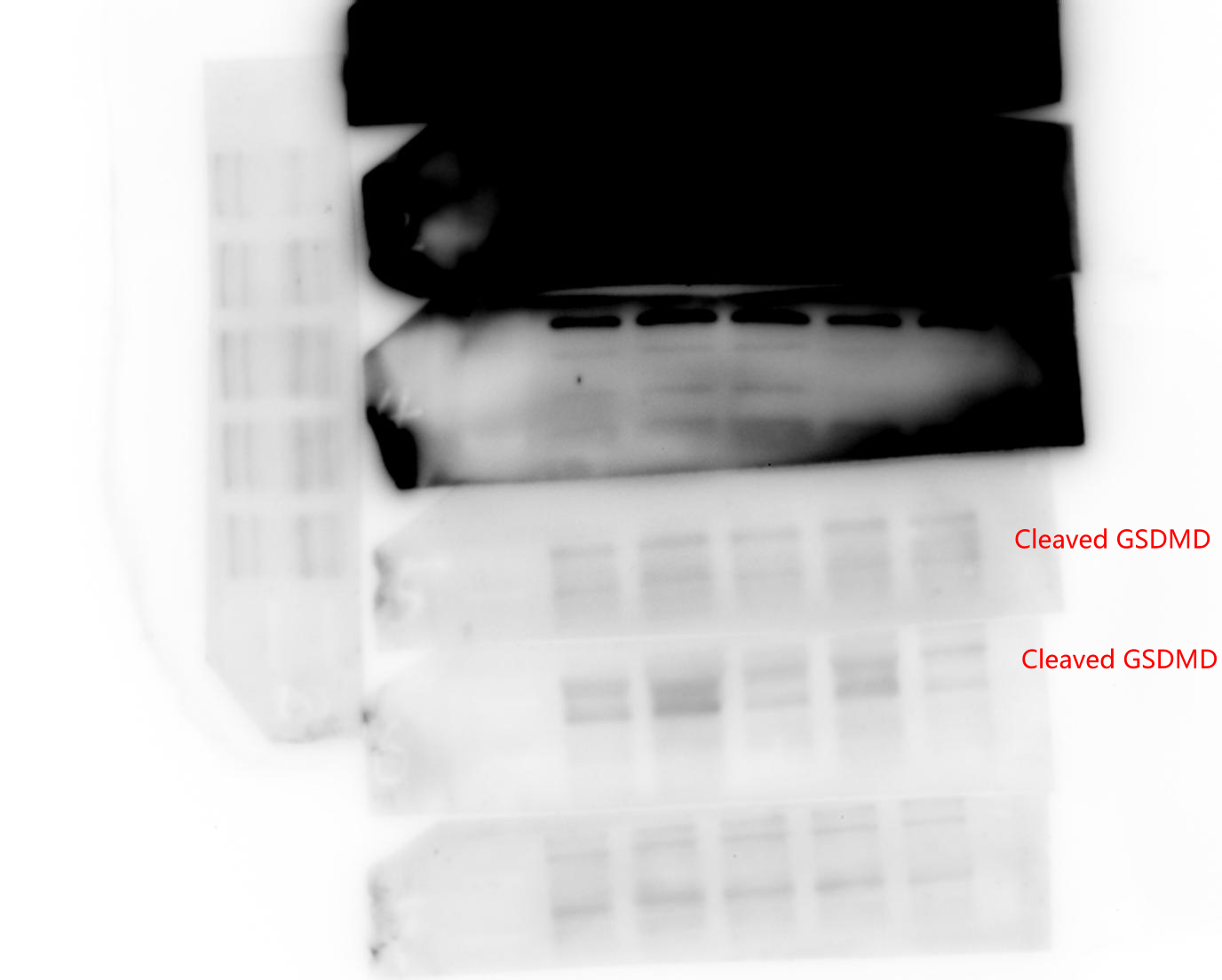

Supplement: Supplementary file 1 [file DataSheet1.ZIP › Supplementary Materials/supplementary materials ( original western blot figures)/Figure 5 (original western blot figures)/Cleaved GSDMD (hippocampus of LPS model).tif]

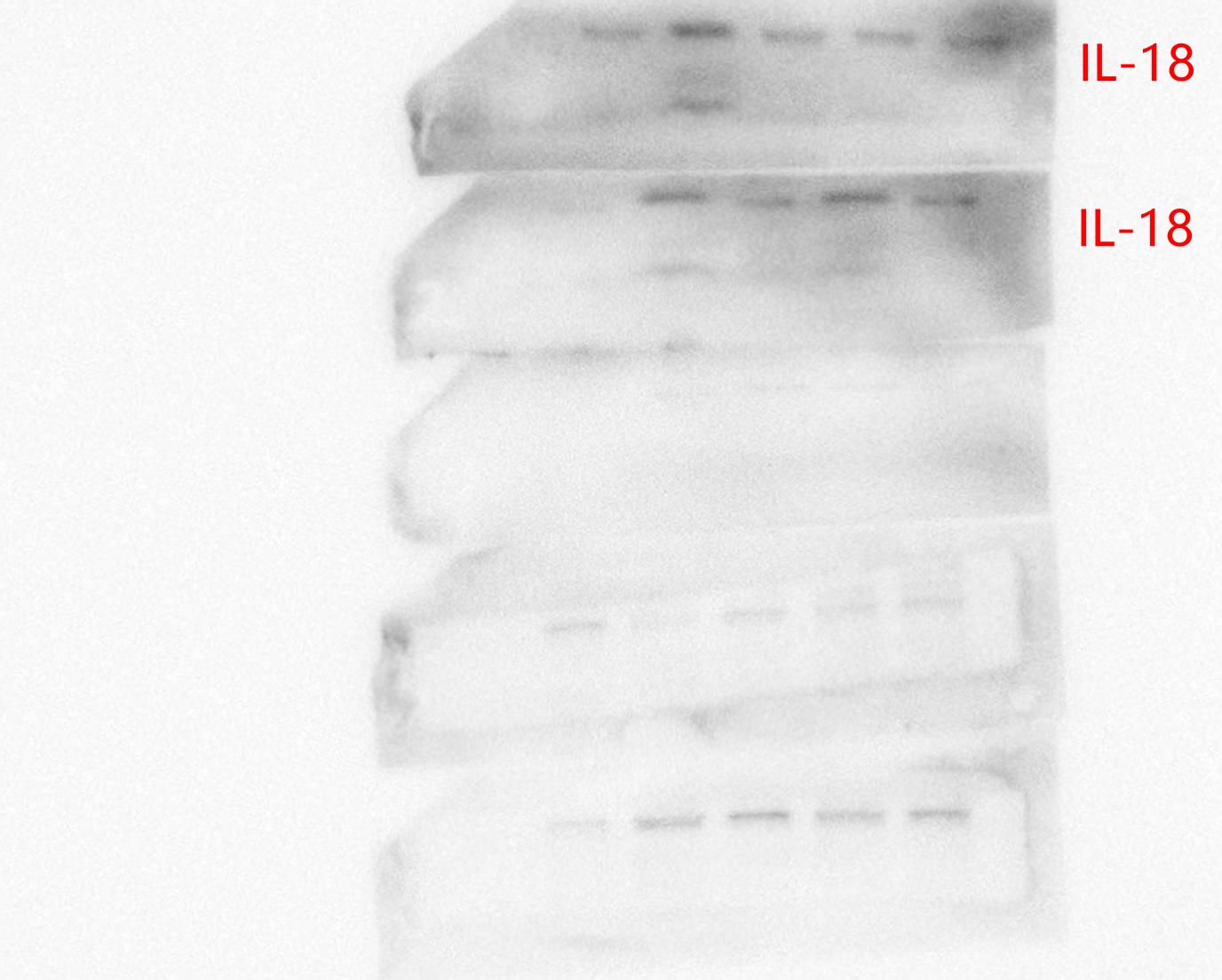

Supplement: Supplementary file 1 [file DataSheet1.ZIP › Supplementary Materials/supplementary materials ( original western blot figures)/Figure 5 (original western blot figures)/IL-18 (hippocampus of LPS model).tif]

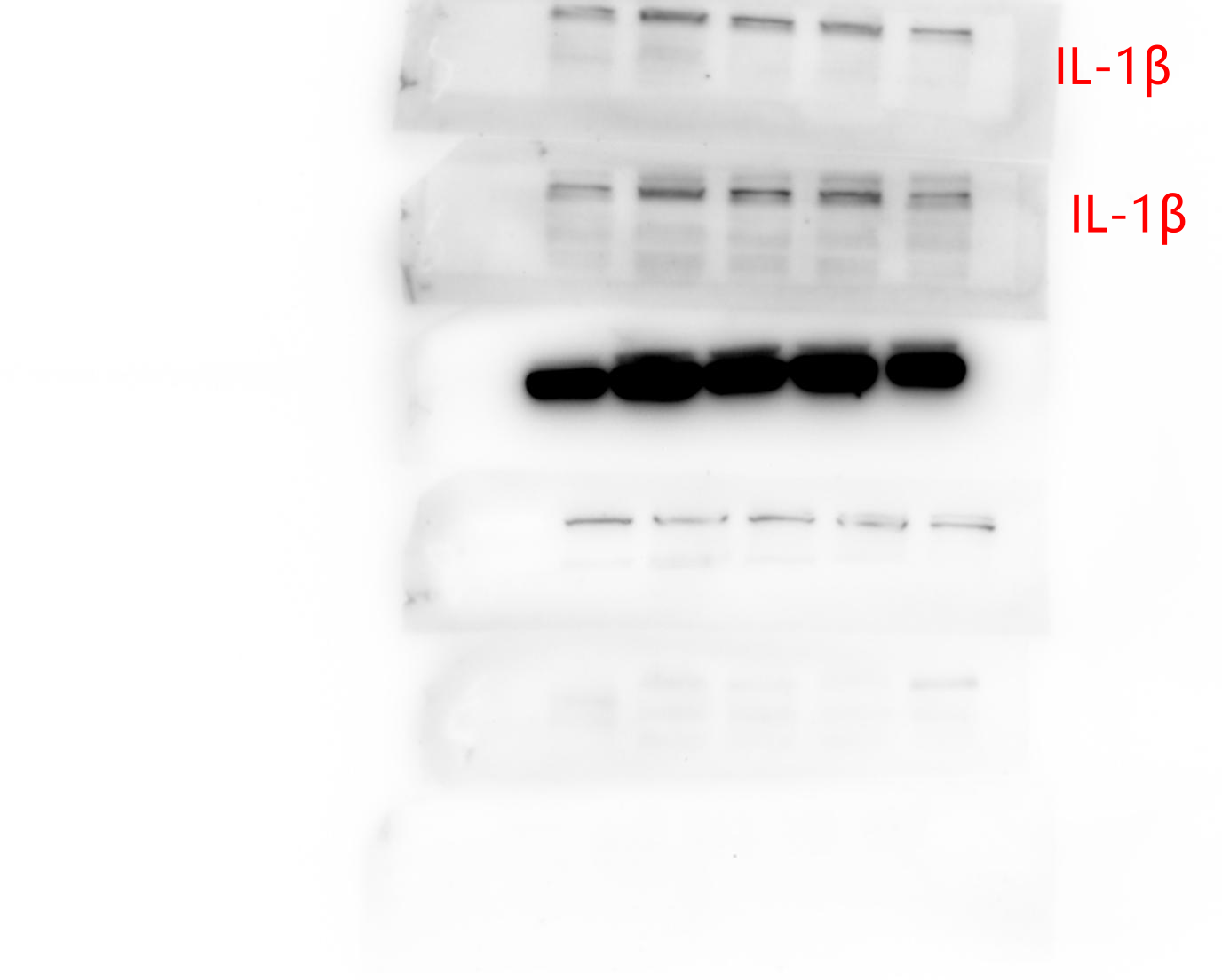

Supplement: Supplementary file 1 [file DataSheet1.ZIP › Supplementary Materials/supplementary materials ( original western blot figures)/Figure 5 (original western blot figures)/IL-1β (hippocampus of LPS model).tif]

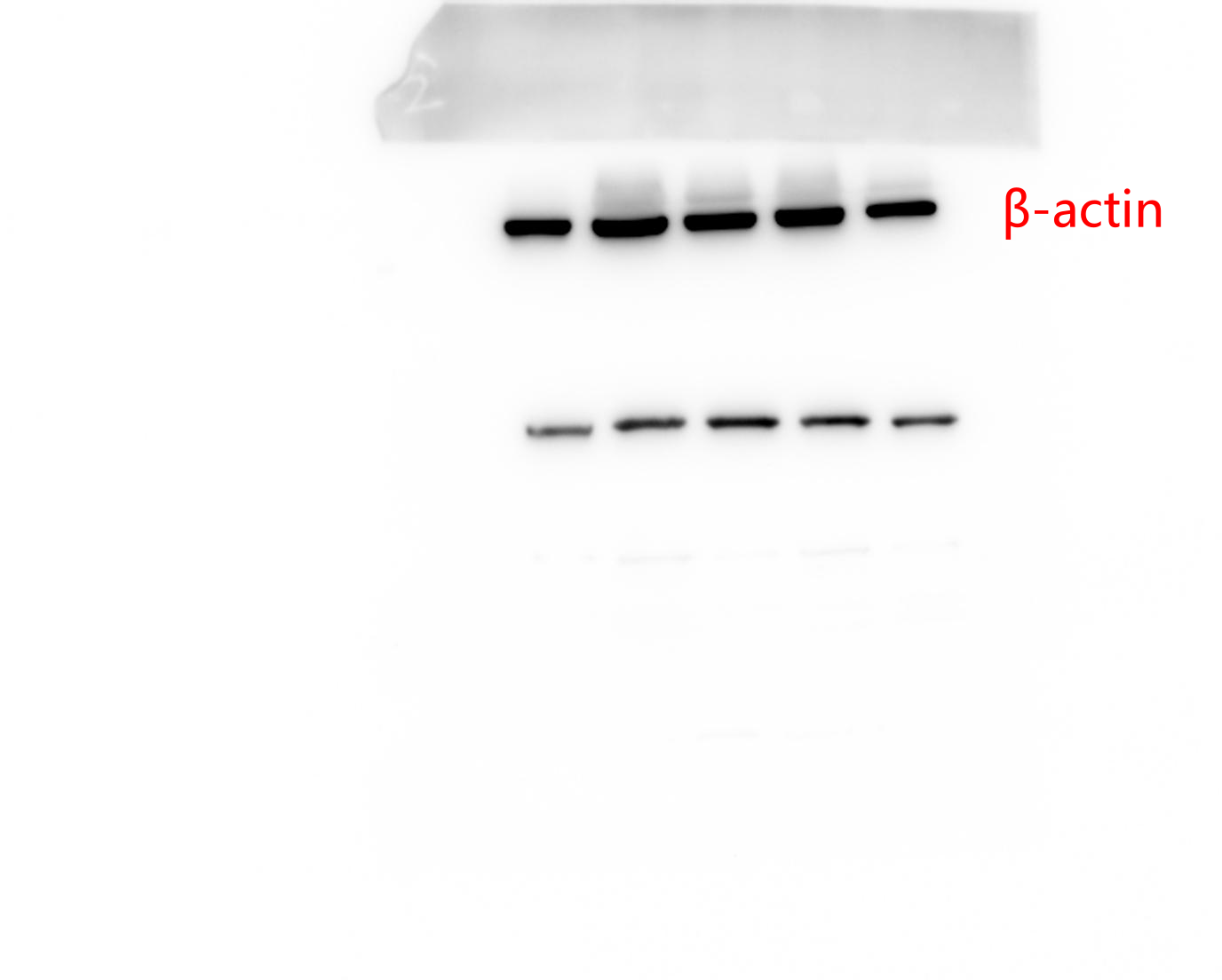

Supplement: Supplementary file 1 [file DataSheet1.ZIP › Supplementary Materials/supplementary materials ( original western blot figures)/Figure 5 (original western blot figures)/β-actin (hippocampus of LPS model).tif]

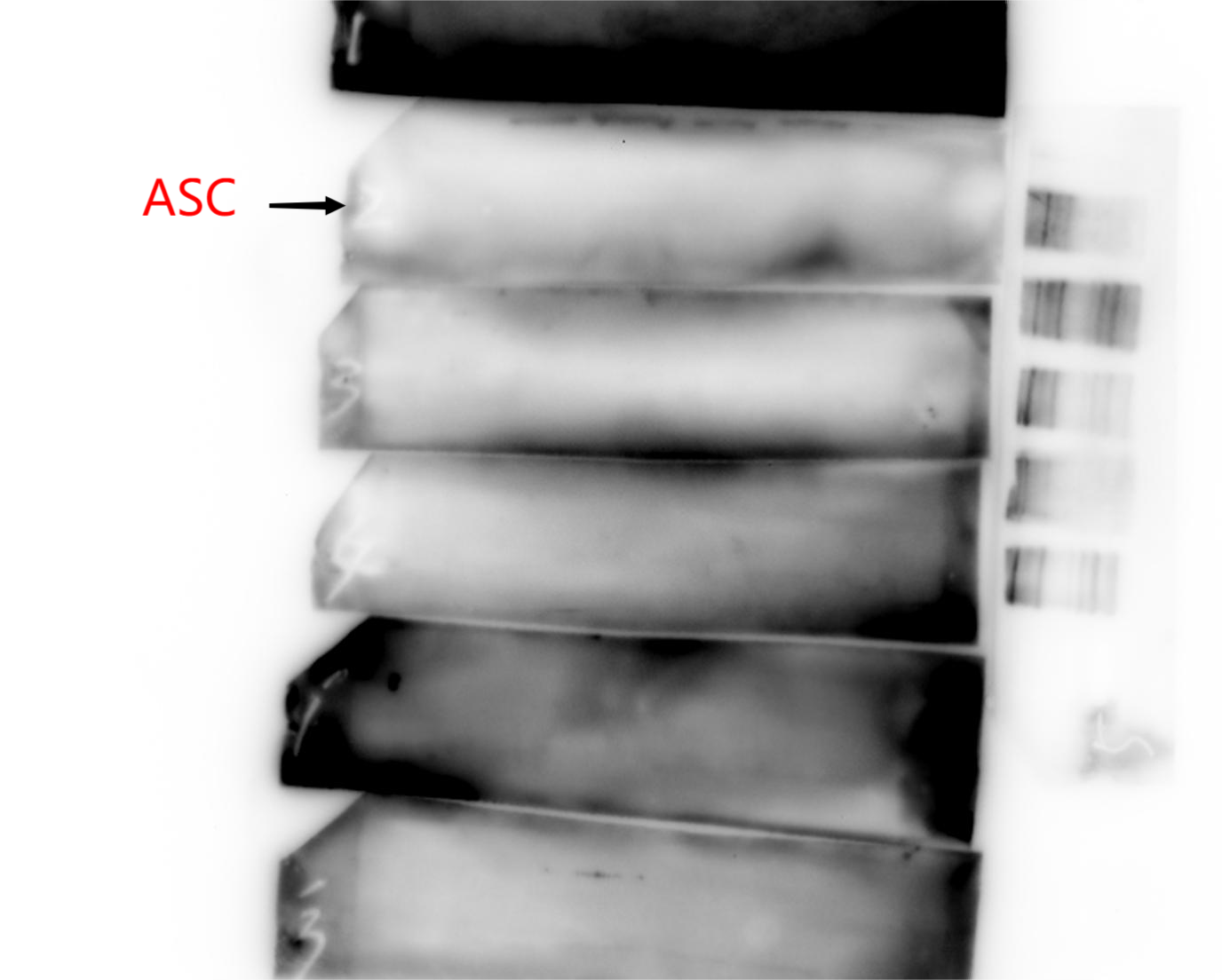

Supplement: Supplementary file 1 [file DataSheet1.ZIP › Supplementary Materials/supplementary materials ( original western blot figures)/Figure 6 (original western blot figures)/ASC (hippocampus of LPS model).png]

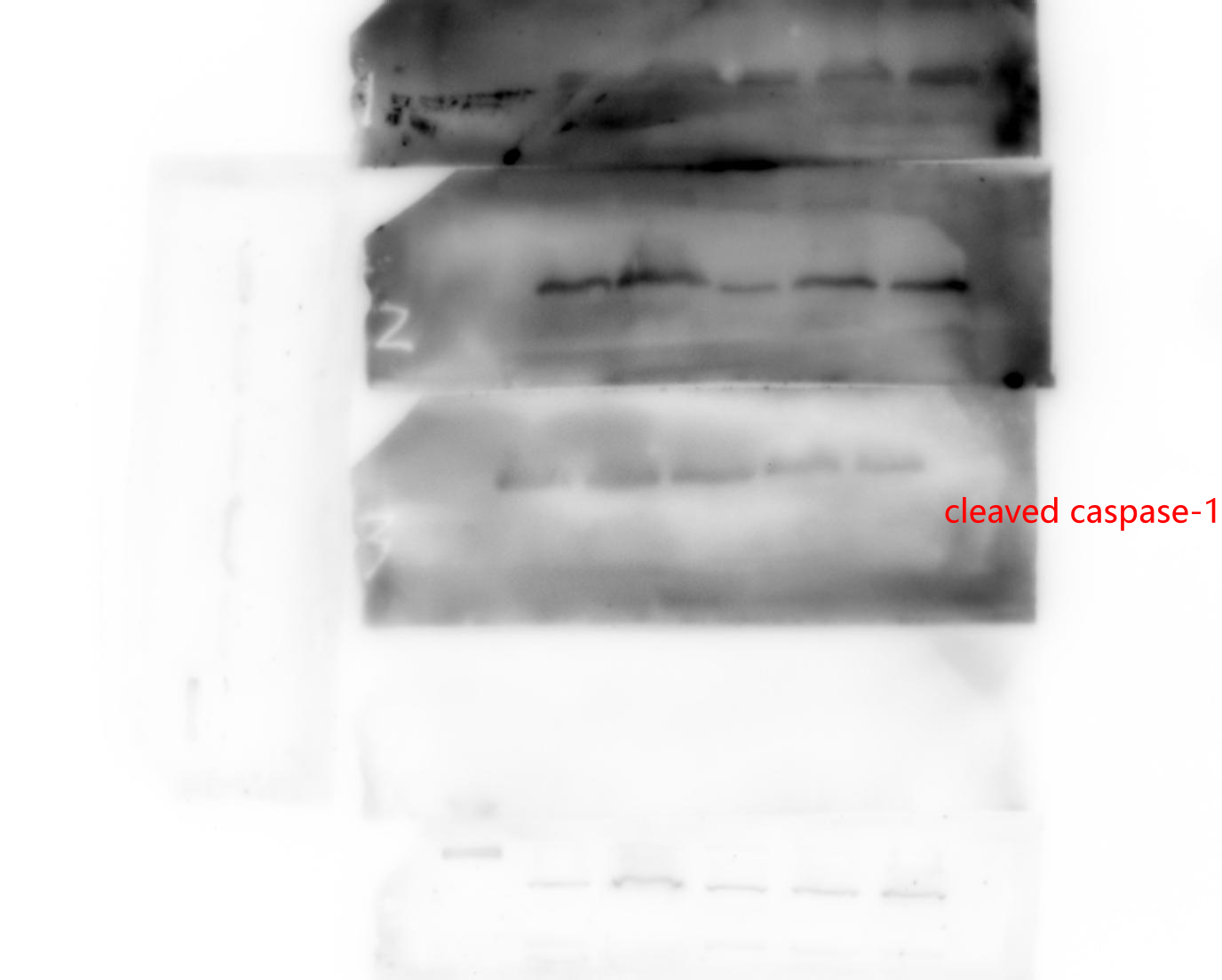

Supplement: Supplementary file 1 [file DataSheet1.ZIP › Supplementary Materials/supplementary materials ( original western blot figures)/Figure 6 (original western blot figures)/Cleaved caspase-1 (hippocampus of LPS model).tif]

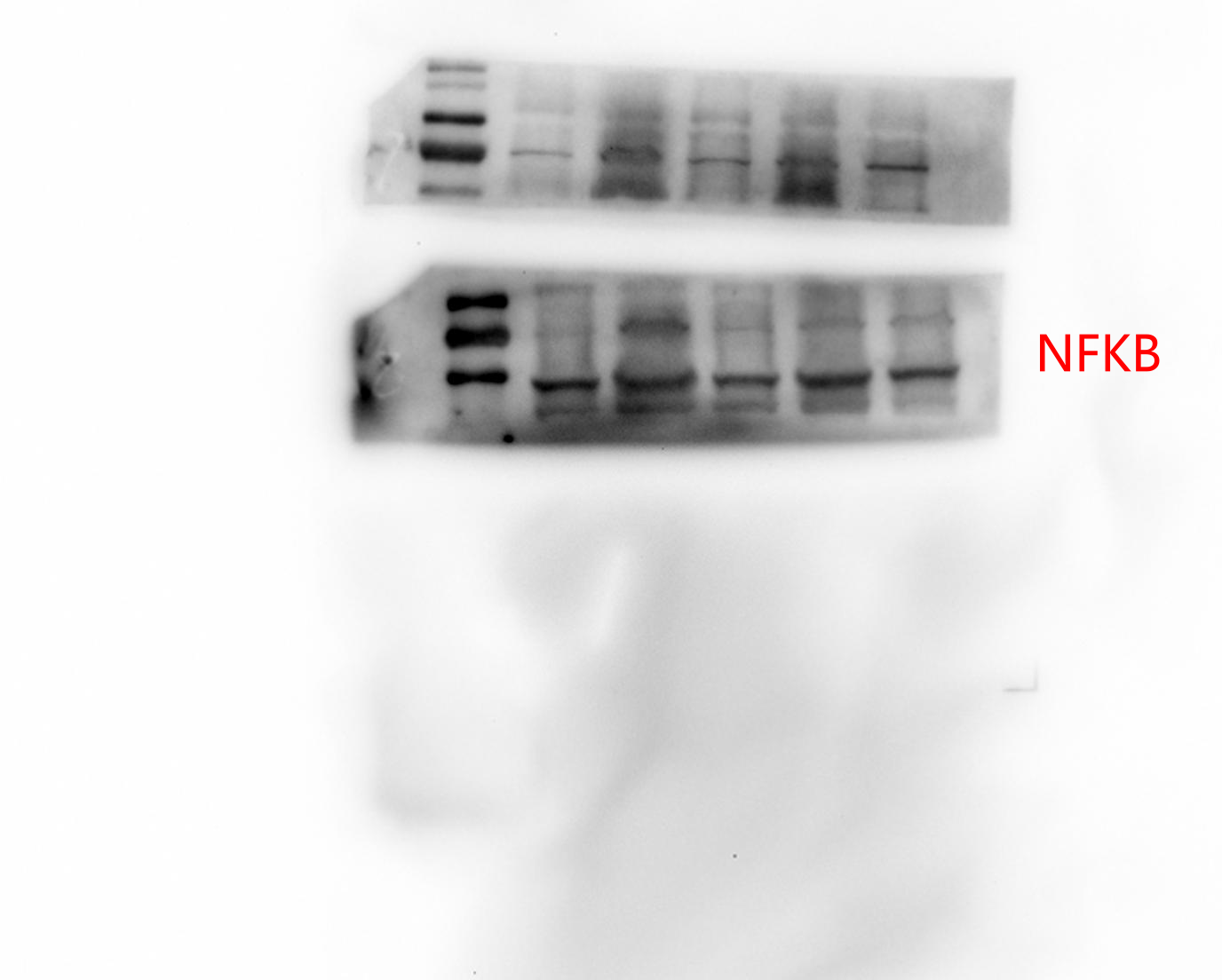

Supplement: Supplementary file 1 [file DataSheet1.ZIP › Supplementary Materials/supplementary materials ( original western blot figures)/Figure 6 (original western blot figures)/NF-κB (hippocampus of LPS model).tif]

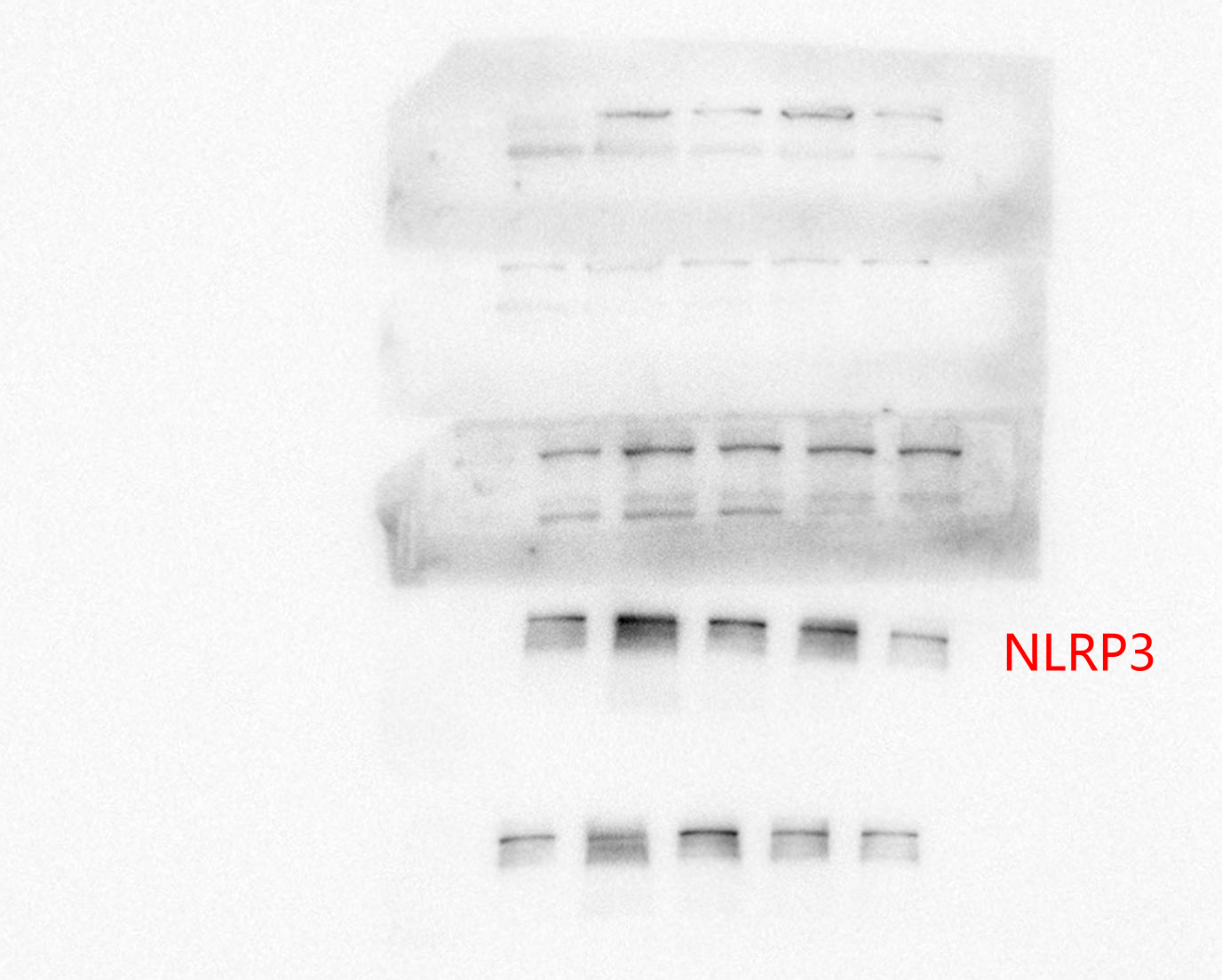

Supplement: Supplementary file 1 [file DataSheet1.ZIP › Supplementary Materials/supplementary materials ( original western blot figures)/Figure 6 (original western blot figures)/NLRP3 (hippocampus of LPS model).tif]

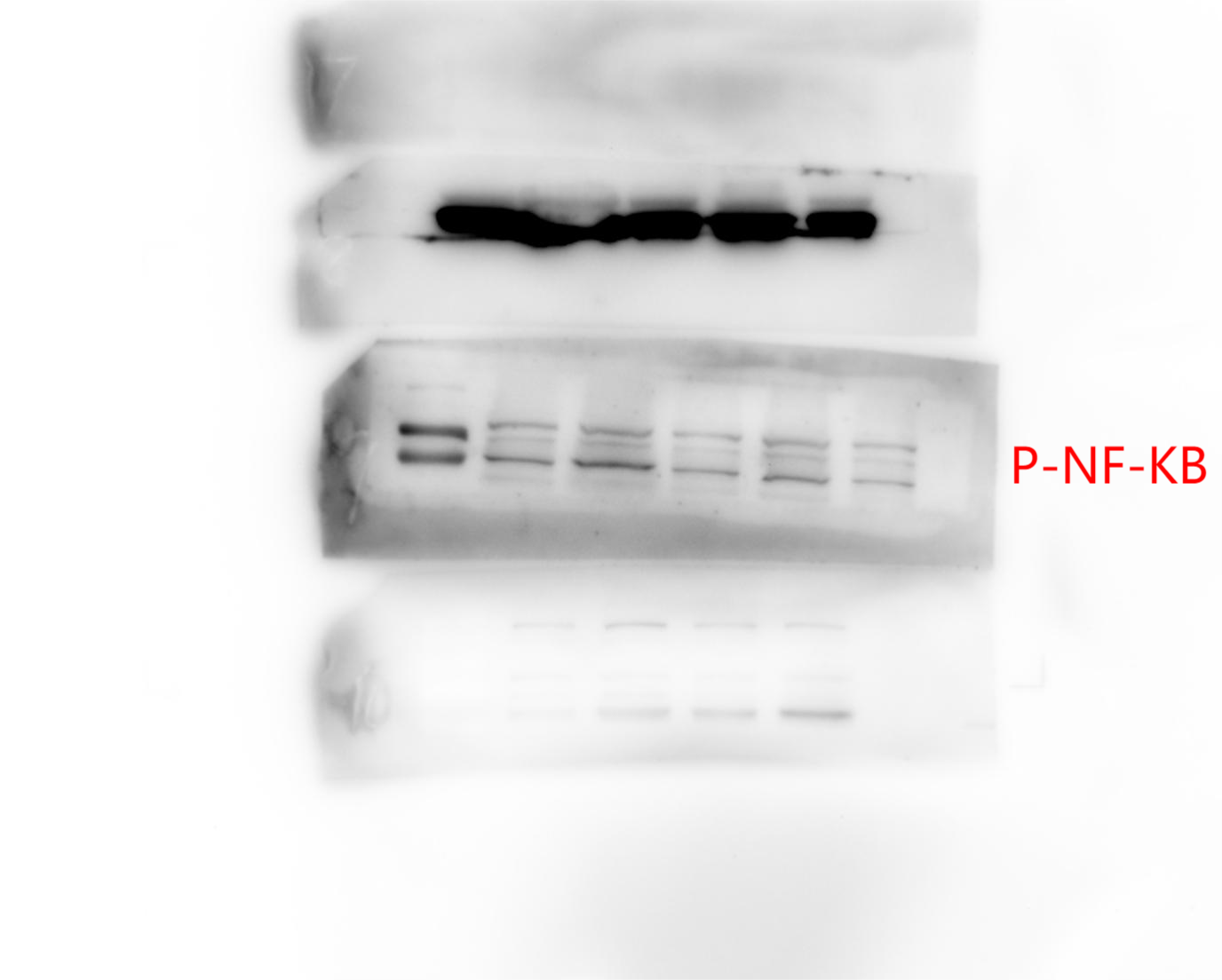

Supplement: Supplementary file 1 [file DataSheet1.ZIP › Supplementary Materials/supplementary materials ( original western blot figures)/Figure 6 (original western blot figures)/P-NF-κB (hippocampus of LPS model).png]

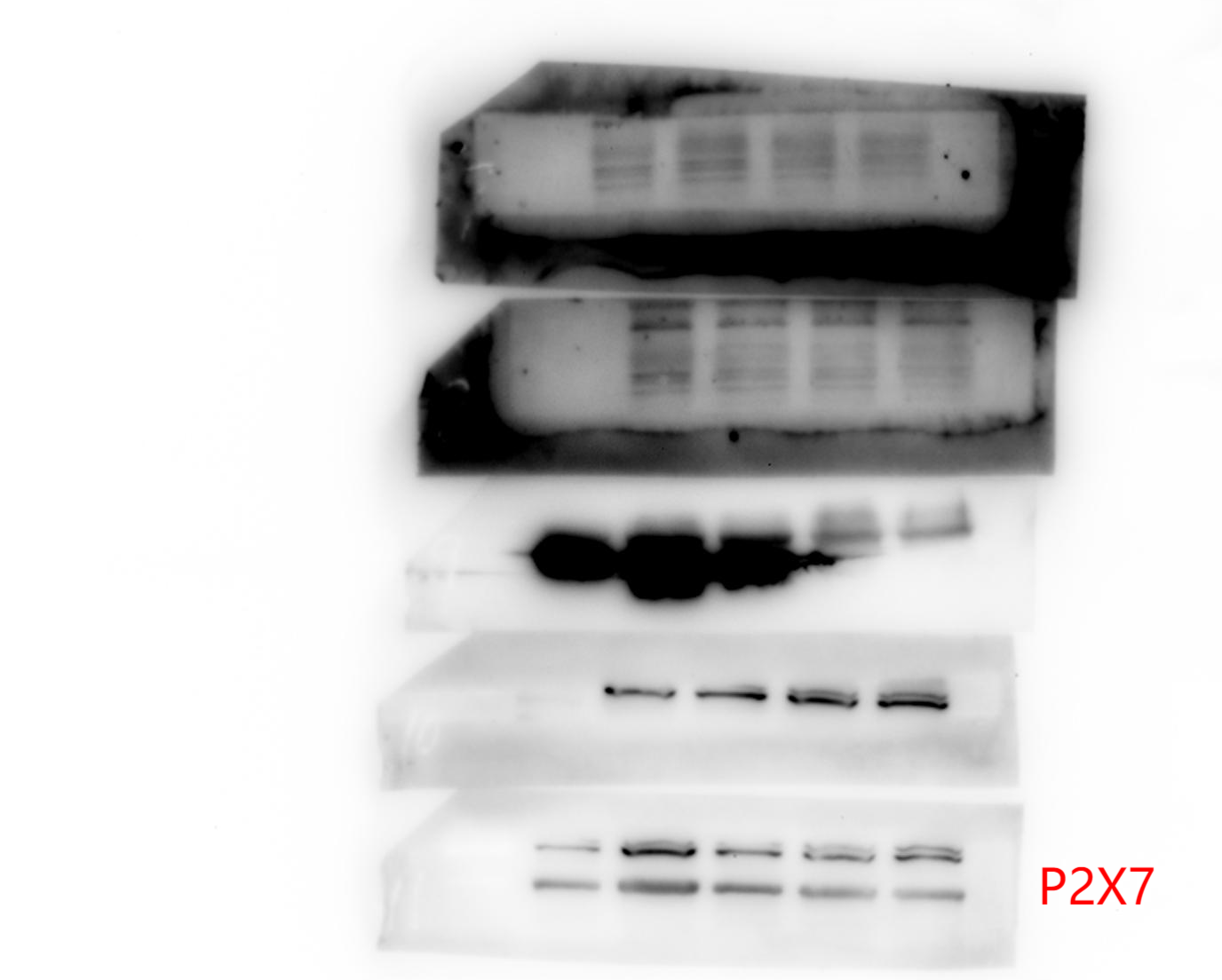

Supplement: Supplementary file 1 [file DataSheet1.ZIP › Supplementary Materials/supplementary materials ( original western blot figures)/Figure 6 (original western blot figures)/P2X7 (hippocampus of LPS model).png]

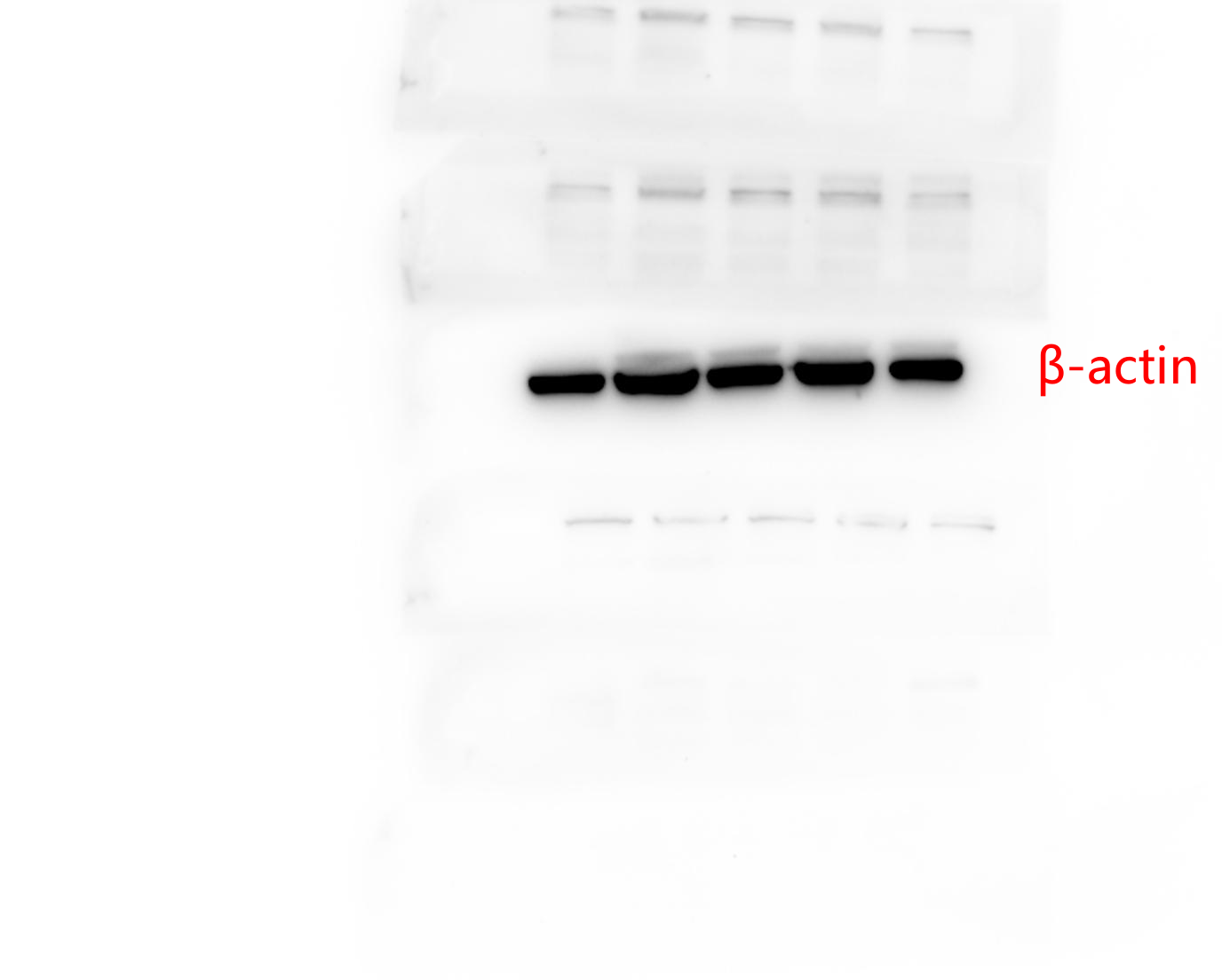

Supplement: Supplementary file 1 [file DataSheet1.ZIP › Supplementary Materials/supplementary materials ( original western blot figures)/Figure 6 (original western blot figures)/β-actin (hippocampus of LPS model).tif]

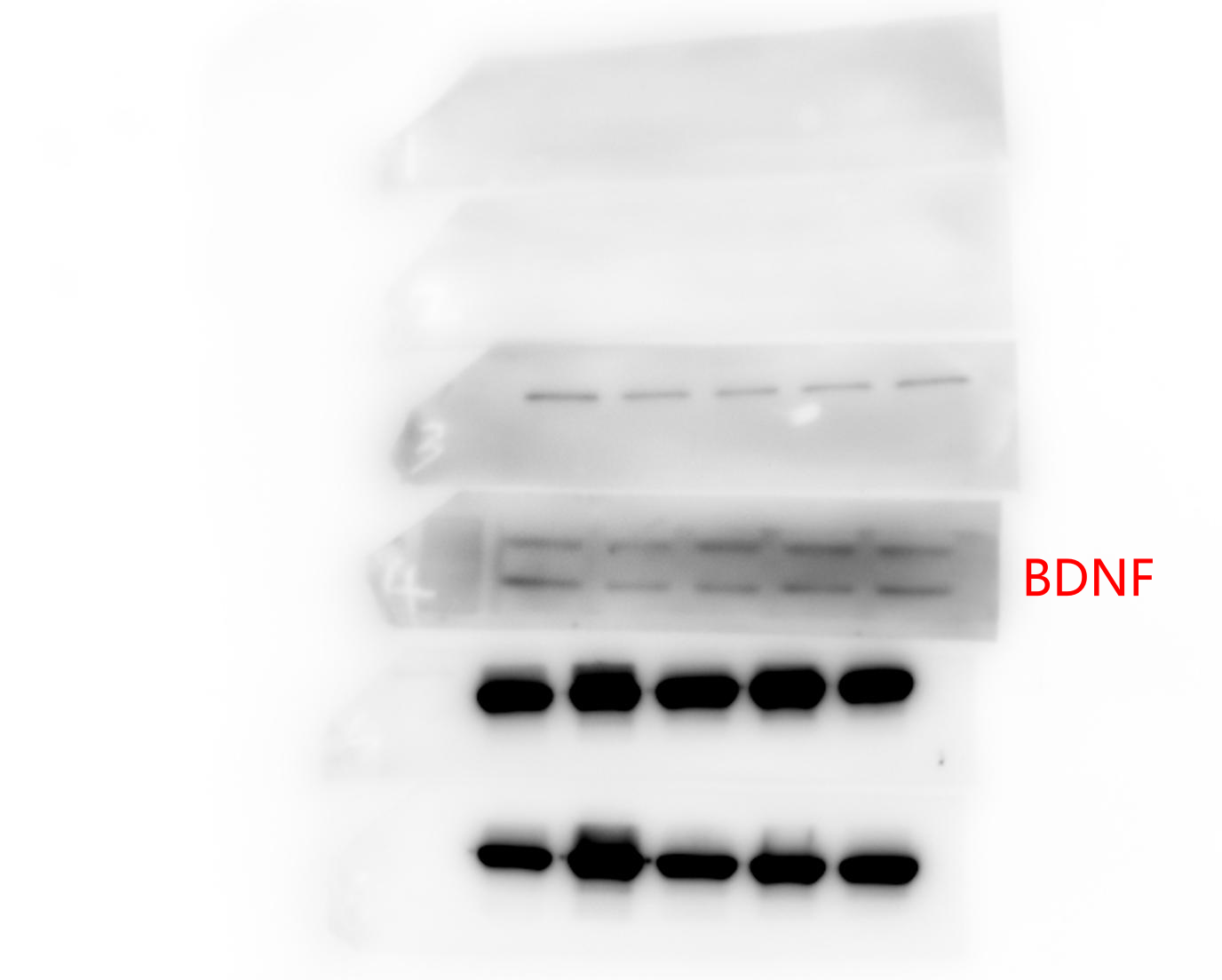

Supplement: Supplementary file 1 [file DataSheet1.ZIP › Supplementary Materials/supplementary materials ( original western blot figures)/Figure 7 (original western blot figures)/BDNF (cell supernatants).tif]

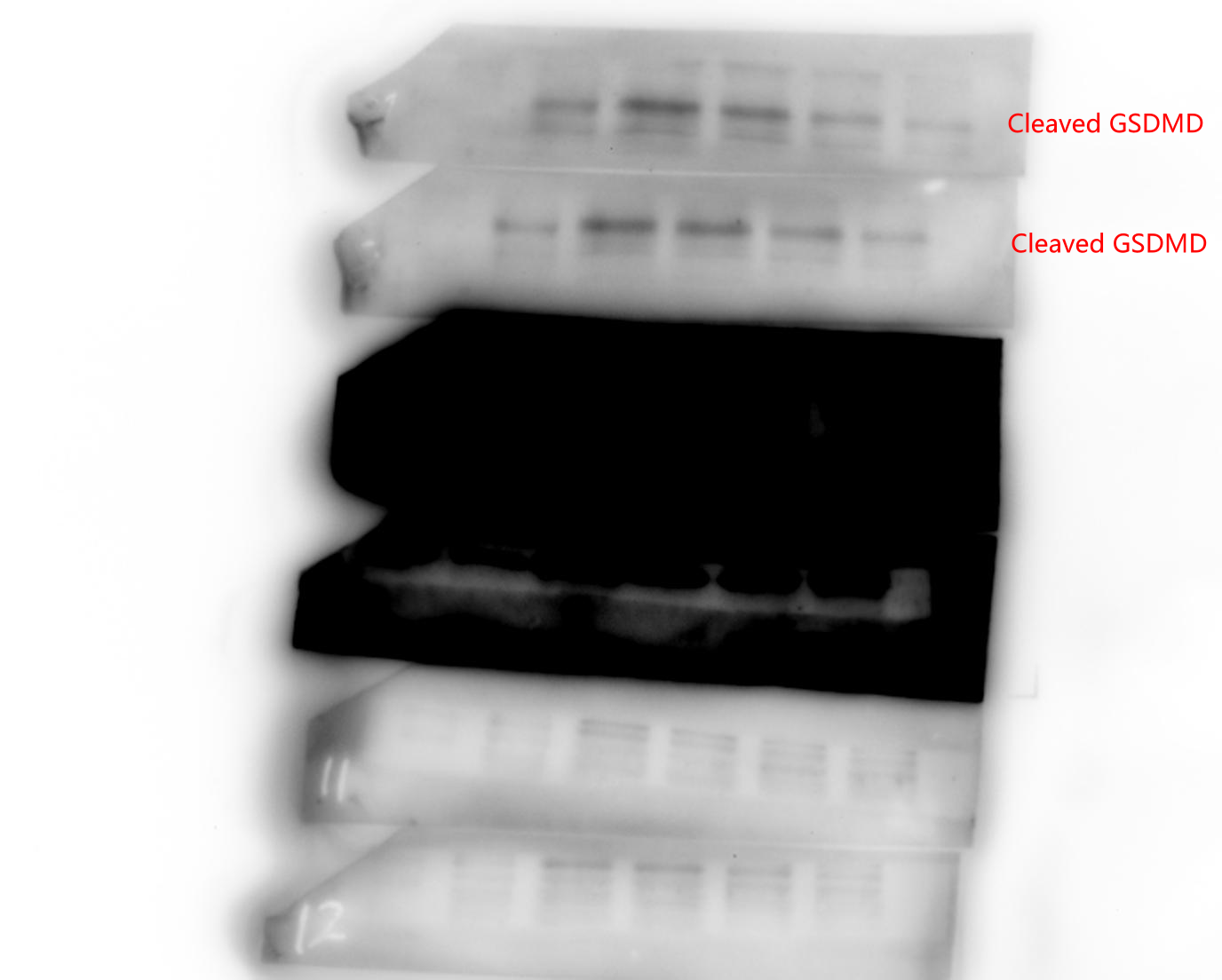

Supplement: Supplementary file 1 [file DataSheet1.ZIP › Supplementary Materials/supplementary materials ( original western blot figures)/Figure 7 (original western blot figures)/Cleaved GSDMD (cell supernatants).tif]

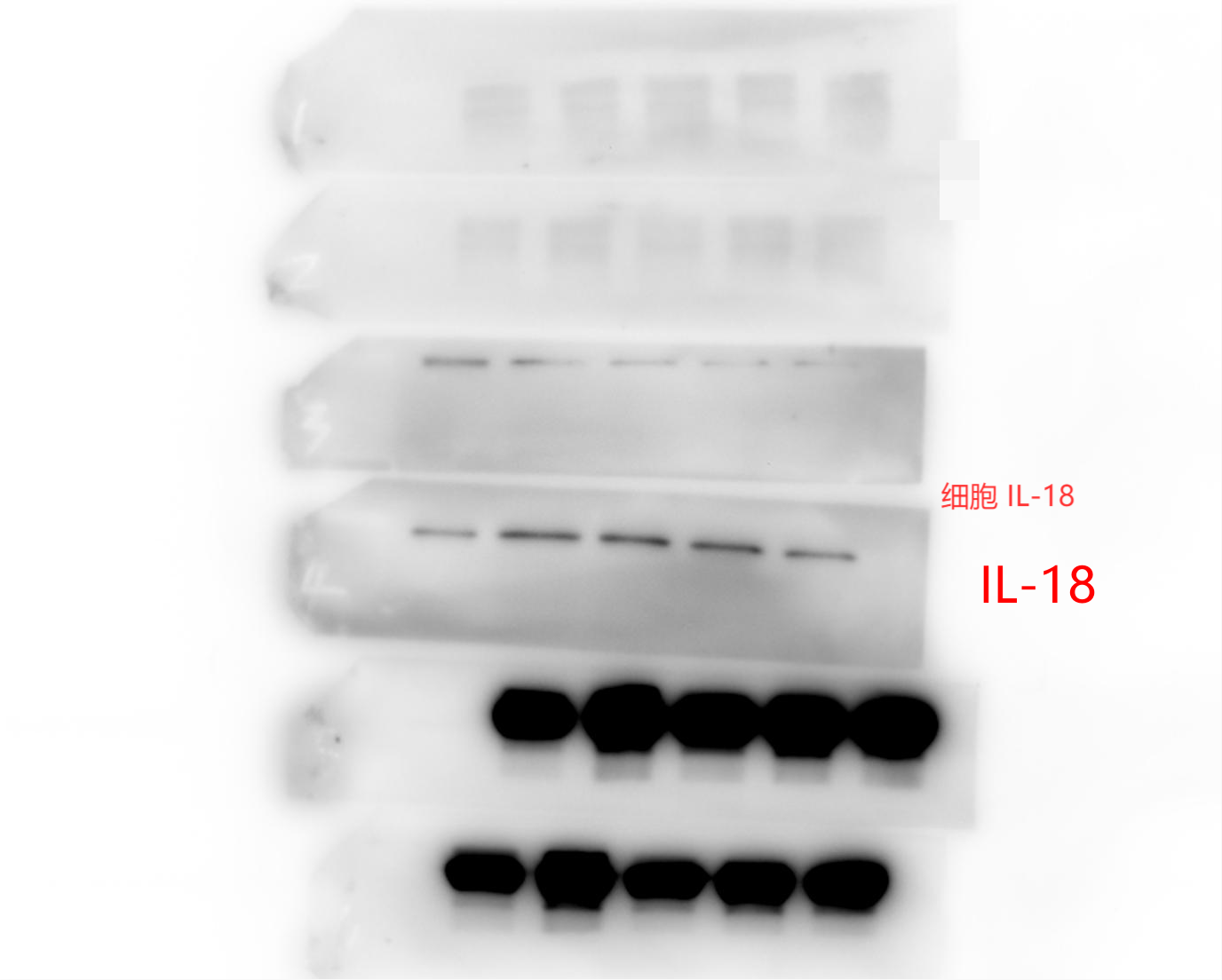

Supplement: Supplementary file 1 [file DataSheet1.ZIP › Supplementary Materials/supplementary materials ( original western blot figures)/Figure 7 (original western blot figures)/IL-18 (cell supernatants).tif]

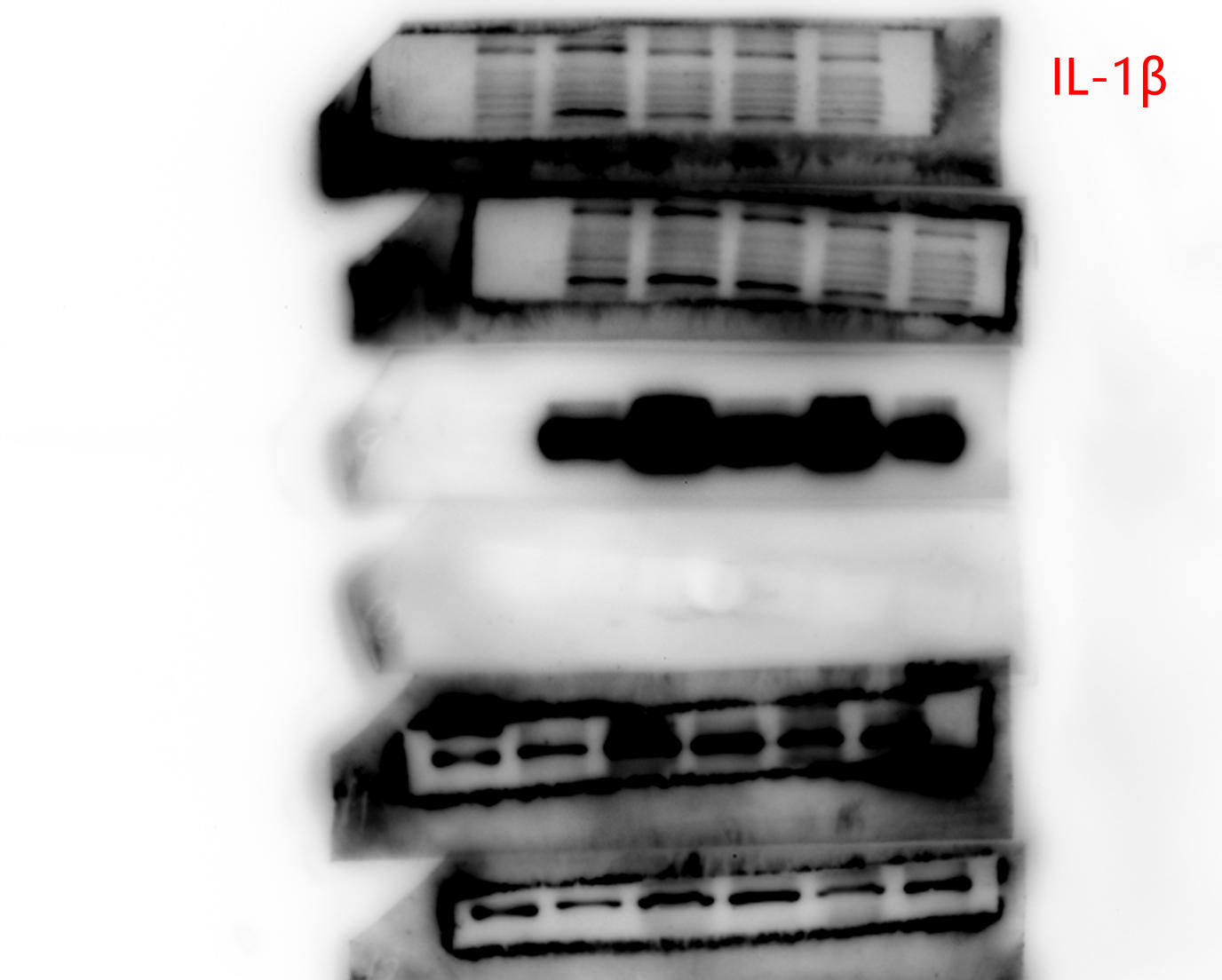

Supplement: Supplementary file 1 [file DataSheet1.ZIP › Supplementary Materials/supplementary materials ( original western blot figures)/Figure 7 (original western blot figures)/IL-1β (cell supernatants).tif]

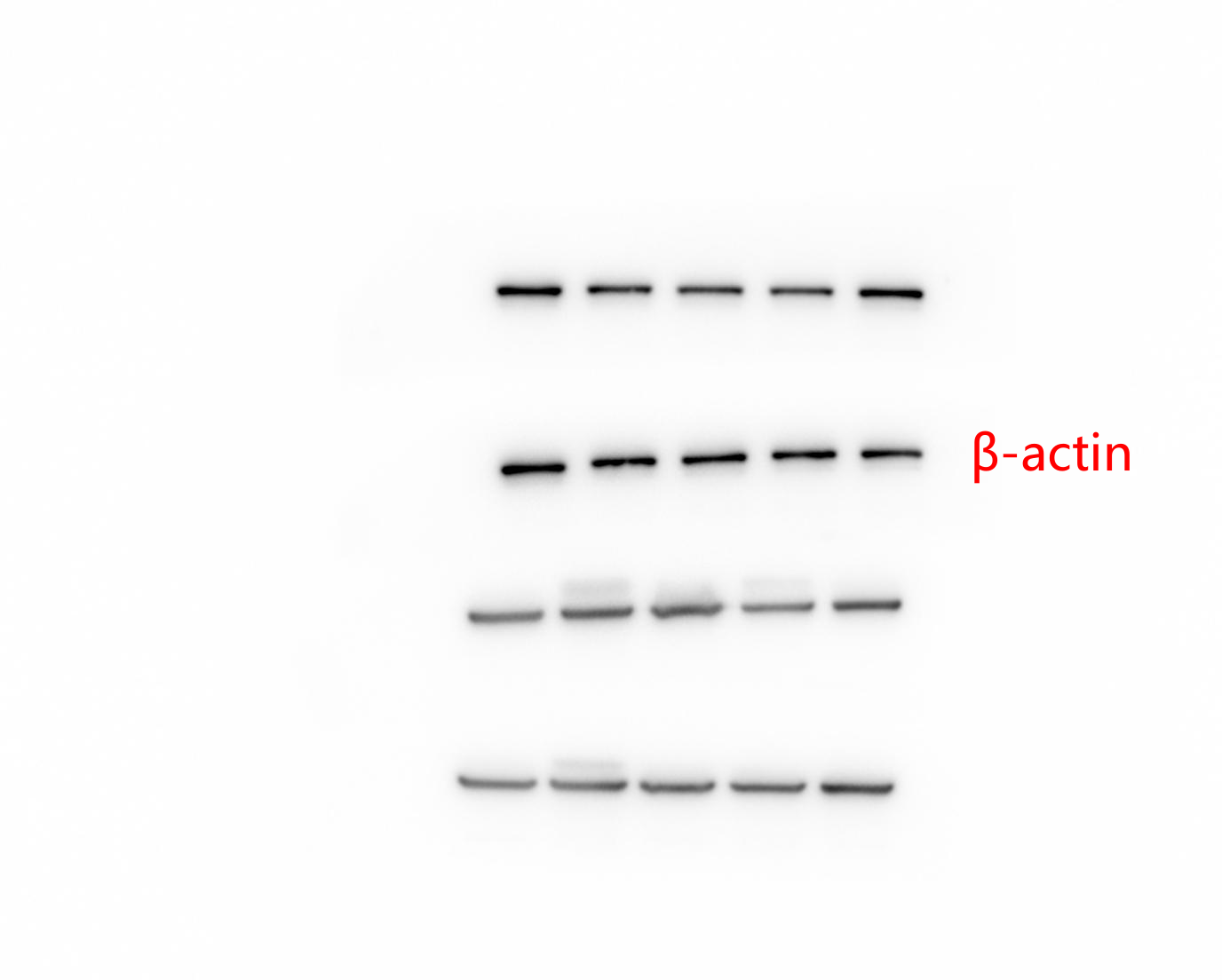

Supplement: Supplementary file 1 [file DataSheet1.ZIP › Supplementary Materials/supplementary materials ( original western blot figures)/Figure 7 (original western blot figures)/β-actin (cell supernatants figure 7D).tif]

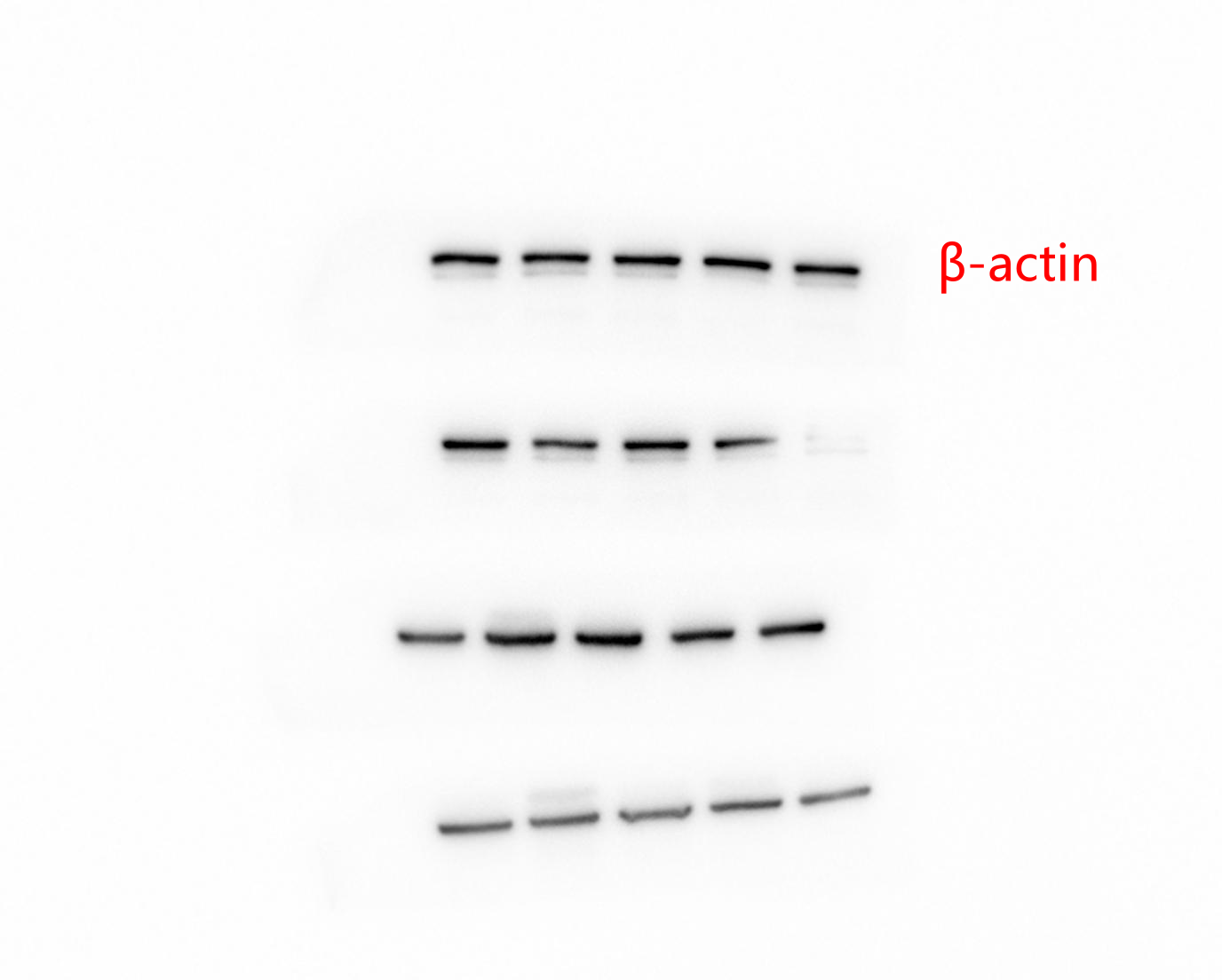

Supplement: Supplementary file 1 [file DataSheet1.ZIP › Supplementary Materials/supplementary materials ( original western blot figures)/Figure 7 (original western blot figures)/β-actin (cell supernatants figure 7B).tif]

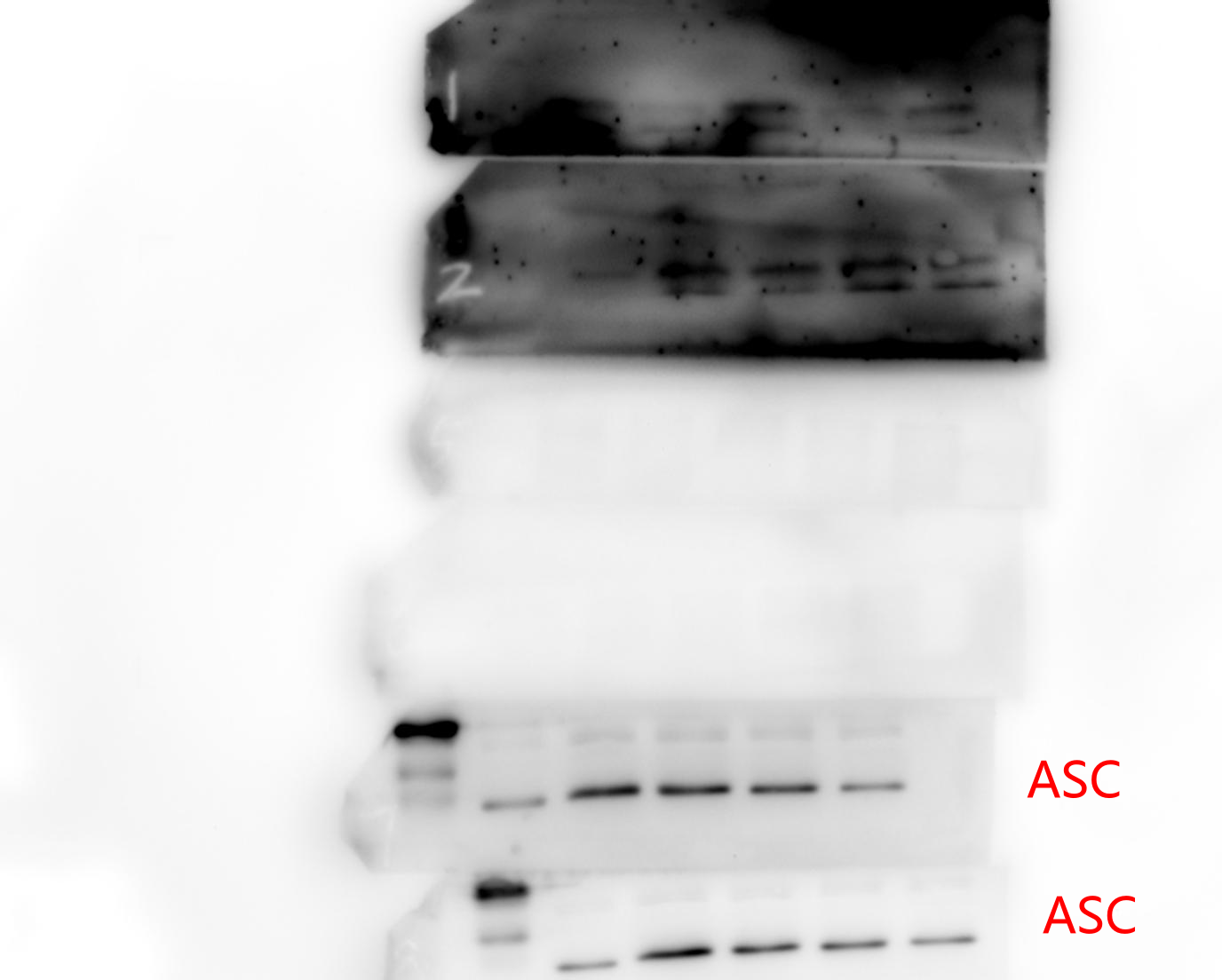

Supplement: Supplementary file 1 [file DataSheet1.ZIP › Supplementary Materials/supplementary materials ( original western blot figures)/Figure 8 (original western blot figures)/Figure 8A (original western blot figures)/ASC (cell supernatants).tif]

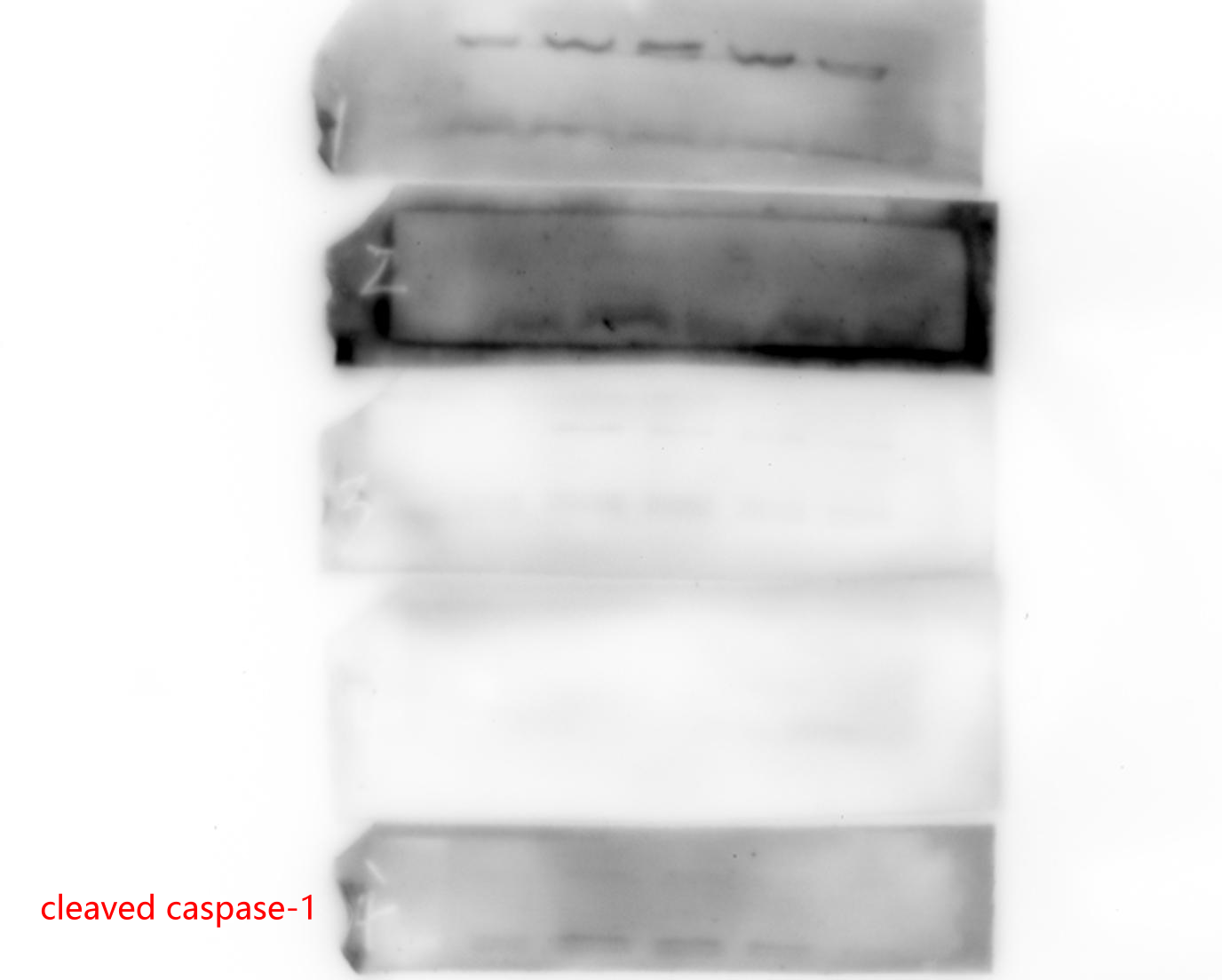

Supplement: Supplementary file 1 [file DataSheet1.ZIP › Supplementary Materials/supplementary materials ( original western blot figures)/Figure 8 (original western blot figures)/Figure 8A (original western blot figures)/Cleaved Caspase-1 (cell supernatants).tif]

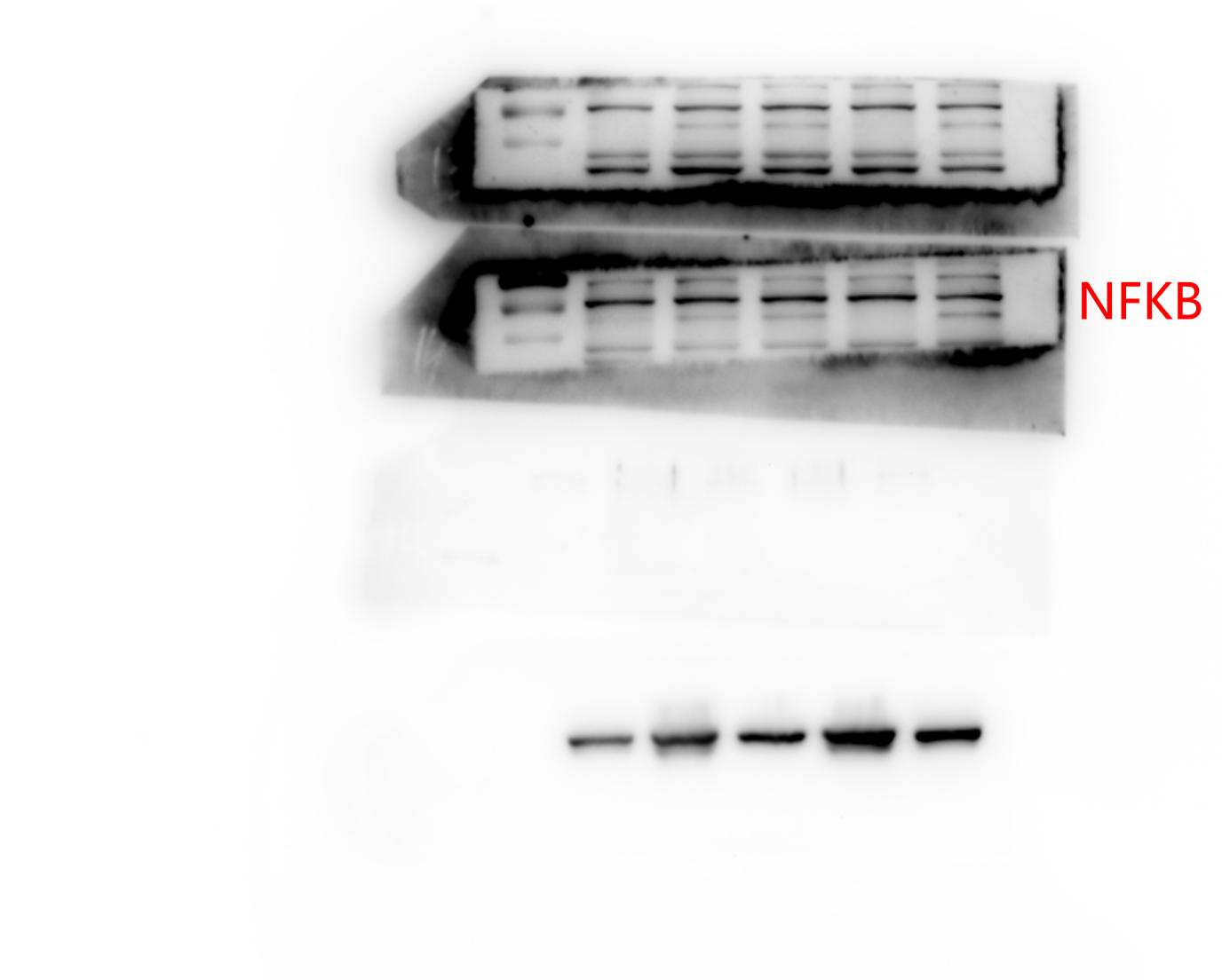

Supplement: Supplementary file 1 [file DataSheet1.ZIP › Supplementary Materials/supplementary materials ( original western blot figures)/Figure 8 (original western blot figures)/Figure 8A (original western blot figures)/NF-κB (cell supernatants).tif]

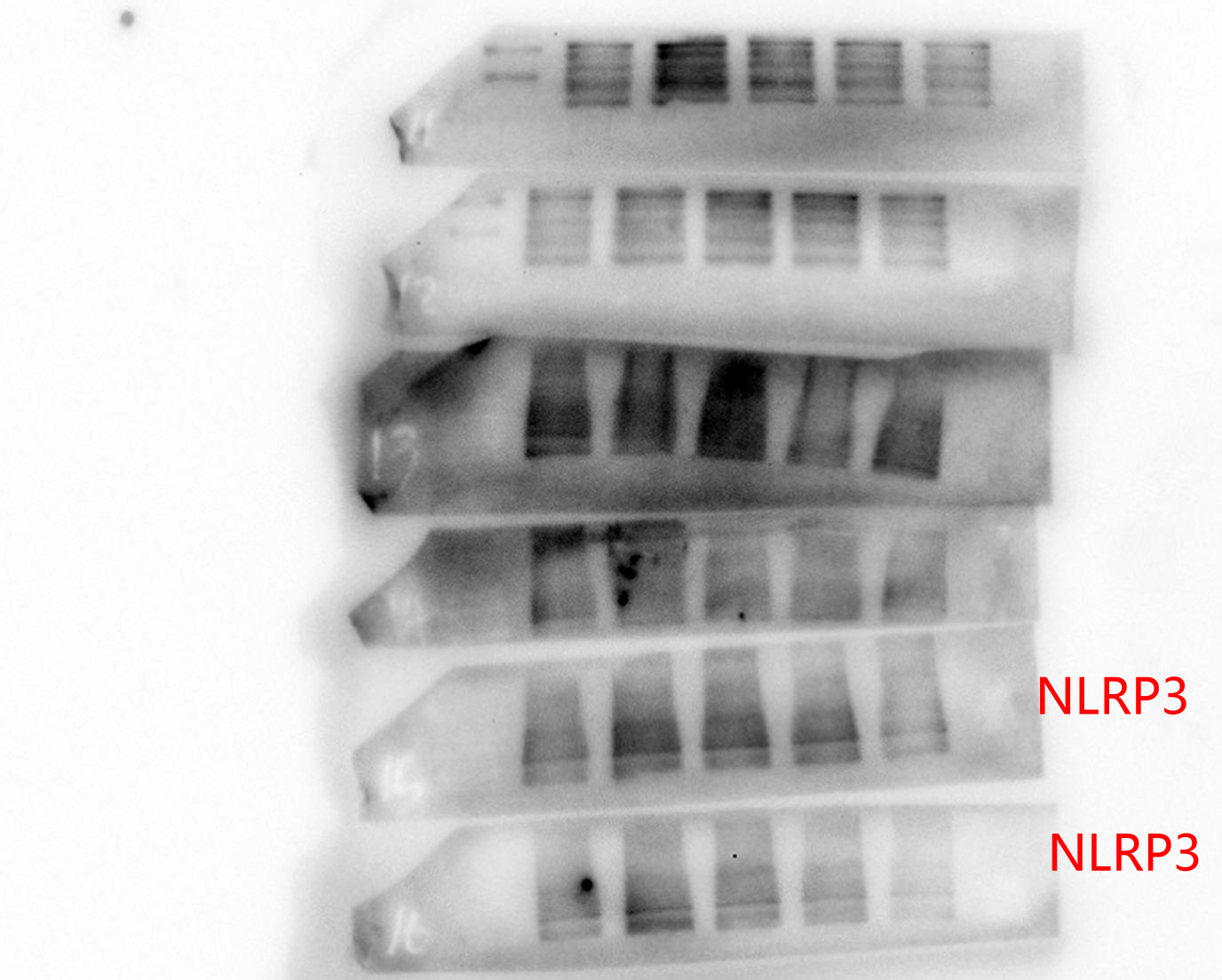

Supplement: Supplementary file 1 [file DataSheet1.ZIP › Supplementary Materials/supplementary materials ( original western blot figures)/Figure 8 (original western blot figures)/Figure 8A (original western blot figures)/NLRP3 (cell supernatants).tif]

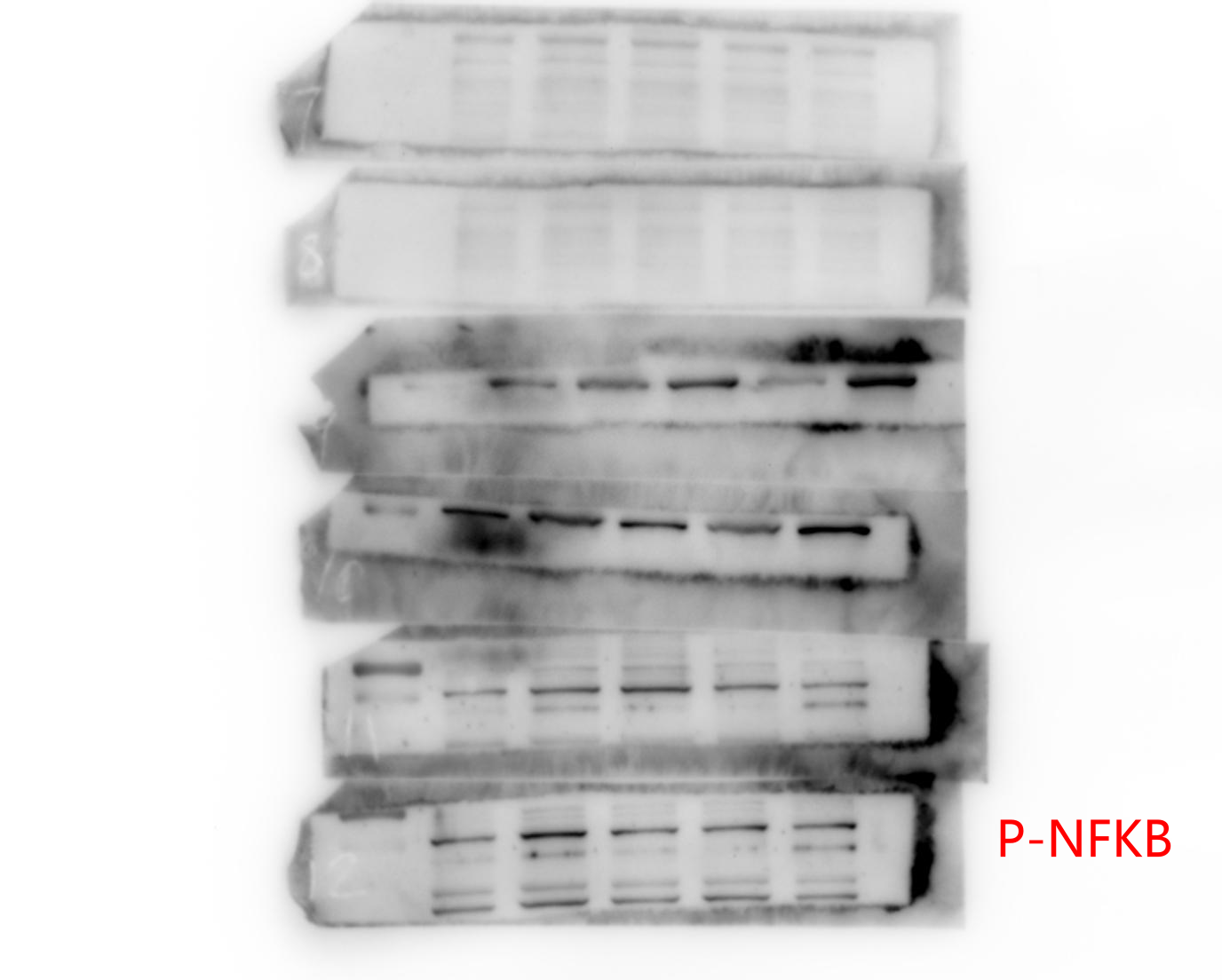

Supplement: Supplementary file 1 [file DataSheet1.ZIP › Supplementary Materials/supplementary materials ( original western blot figures)/Figure 8 (original western blot figures)/Figure 8A (original western blot figures)/P-NF-κB (cell supernatants).tif]

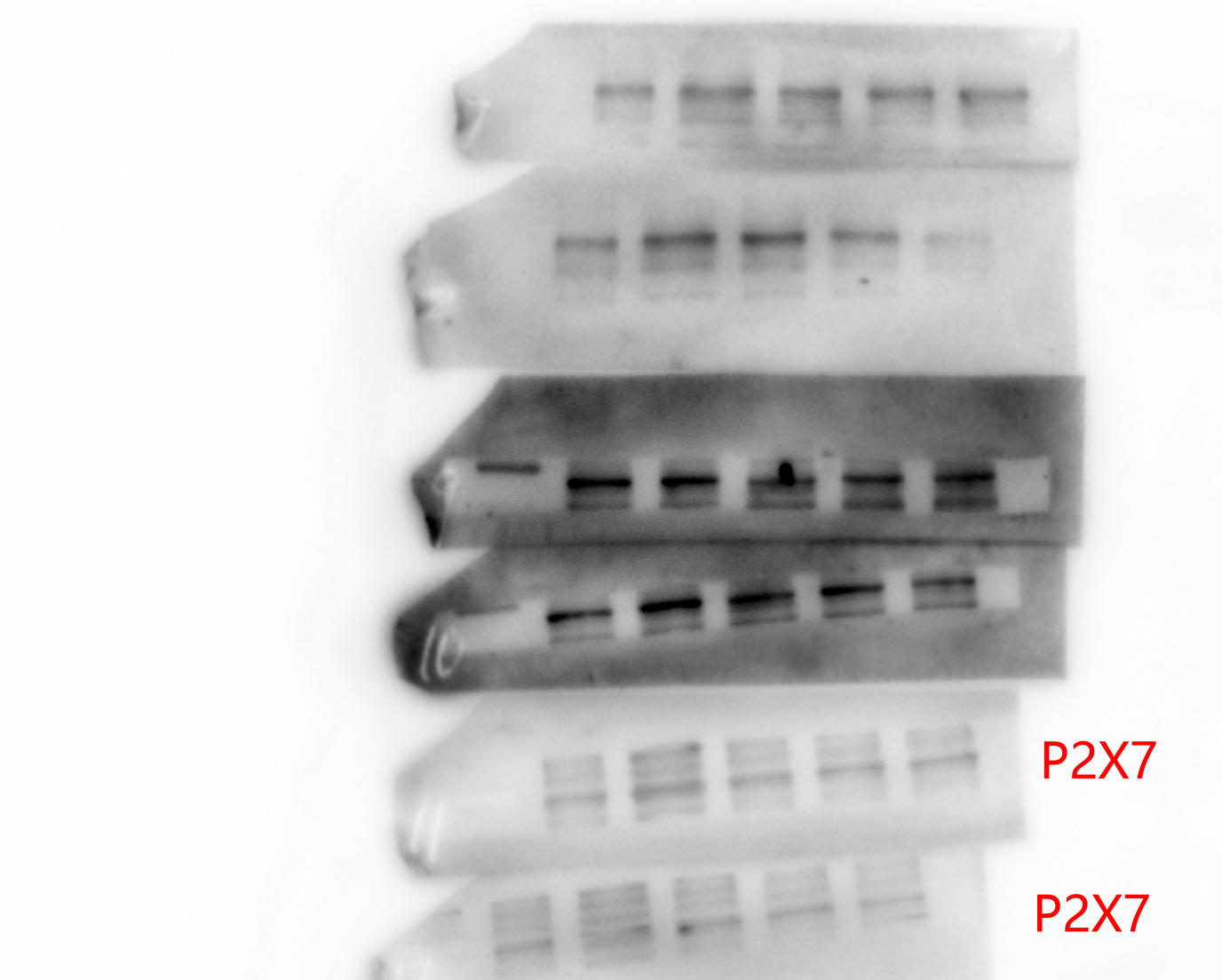

Supplement: Supplementary file 1 [file DataSheet1.ZIP › Supplementary Materials/supplementary materials ( original western blot figures)/Figure 8 (original western blot figures)/Figure 8A (original western blot figures)/P2X7 (cell supernatants).tif]

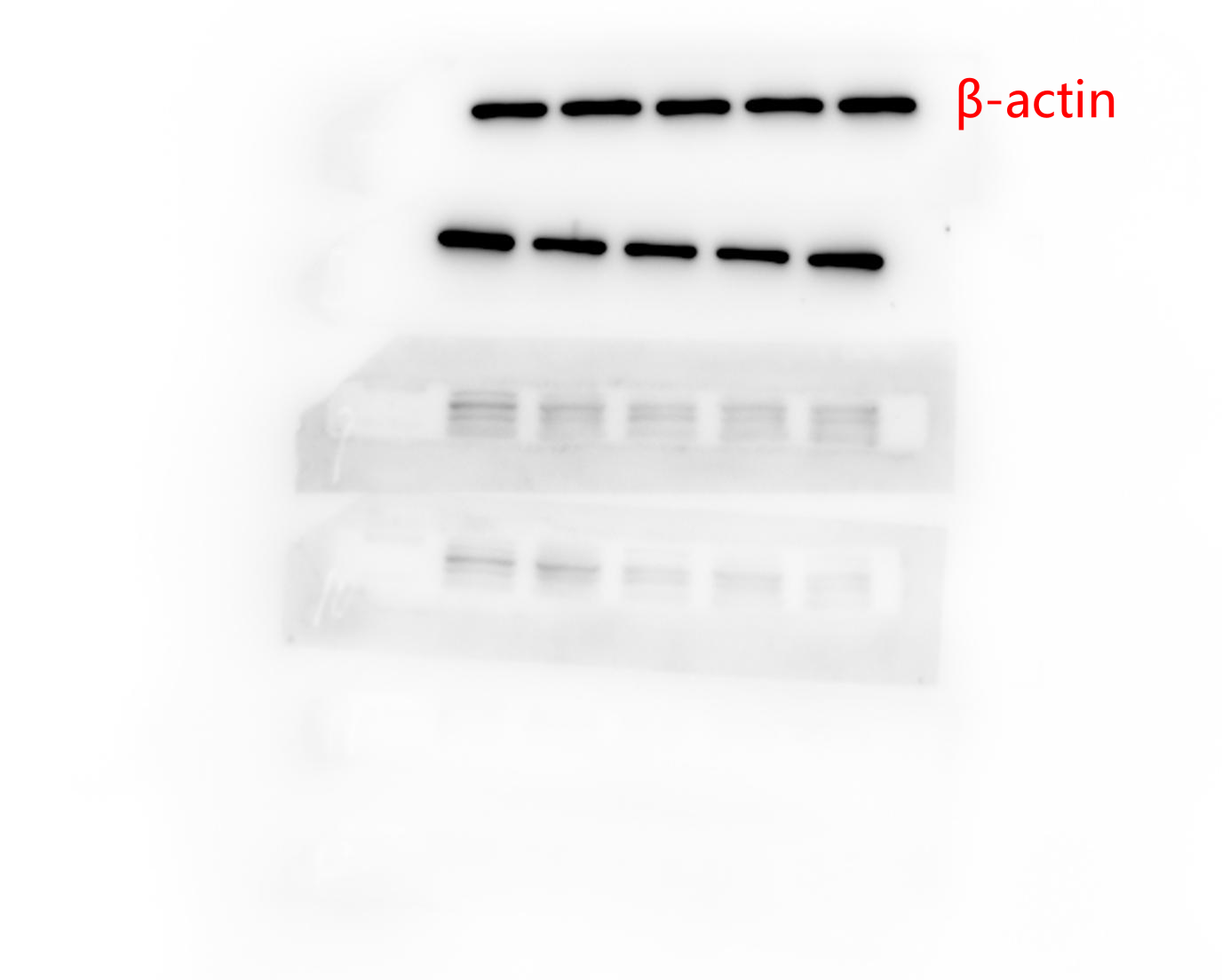

Supplement: Supplementary file 1 [file DataSheet1.ZIP › Supplementary Materials/supplementary materials ( original western blot figures)/Figure 8 (original western blot figures)/Figure 8A (original western blot figures)/β-actin (cell supernatants).tif]

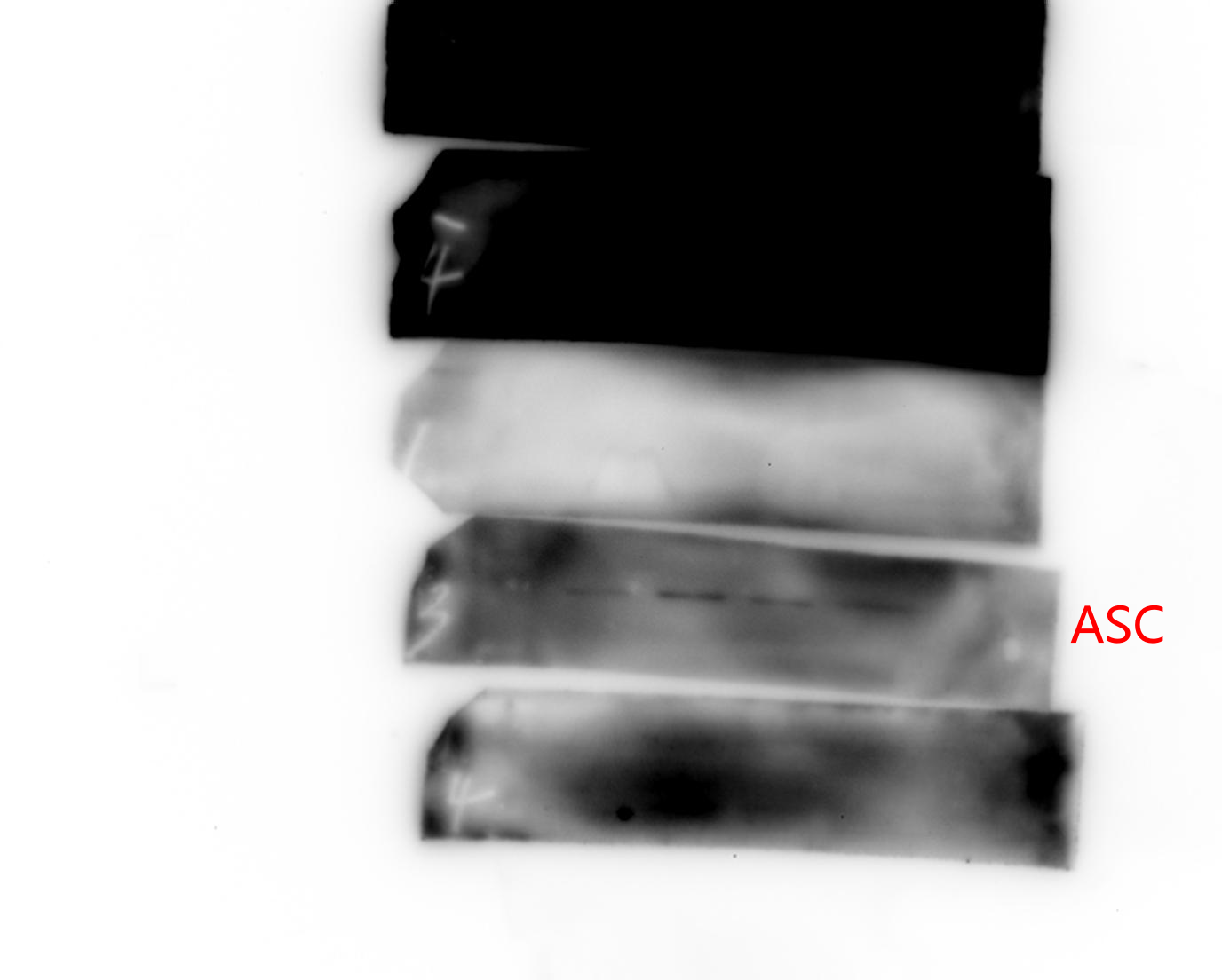

Supplement: Supplementary file 1 [file DataSheet1.ZIP › Supplementary Materials/supplementary materials ( original western blot figures)/Figure 8 (original western blot figures)/Figure 8E (original western blot figures)/ASC (cell supernatants).tif]

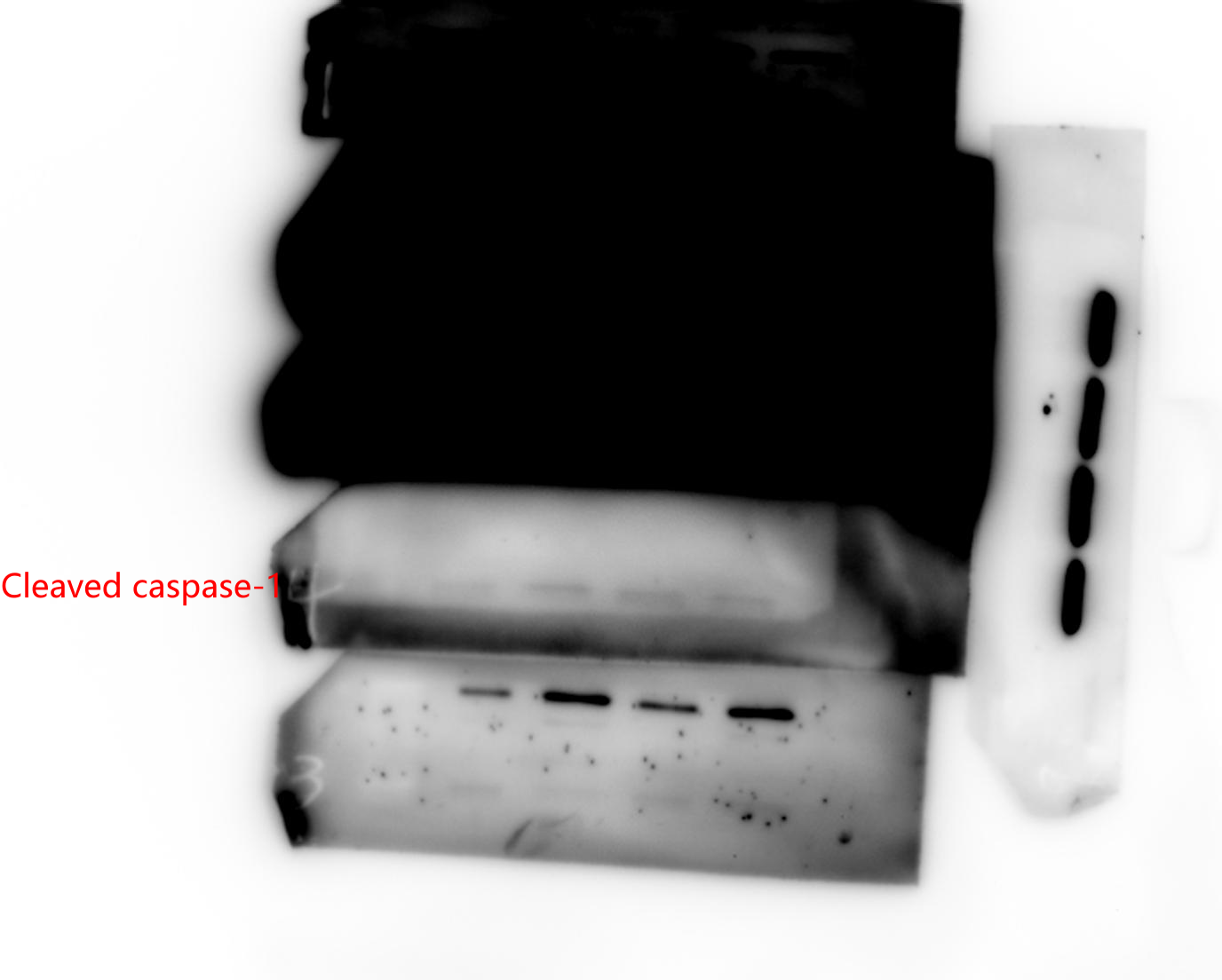

Supplement: Supplementary file 1 [file DataSheet1.ZIP › Supplementary Materials/supplementary materials ( original western blot figures)/Figure 8 (original western blot figures)/Figure 8E (original western blot figures)/Cleaved caspase-1 (cell supernatants).tif]

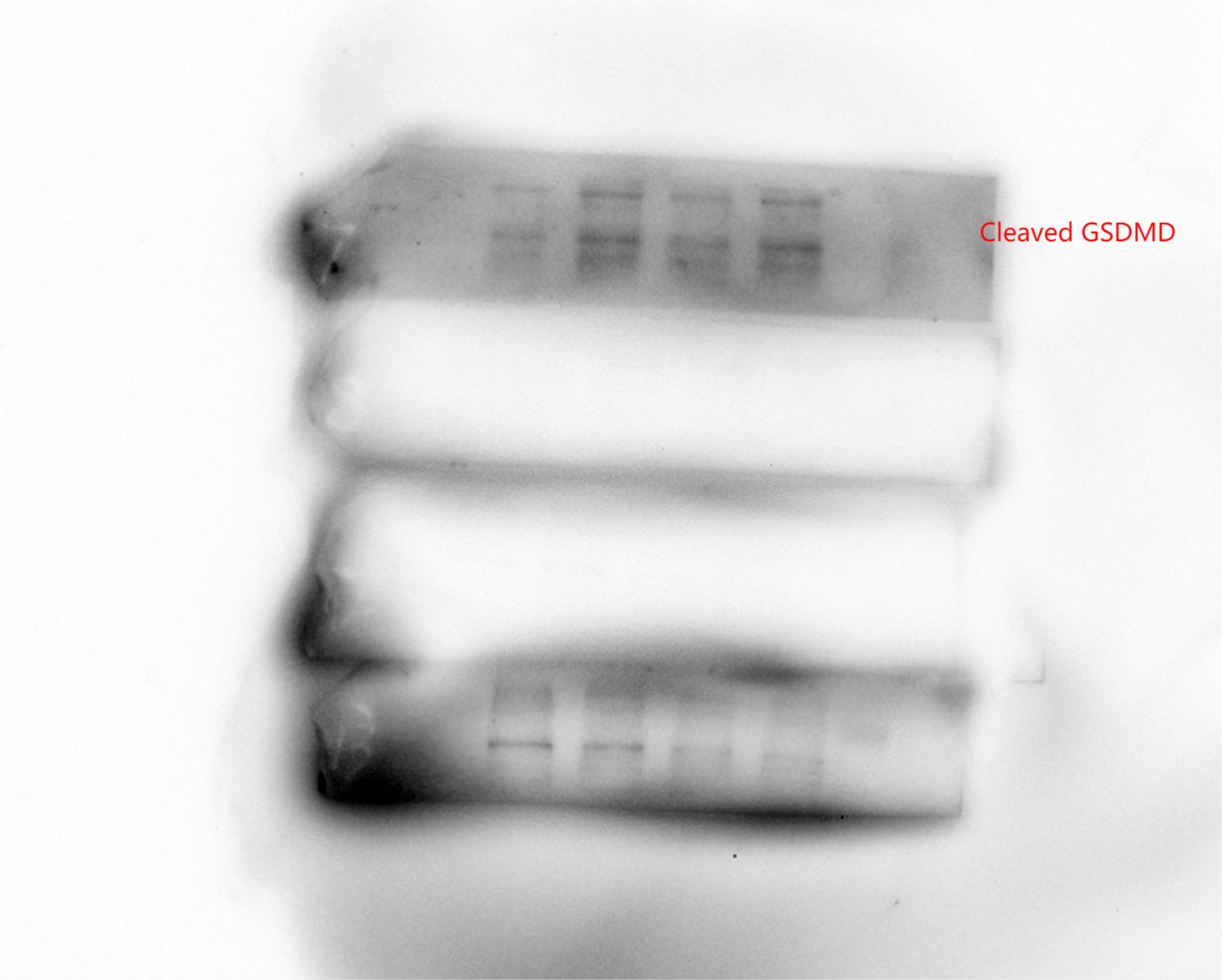

Supplement: Supplementary file 1 [file DataSheet1.ZIP › Supplementary Materials/supplementary materials ( original western blot figures)/Figure 8 (original western blot figures)/Figure 8E (original western blot figures)/Cleaved GSDMD (cell supernatants).png]

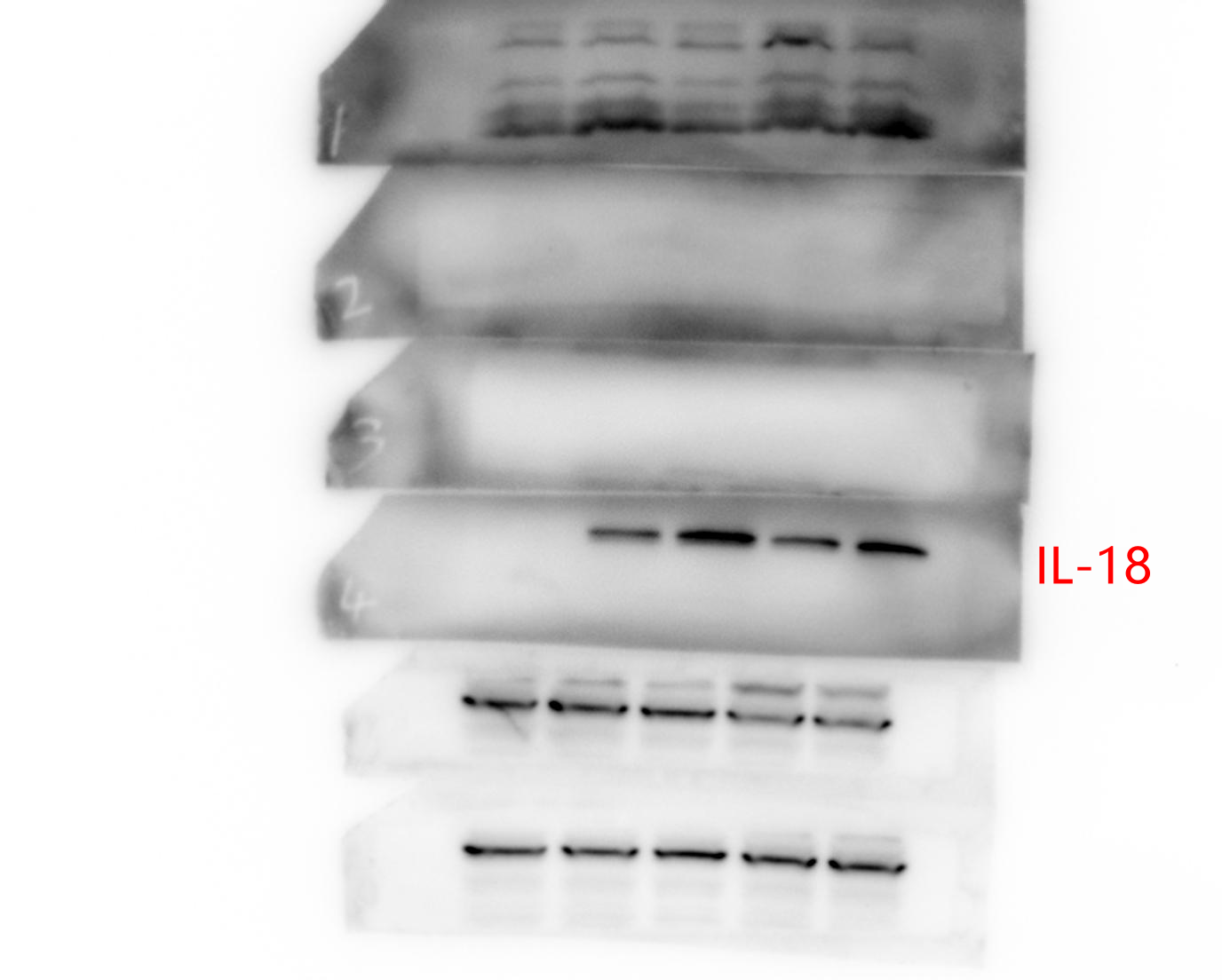

Supplement: Supplementary file 1 [file DataSheet1.ZIP › Supplementary Materials/supplementary materials ( original western blot figures)/Figure 8 (original western blot figures)/Figure 8E (original western blot figures)/IL-18 (cell supernatants).tif]

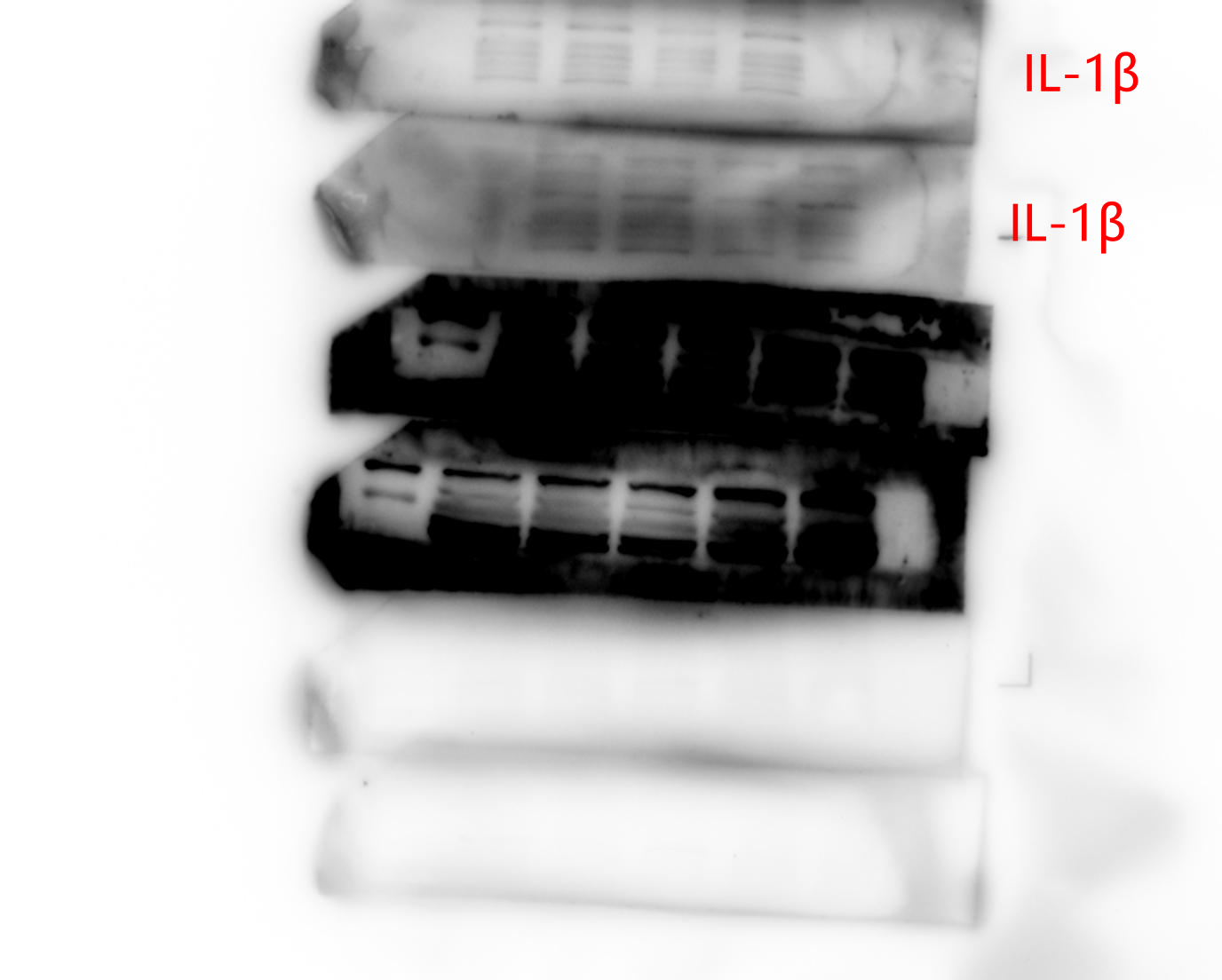

Supplement: Supplementary file 1 [file DataSheet1.ZIP › Supplementary Materials/supplementary materials ( original western blot figures)/Figure 8 (original western blot figures)/Figure 8E (original western blot figures)/IL-1β (cell supernatants).tif]

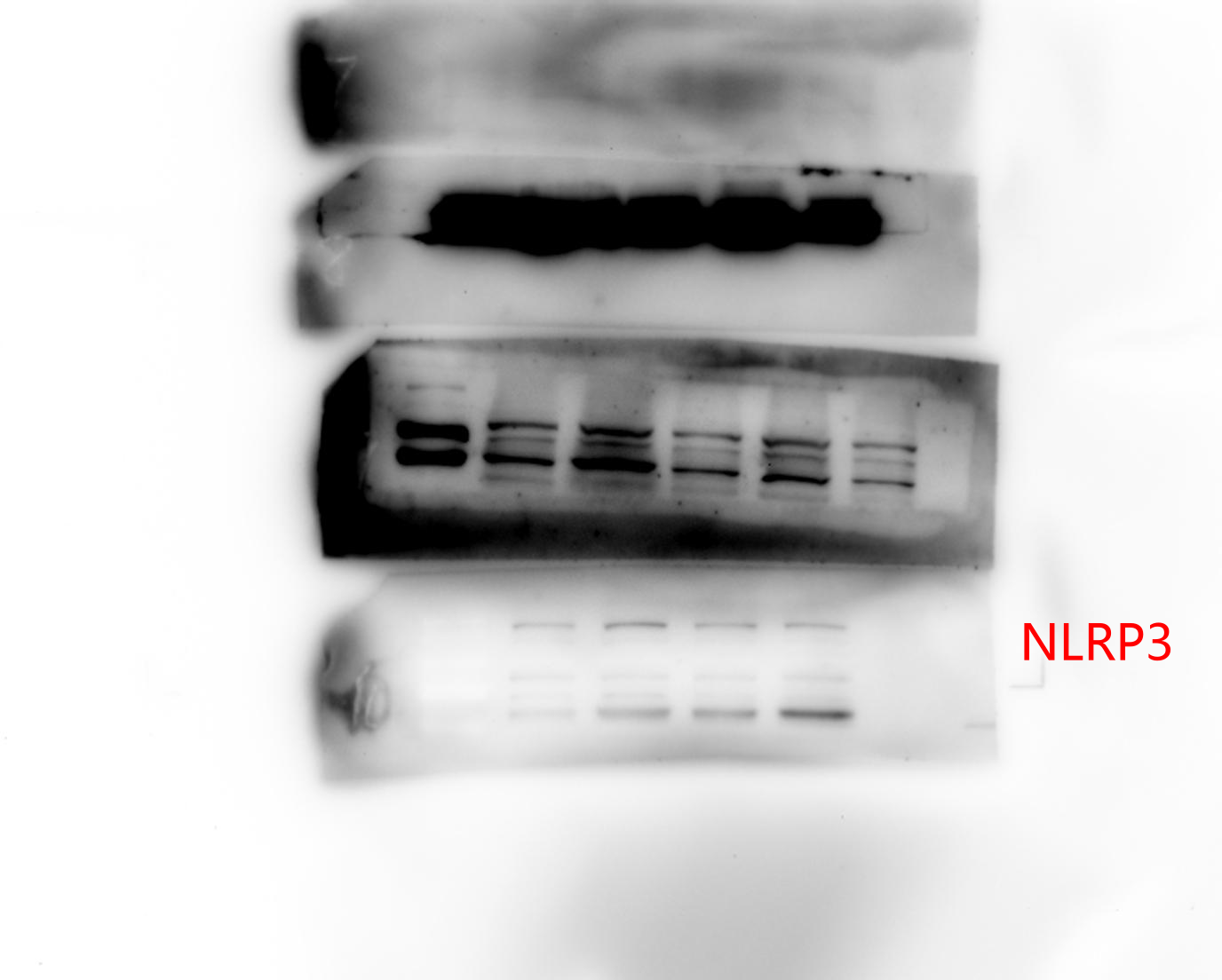

Supplement: Supplementary file 1 [file DataSheet1.ZIP › Supplementary Materials/supplementary materials ( original western blot figures)/Figure 8 (original western blot figures)/Figure 8E (original western blot figures)/NLRP3 (cell supernatants).tif]

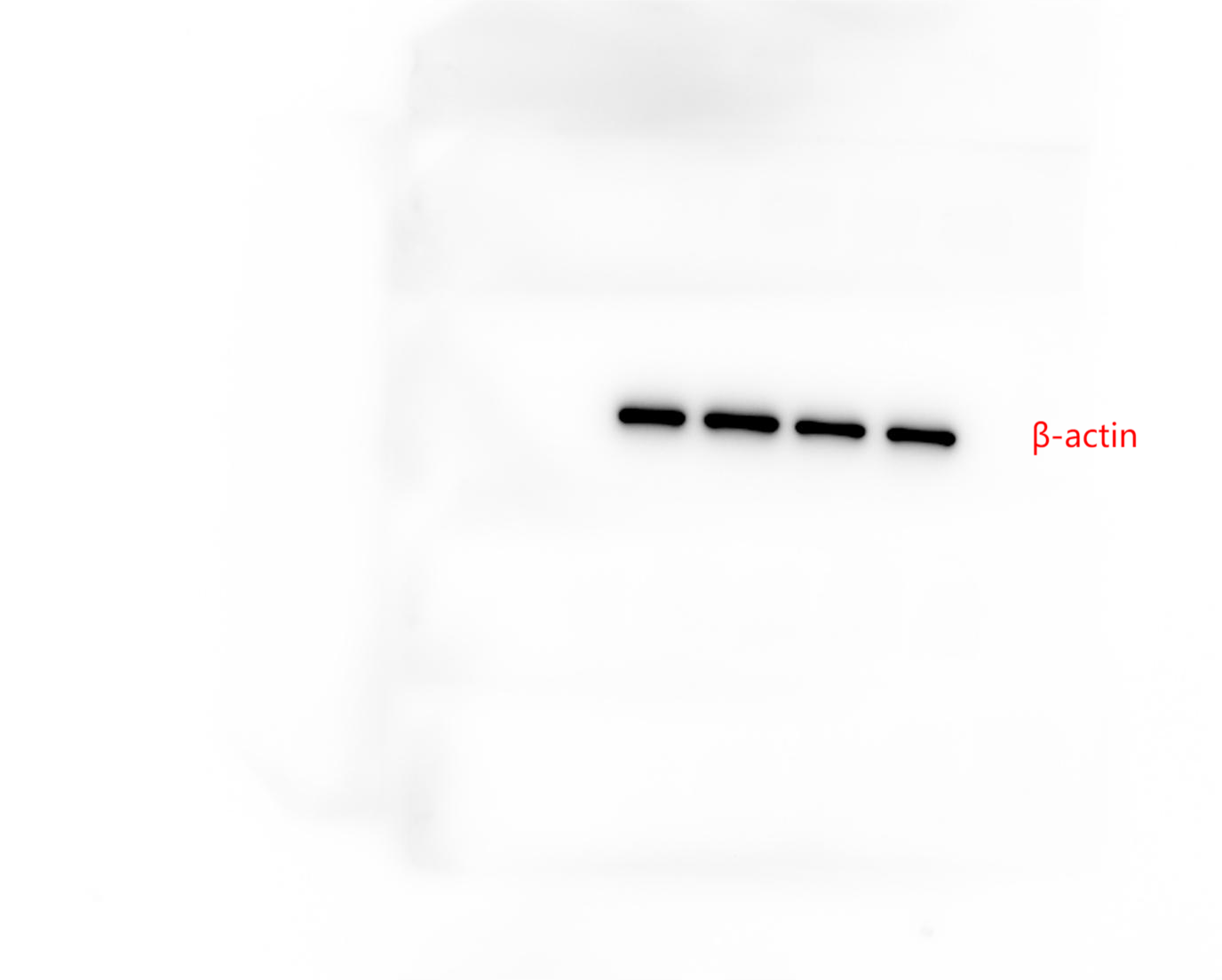

Supplement: Supplementary file 1 [file DataSheet1.ZIP › Supplementary Materials/supplementary materials ( original western blot figures)/Figure 8 (original western blot figures)/Figure 8E (original western blot figures)/β-actin (cell supernatants).png]
