# Supplementary material for: Salidroside Ameliorates Depression by Suppressing NLRP3-Mediated Pyroptosis via P2X7/NF-κB/NLRP3 Signaling Pathway
Source: Front Pharmacol. 2022 Apr 12;13:812362. doi: 10.3389/fphar.2022.812362 (PMC9039222; doi:10.3389/fphar.2022.812362)
Supplement: Supplementary file 2 [file DataSheet2.ZIP › Supplementary materials/supplementary materials 2 ( original western blot figures).pdf]

Supplementary materials 2 (original western blot figures):

Legends of the figure

FIGURE 1 | Effects of Sal in CORT-induced depression in mice.

The original western blot bands of BDNF, β-actin protein in CORT-induced depressive mice.

The lane order from left to right on blot is: (1) the control group, (2) the CORT group, (3) the CORT + FLU group, (4) the CORT + Sal (20 mg/kg) group, (5) the CORT + Sal (40 mg/kg) group.

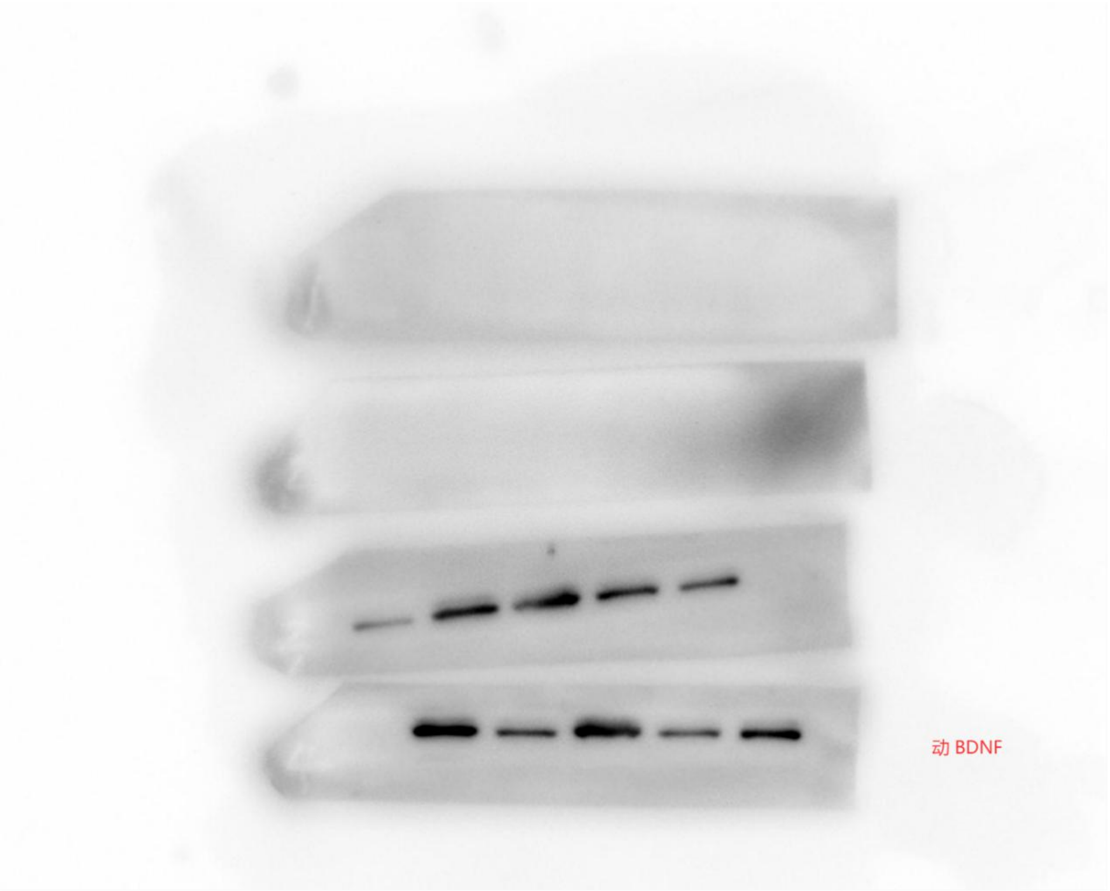

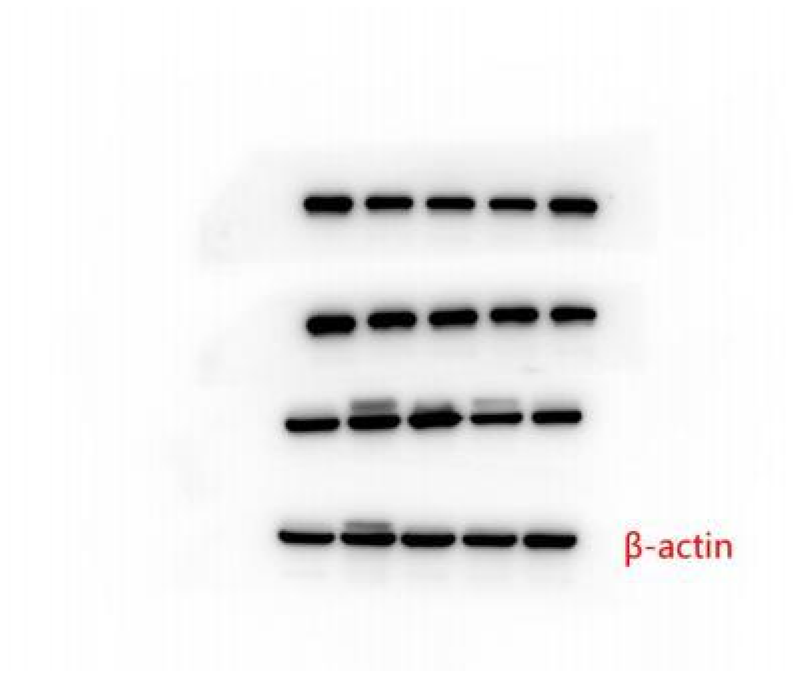

FIGURE 2 | Sal mitigates pyroptosis in CORT-induced depression in mice.

The original western blot bands of IL- 1β, IL- 18 protein in CORT-induced depressive mice.

The lane order from left to right on blot is: (1) the control group, (2) the CORT group, (3) the CORT + FLU group, (4) the CORT + Sal (20 mg/kg) group, (5) the CORT + Sal (40 mg/kg) group.

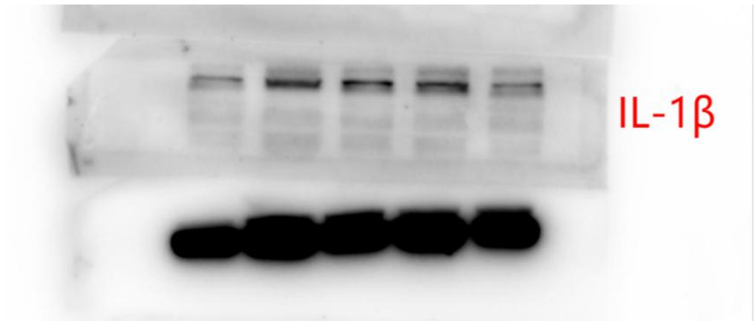

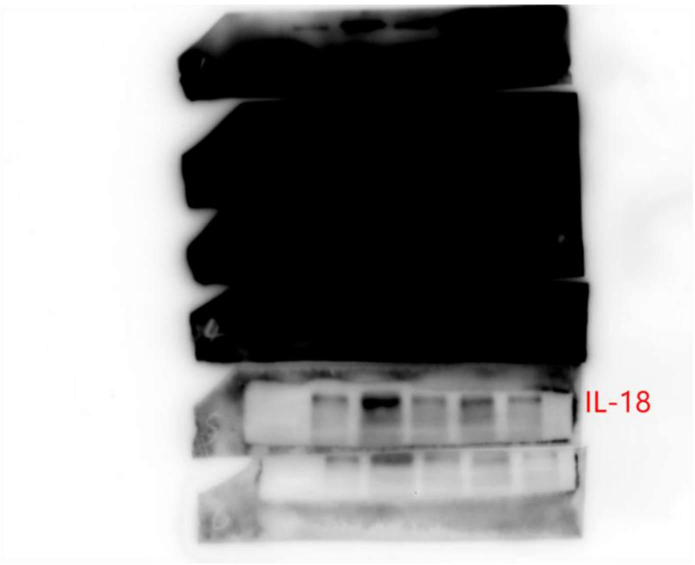

FIGURE 6 | Sal inhibits the P2X7/NF-κB/NLRP3 signaling pathway in LPS-induced mice. The original western blot band of ASC protein in LPS-induced depressive mice.

The lane order from left to right on blot is: (1) the control group, (2) the LPS group, (3) the LPS + FLU group, (4) the LPS + Sal (20 mg/kg) group, (5) the LPS + Sal (40 mg/kg) group.

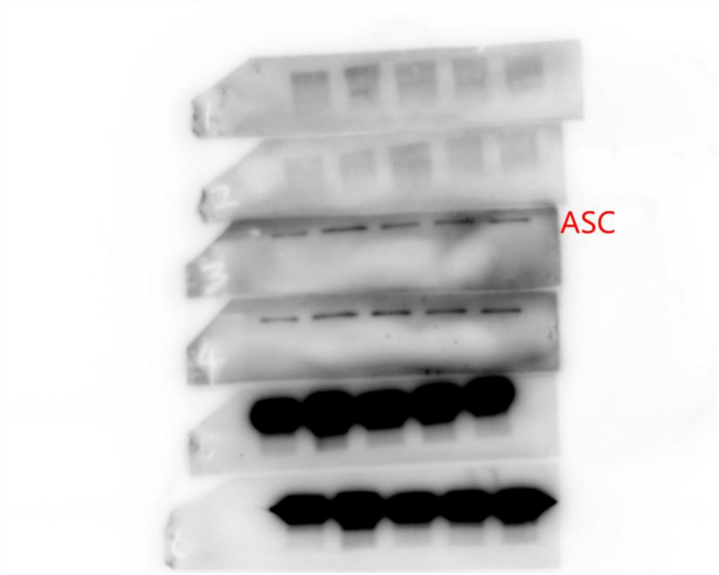

FIGURE 8 | Sal ameliorates pyroptosis by inhibiting P2X7/NF-κB/NLRP3 signaling pathway in CORT-induced PC12 cells.

(A) The original western blot band of Cleaved caspase-1. The lane order from left to

right on blot is: (1) the control group, (2) the CORT group, (3) the 2 μM Sal group, (4) the CORT + 10 μM Sal group, (5) the CORT + 50 μM Sal group.

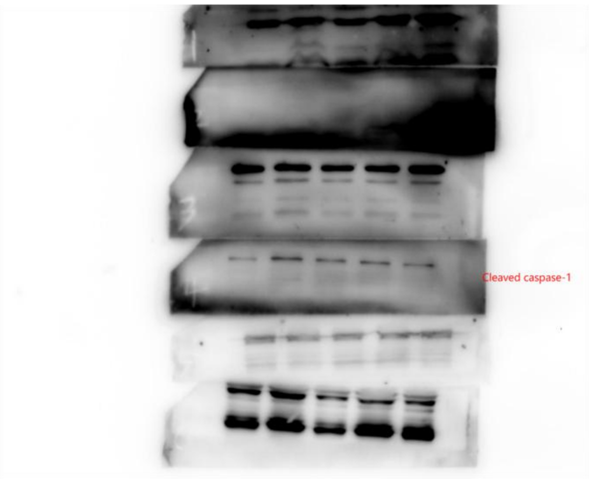

(E) The protein levels of NLRP3, ASC, Cleaved caspase-1, IL-1β, IL-18 and Cleaved GSDMD were detected using Western blot (n = 3).

The original western blot bands of NLRP3, ASC, Cleaved caspase-1, IL-1β, IL-18, Cleaved GSDMD and β-actin. The lane order from left to right on blot is: (1) the Control group, (2) the CORT group, (3) the Nig group, (4) the CORT + Sal group, (5) the Nig + Sal group, (6) the CORT + Sal + Nig group.

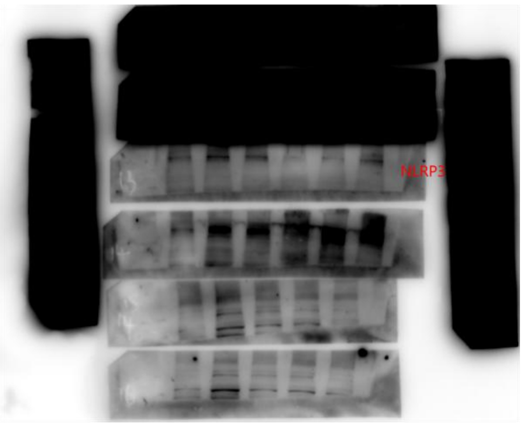

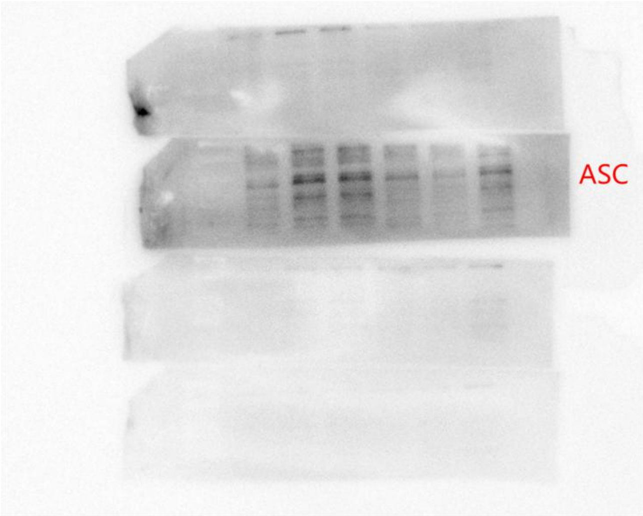
ASC

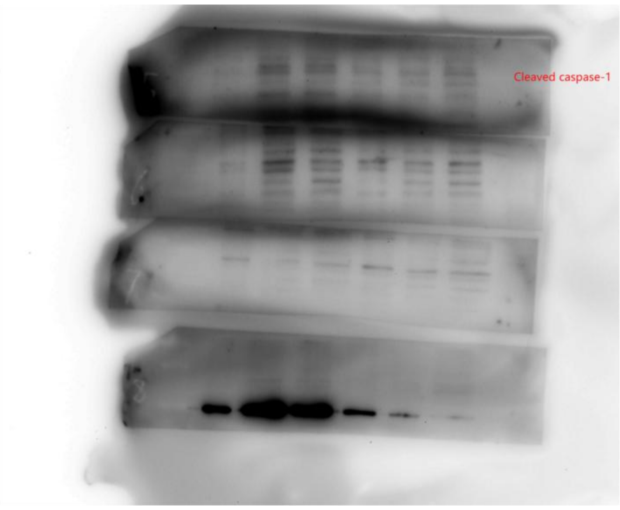
Cleaved caspase-1

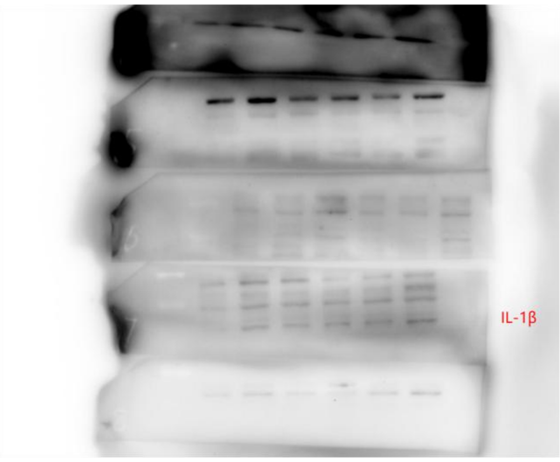
IL-1β

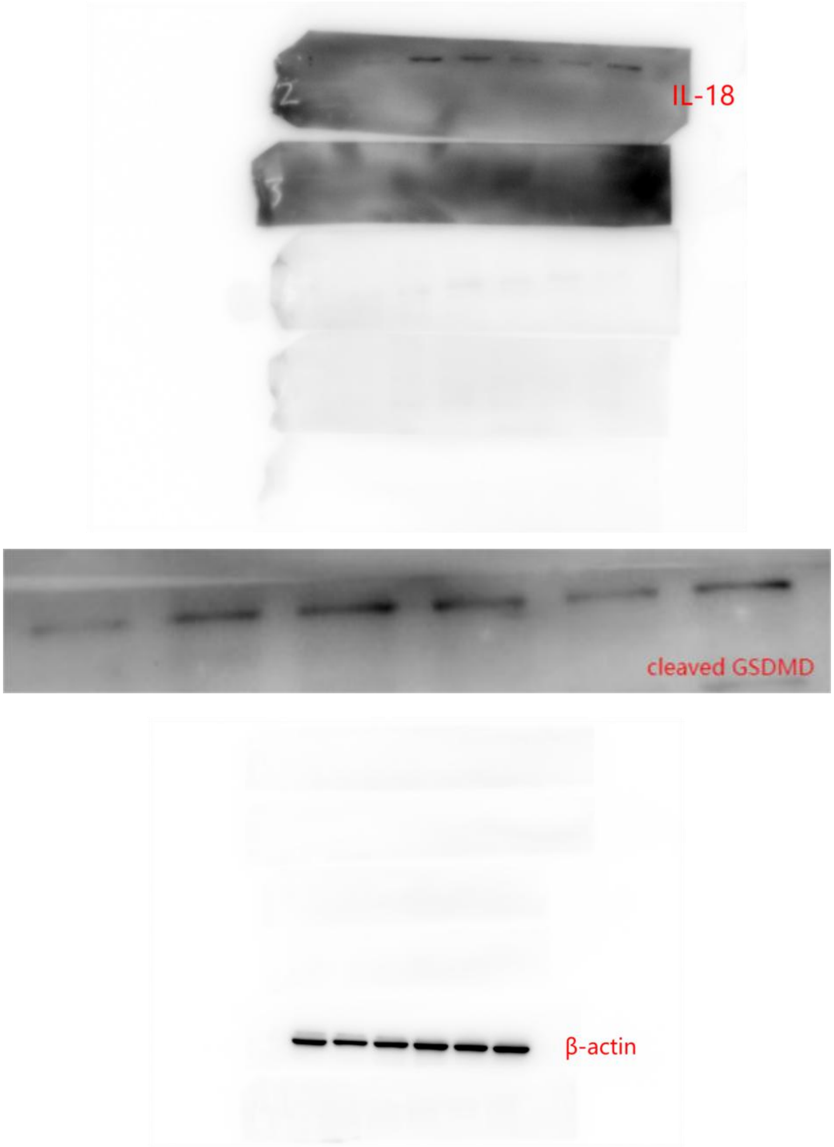

FIGURE 9 | Sal and knockdown of NLRP3 attenuates CORT-induced pyroptosis in PC12 cells.

(A) Western blot analysis was used to detect the protein levels of NLRP3 in knock down of NLRP3 in PC12 cells (n=3).

The original western blot bands of NLRP3 and β-actin protein in PC12 cells. The lane order from left to right on blot is: (1) the Control group, (2) the Si-NC group, (3) the Si-NLRP3 3 group, (4) the Si-NLRP3 2 group, (5) the Si-NLRP3 1 group.

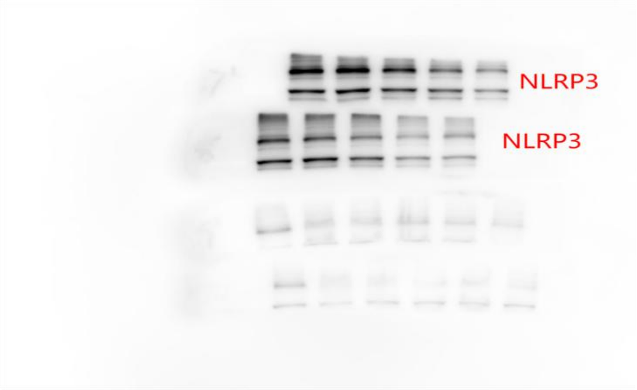

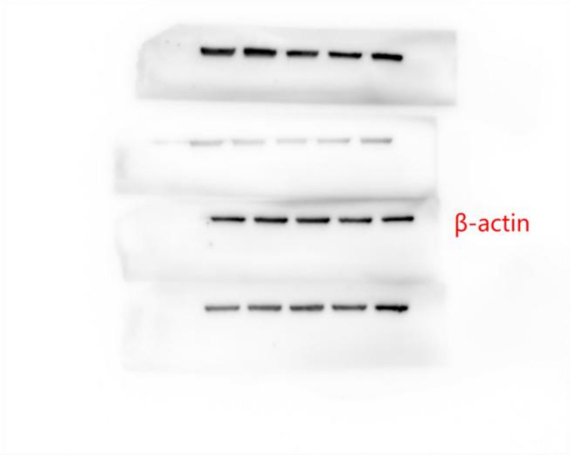

(E) The original western blot bands of Cleaved caspase-1, Cleaved GSDMD, IL-18, IL-1β, β-actin protein in PC12 cells. The lane order from left to right on blot is: (1) the Control group, (2) the CORT group, (3) the Si-NC + CORT group, (4) the Si-NLRP3 + CORT group, (5) the Sal + CORT group, (6) the Si-NLRP3 + CORT + Sal group.

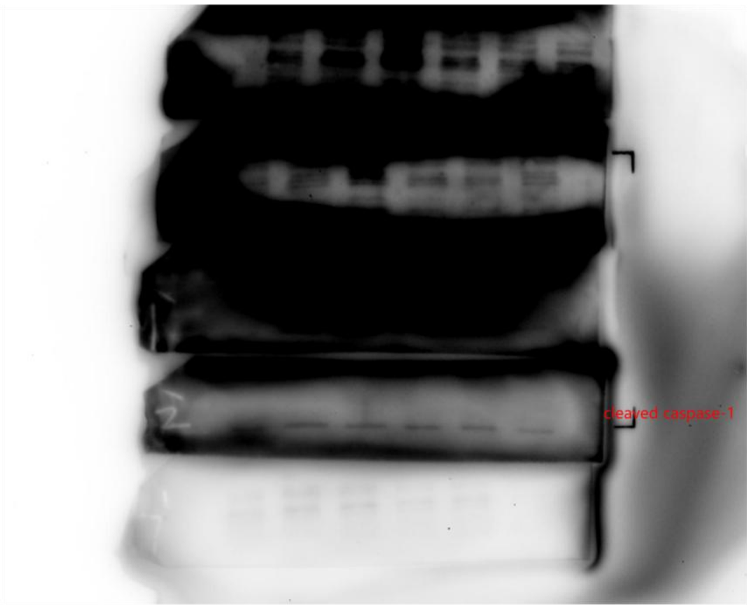
cleaved caspase-1

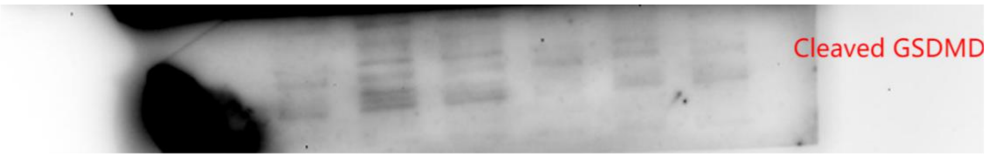
Cleaved GSDMD

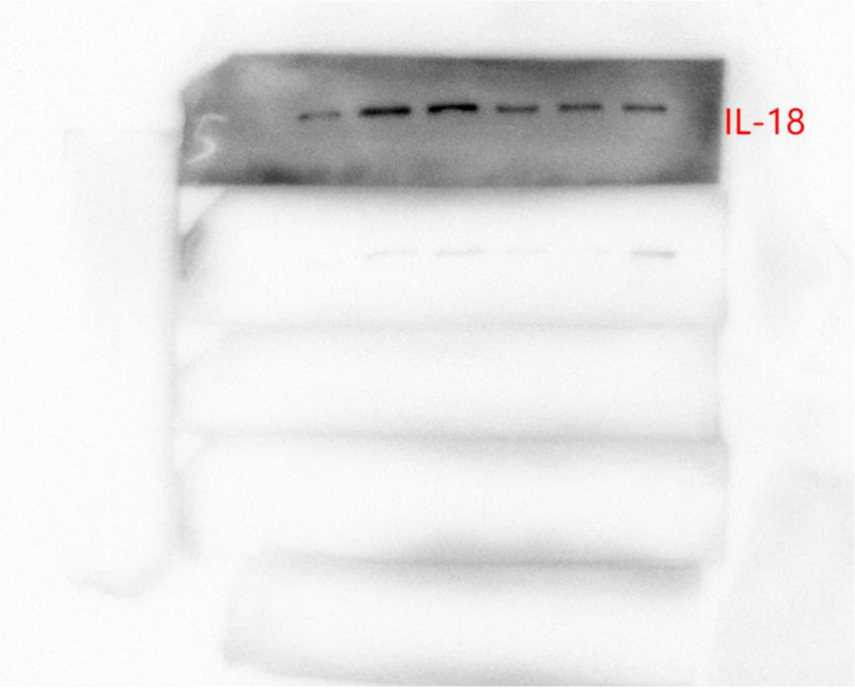
IL-18

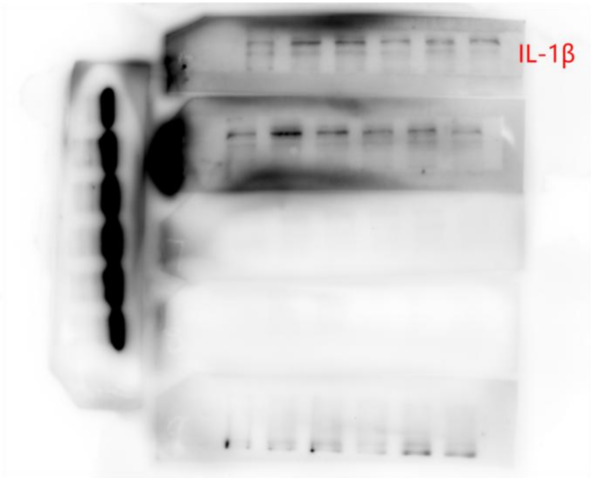
IL-1β

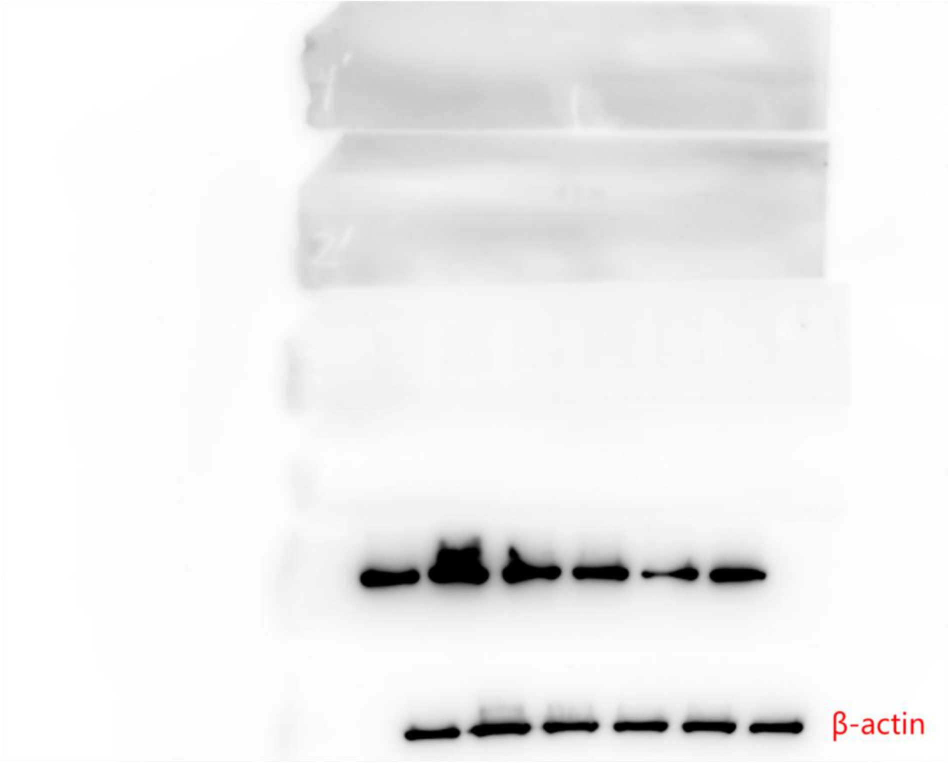
β-actin
