# Supplementary material for: Salidroside Ameliorates Depression by Suppressing NLRP3-Mediated Pyroptosis via P2X7/NF-κB/NLRP3 Signaling Pathway
Source: Front Pharmacol. 2022 Apr 12;13:812362. doi: 10.3389/fphar.2022.812362 (PMC9039222; doi:10.3389/fphar.2022.812362)
Supplement: Supplementary file 1 [file DataSheet1.ZIP › Supplementary Materials/Cover letter.docx]

November 10, 2021

To ***Frontiers in Pharmacology***

Dear editors:

Please find enclosed our manuscript entitled “**Salidroside ameliorates depression by suppressing NLRP3-mediated pyroptosis via P2X7/NF-κB/NLRP3 signaling pathway**”, which we would like to submit for publication as a Research Paper in ***Frontiers in Pharmacology*.** The work described has not been submitted elsewhere for publication, in whole or in part, and all the authors listed have approved the manuscript that is enclosed.

This study examines the underlying mechanisms of pyroptosis in depression and the neuroprotective effects of salidroside (a main active ingredient extracted from traditional herbal medicine). Our results indicated that inhibiting pyroptosis or administration of salidroside could be a novel therapeutic strategy for depression.

In this paper, two depression models, including Corticosterone and lipopolysaccharide, were established *in vivo* and Corticosterone-induced PC12 cell model *in vitro* to investigate the antidepressant actions of salidroside. Our results showed that salidroside successfully attenuated depression, as follows: (1) salidroside could improve depression-like behavior in the depression of mice. (2) salidroside ameliorates depression by inhibiting NLRP3-mediated pyroptosis in vitro and in vivo. (3) salidroside inhibits NLRP3-mediated pyroptosis by inhibiting the P2X7/NF-κB/NLRP3 signaling pathway. We believe that this manuscript is valuable for all researchers who are interested in depression.

We deeply appreciate your consideration of our manuscript and look forward to receiving comments from the reviewers. Everything goes well. Your kind considerations will be greatly appreciated.

Yours Sincerely,

Tianhua Yan, PhD, Professor

China Pharmaceutical University, Nanjing, China

Email: 1020050806@cpu.edu.cn
